# Texture-aware diffusion modelling for severity-conditioned synthetic progression-like visualization of diabetic foot ulcers from single images

**DOI:** 10.1371/journal.pone.0359540

**Published:** 2026-10-12

**Authors:** Pratham Kaushik, Vinay Kukreja, Eshika Jain, Modafar Ati, Shanmugasundaram Hariharan

**Affiliations:** 1 Centre for Research Impact and Outcome, Chitkara University Institute of Engineering and Technology, Chitkara University, Punjab, India; 2 Computer Science and Information Technology, College of Engineering, Abu Dhabi University, Abu Dhabi, United Arab Emirates; 3 Department of Computer Science and Engineering, Vel Tech Rangarajan Dr. Sagunthala R&D Institute of Science and Technology, Chennai, India; Islamia University of Bahawalpur: The Islamia University of Bahawalpur Pakistan, PAKISTAN

## Abstract

Diabetic foot ulcers (DFUs) are serious complications of diabetes associated with delayed healing, infection, and limb amputation. Modelling DFU progression remains challenging because publicly available image datasets generally contain independent single-timepoint images rather than longitudinal follow-up sequences. This study proposes a texture-aware conditional diffusion framework for generating severity-conditioned, progression-like synthetic DFU images from a single RGB input. Instead of claiming patient-specific temporal prediction, the framework models plausible population-level visual transitions across ordinal pseudo-stages derived from clinically relevant severity cues. The full model achieved an SSIM of 0.851 ± 0.020, PSNR of 24.76 ± 1.12 dB, and FID of 44.33 ± 5.02, with higher observed SSIM and PSNR and lower FID values than the evaluated GAN and VAE baselines across structural fidelity and distributional realism. The GAN baseline achieved SSIM 0.782 ± 0.031, PSNR 21.98 ± 1.56 dB, and FID 59.13 ± 6.59, while the VAE baseline achieved SSIM 0.738 ± 0.038, PSNR 19.63 ± 1.83 dB, and FID 69.77 ± 7.44. The framework also obtained a Texture Consistency Score of 0.79 ± 0.04 and a Granulation Tissue Fidelity Score of 0.76 ± 0.05, indicating preservation of fine-grained texture and granulation-related appearance patterns. Ablation results confirmed the contribution of texture conditioning and cross-attention to image fidelity. Advanced severity-state analysis produced SSIM values of 0.674, 0.804, and 0.726 across three pseudo-stages, supporting severity-aligned structural consistency. Since true longitudinal future-state images were unavailable, these metrics indicate synthetic image fidelity and distributional realism, not longitudinal progression accuracy. Longitudinal DFU cohorts and larger clinician-centred validation remain necessary before clinical decision-support use or temporal progression claims can be justified. To assess generalization beyond the primary 493-image dataset, external validation was additionally performed on an independent DFU dataset under a zero-shot setting, demonstrating stable cross-dataset performance with only modest degradation in image fidelity and texture consistency. Therefore, the proposed framework should be interpreted as modelling cross-sectional severity-conditioned appearance interpolation across independent cases, rather than true patient-specific future progression prediction.

## 1. Introduction

Diabetic foot ulcers (DFUs) are a critical and frequent complication of diabetes, frequently leading to chronic wounds, infection, and even limb amputation **if not identified and treated early**[1]. The precise forecasting of diabetic foot ulcer (DFU) progression and the risk of recurrence remains a major and complex **challenge for clinicians** and wound care experts. This challenge is due to the fact that traditional assessment techniques often **rely heavily on** subjective visual inspection or fixed risk factors that may differ significantly across practitioners and time. Consequently, these traditional methods may not provide sufficiently **accurate or reliable assessments**, which highlights the necessity of more accurate and objective instruments to enhance patient outcomes [2,3]. Recent computational methods have quickly improved the discipline, using machine learning and deep neural networks to automatically detect, stage, and predict the outcome of diabetic foot ulcers (DFUs). These advanced methods use a variety of imaging modalities, such as photographic, hyperspectral, and thermographic imaging, to increase diagnostic accuracy, allow earlier intervention, and improve patient outcomes. Consequently, they are becoming **an increasingly important part** of clinical practice, which can help healthcare professionals better **manage** this complex condition[4,5]. Machine learning algorithms that have been trained on a mix of both clinical data and image-based features, including Random Forests, Support Vector Machines (SVMs) and deep neural networks, can now accurately predict important outcomes **such as** ulcer healing, disease progression and recurrence. As an example, more sophisticated ML algorithms have reached classification rates as high as 92.5%, and cluster-based analysis has improved the performance of various DFU severity levels, such as mild to severe cellulitis and life- or limb-threatening infections [6,7]. These advanced predictive tools are increasingly valuable in personalized treatment planning and improving patient outcomes [1,8]. Asymmetries of texture and temperature have been found to be very effective in detecting pre-ulcerative conditions. Support Vector Machine (SVM) models have demonstrated **sensitivities exceeding 95%, indicating strong predictive performance** [9].

The analysis of texture has grown to be a more significant component of wound assessment over the last few years. It has been demonstrated that with a combination of well-designed, traditional texture features and deep learning-based features in combination with standard RGB images, wound healing classification and prognosis models can be significantly enhanced in accuracy. This combined method enables the healthcare professional to gain more insight into the nature of the wounds and possibly make more decisions regarding treatment [8,9].

Emerging deep learning models, such as convolutional neural networks (CNNs), well-tuned ensemble networks, and radiomics-based models are have shown promise in the field. They have demonstrated **strong performance**, particularly when explicit texture and shape features are analyzed and modelled. As an example, the EfficientNet architecture has reported a validation accuracy of 95% in the classification of healthy skin and ulcerated tissue, which underlines its possible integration into healthcare systems to **enable early detection and treatment of** ulcerated tissue [10,11]. Likewise, the Dense-ShuffleGCANet model, which is based on DenseNet, uses attention and channel-centric features to learn the heterogeneous visual properties of DFUs,achieving an accuracy of 86.09%, **which is higher than that of several** state-of-the-art architectures [12]. Moreover, ensemble models and hybrid architectures, including CNNs with support vector machines (SVMs) or K-nearest neighbors (KNNs), have also been shown to be more precise and robust in the classification of infection and ischemia in DFUs [4,13–15]. These advancements are gradually transforming the way we approach complex imaging and diagnostic tasks, providing more accurate and insightful results [16,17]. Recent advances also include the use of attention-driven networks, Siamese neural networks, and vision transformers, which enable multi-class classification and similarity learning for more nuanced DFU categorization, such as distinguishing between infection, ischemia, or both [18,19]. Segmentation models like Mask2former and U-Net have been applied for automated wound boundary detection and tissue classification, supporting detailed grading of ulcer severity and assisting clinicians in treatment planning [20,21]. In addition to mere detection, the clinical community is increasingly focused on **predicting progression and recurrence**. This can be achieved using both clinical and image-based predictors. Such approaches may support earlier interventions in informing earlier interventions and building more individualized care plans, ultimately leading to better patient outcomes and decreased risk of complications [22,23]. One recent breakthrough in the field is the use of advanced generative models, such as Generative Adversarial Networks (GANs) and, more recently, diffusion models. These models **are used to generate** synthetic wound images and the modelling of wound development with time. The diffusion models, specifically, have demonstrated impressive fidelity to generate images of Diabetic Foot Ulcers (DFUs), and research has shown that even as many as 70 percent of the synthetic images are viewed by clinical professionals as real, which makes them potentially useful in research, training, and diagnostic applications [24]. Notably, current developments in conditional diffusion models have significantly advanced the ability to predict infections from single wound images. These models have since surpassed most of the current state of the art approaches particularly in sensitivity and specificity, which is a significant breakthrough in medical imaging and diagnostic technology [25].

Synthetic medical imaging is currently undergoing a revolution with generative models, which can now not only be augmented to make rare wound classes but can also be simulated in silico to model disease progression. In particular, diffusion models have become an effective framework to create high-fidelity, realistic, and diverse synthetic images of diabetic foot ulcers [24]. These models have outperformed traditional GANs in terms of both realism and the preservation of clinically relevant microstructural details, as validated by wound experts [25]. Recent works also demonstrate the feasibility of using single images to predict future wound progression, leveraging both texture-aware analysis and computational modeling to forecast healing or deterioration [26,27].

Despite these advances, a major gap remains: existing models rarely integrate multi-scale, texture-aware encoding with robust, diffusion-based synthetic progression modelling from single photographic inputs. Addressing this gap, the present study introduces a texture-aware diffusion framework that integrates hand-crafted and deep texture features with conditional generative modelling to synthesize clinically interpretable, progression-like DFU appearances from a single input image. The proposed approach is positioned as a severity-conditioned visualization framework rather than a definitive temporal prediction system, thereby supporting risk-aware interpretation and methodological development under limited longitudinal data availability [14,28,29].

In response to this gap, the present study introduces a texture-aware conditional diffusion framework for severity-conditioned progression-like DFU image synthesis from a single input image. The framework combines local binary pattern descriptors and deep perceptual texture features with a cross-attention-enhanced diffusion architecture to preserve clinically relevant ulcer texture and morphology. Since the available datasets do not contain true longitudinal follow-up images, the proposed pseudo-progression strategy is not intended to represent deterministic patient-specific temporal forecasting. Instead, it provides a weakly supervised approximation of plausible population-level visual transitions across severity-aligned ordinal stages. This distinction is essential for ensuring that the proposed method is interpreted as progression-like synthetic visualization rather than definitive future ulcer prediction.

## 2. Contributions of the study

1. This study proposes a texture-aware conditional diffusion framework for generating severity-conditioned progression-like DFU images from a single RGB input, addressing the lack of longitudinal DFU image datasets while avoiding claims of deterministic patient-specific temporal prediction.
2. Introduces a hybrid texture encoding strategy combining local binary patterns and deep perceptual features, resulting in a Texture Consistency Score of 0.79 ± 0.04 and supporting fine-grained microstructural preservation during progression-like DFU synthesis.
3. Incorporates a cross-attention mechanism within the diffusion model, achieving an FID of 44.33 ± 5.02 and indicating improved alignment with real ulcer image distributions compared with GAN and VAE baselines.
4. Presents a Granulation Tissue Fidelity Score(GTFS), achieving 0.76 ± 0.05, indicating biologically plausible representation of granulation-related appearance patterns and supporting preliminary visual interpretability.
5. Performs detailed ablation studies in which the full model showed the most favorable observed metric values among the evaluated configurations, supporting the contribution of texture conditioning and cross-attention to progression-like synthesis quality.
6. Presents a timestep-wise severity-state analysis to examine how the model preserves structural and textural consistency across synthetic severity-aligned stages, with SSIM scores of 0.674, 0.804, and 0.726 across the evaluated stages.

## 3. Methodology

This paper introduces a diffusion-based synthetic generation framework that incorporates texture information to produce progression-like synthetic images of diabetic foot ulcers (DFUs) under different severity conditions. The methodology aims to generate visualisations of possible changes in the appearance of the ulcer from a single input image under weakly supervised conditions, especially in the absence of longitudinal follow-up datasets. The entire pipeline combines image preprocessing, texture feature encoding, and a conditional diffusion architecture to generate morphologic and textural variations at hypothetical pseudo-stages that are aligned with the severity. Each of the phases of the proposed approach is explained in detail in the following subsections, and the details of data collection, preprocessing strategies, texture encoding techniques, model architecture design, training strategies, evaluation protocols, and implementation specifics are shown in Fig 1.

**Fig 1:**
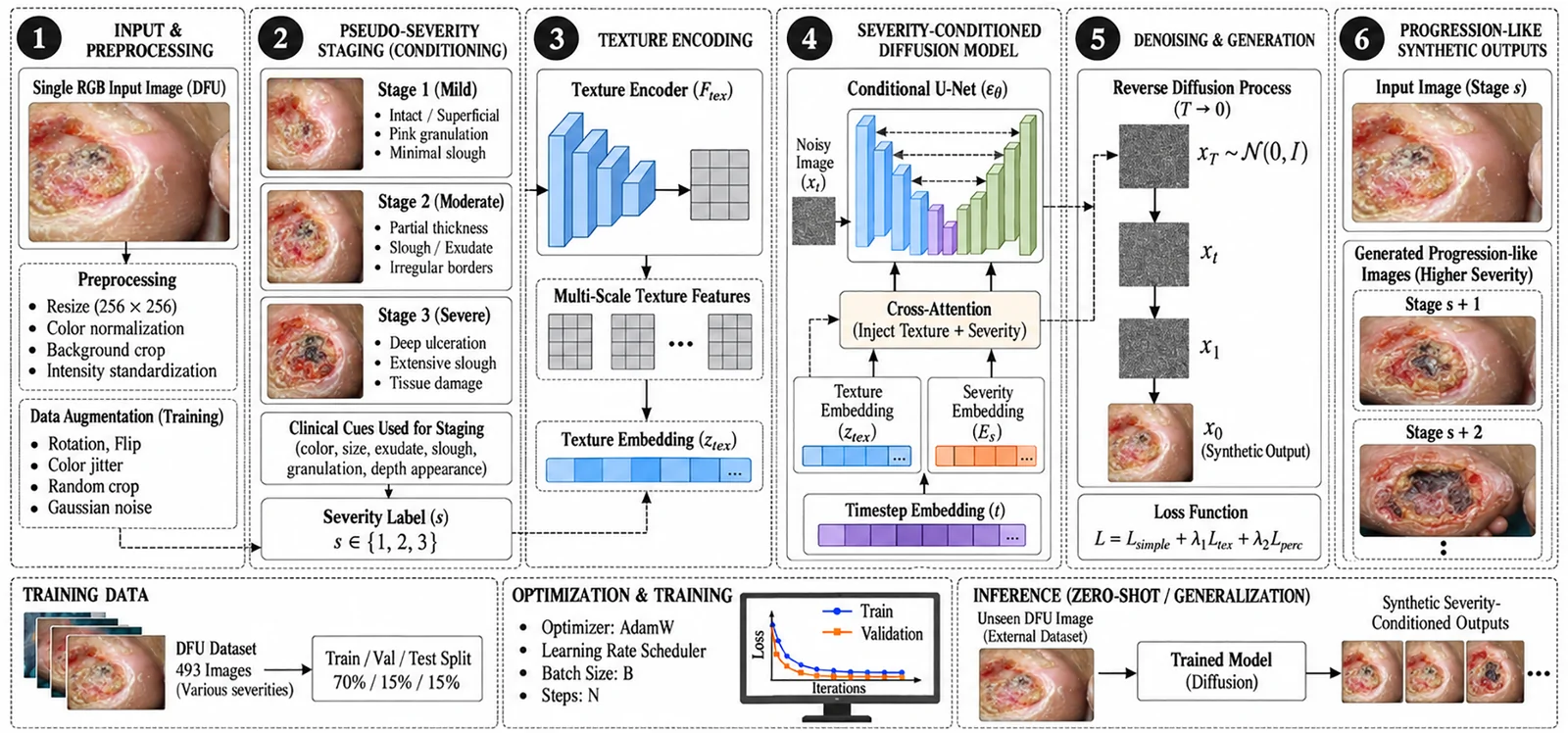
Proposed texture-aware severity-conditioned diffusion synthesis framework for DFUs.

### 3.1 Phase 1: Data collection

In this study, a publicly available dataset comprising 493 high-resolution RGB images of diabetic foot ulcers (DFUs) was obtained from Kaggle [30], as seen in Fig 2. All images show a different type of ulcer, with a wide spectrum of ulcer severity and tissue changes from granulation to necrosis and epithelialization. Images were taken in different clinical settings, and therefore under a variety of lighting conditions, foot position and ulcer morphology. No longitudinal follow-up images were available, so each image was considered as a separate case. The dataset was adequate for modelling ulcer texture patterns and morphological characteristics that could be used to create synthetic images of the DFU that mimic progression. However, the dataset did not contain repeated observations of the same ulcer over time, so direct supervision of true ulcer progression over time was not possible.. Thus, pseudo-stages were constructed from cross-sectional severity cues rather than from temporal wound trajectories, to create severity-aligned pseudo-stages. This design decision is recognized as a methodological limitation and the scope of the proposed framework is limited to a progression-like synthesis based on severity, not to a prediction of the future state. The basic dataset is a collection of 493 photos of diabetic foot ulcers from a public Kaggle repository. The data set was split into 345 images used for training, 74 images used for validation and 74 images used for testing (70%, 15%, 15%). There was no overlap among the training, validation, and test subsets, and the split was performed at the image level using a fixed random seed for reproducibility. A fixed train–validation–test split was used in all the baseline, ablation, and comparative experiments to provide fair and consistent model evaluation under the same data conditions. Patient-wise splitting was not possible as patient identifiers were unavailable in the dataset; this is acknowledged as a limitation of the publicly available DFU image collection. It is also noted that the relatively small size of the primary dataset is an important constraint when training a diffusion-based generative model. To prevent overfitting, the same preprocessing, data augmentation, data splitting and evaluation methods were used for all the model variants. However, the small sample size limits the representativeness of the diversity of the DFU presentations and is thus a methodological limitation. It is important to note that in this study the independent single-timepoint images do not offer temporal supervision. Therefore, the severity pseudo-stages should not be used as chronological stages of the same ulcer. Rather, they are cross-sectional severity groupings of independent case based on visual features of the ulcer appearance including size, depth, prevalence of granulation tissue and necrotic coverage. Hence, the pseudo-stages should be interpreted as appearance interpolations that are conditioned by the severity, not as temporal sequences. This definition clarifies the dataset is appropriate for severity-conditioned progression-like synthesis but not for true longitudinal progression prediction.

**Fig 2:**
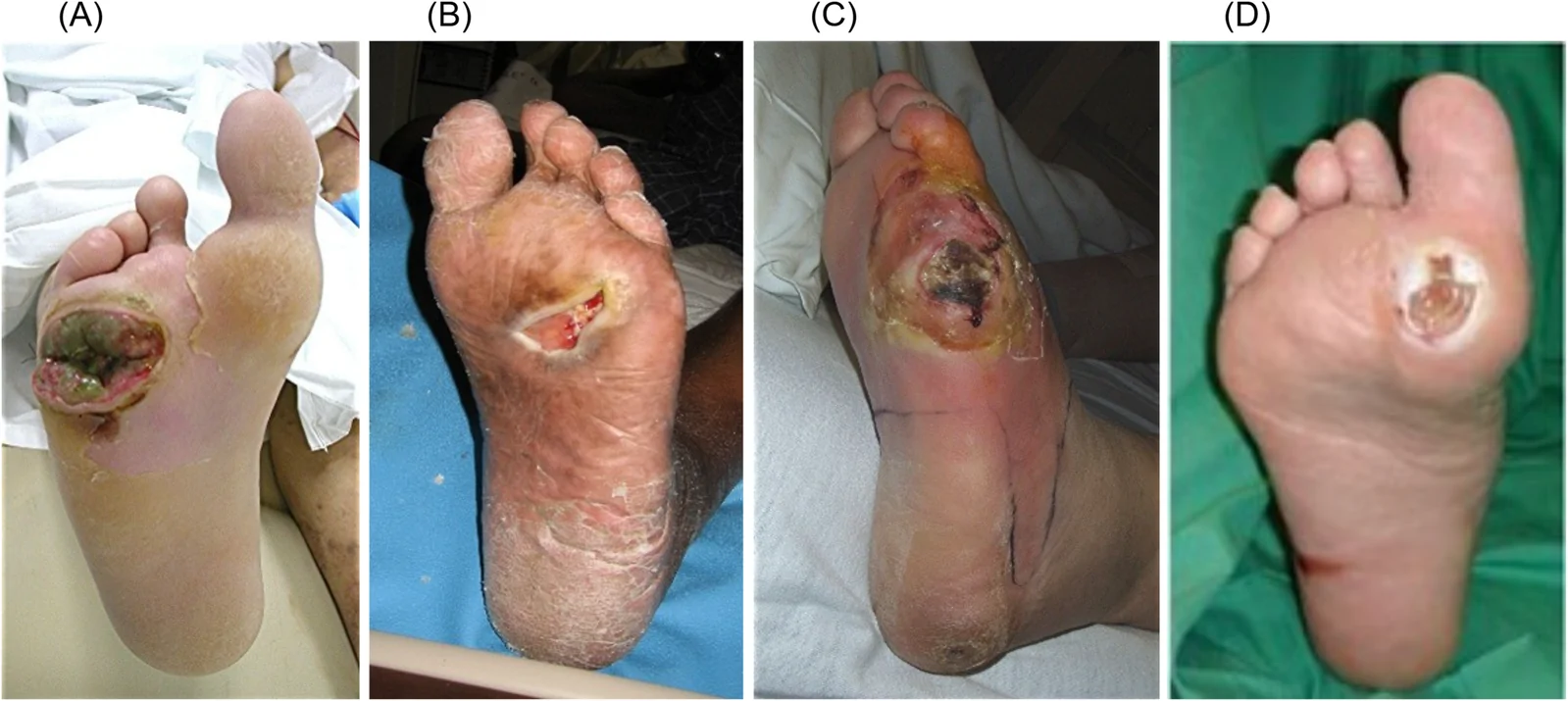
Sample images from the dataset [30].

### 3.2 Phase 2: Data preprocessing

An advanced preprocessing pipeline was used to create a high-fidelity and robust input representation for the diffusion model, going beyond the standard resizing and simple augmentations. All images were first resized to a common resolution of 256 × 256 pixels using aspect-preserving interpolation to minimize geometric distortion, while preserving the structural integrity of ulcer boundaries. Then adaptive background subtraction was applied to the image “using morphological operations and dynamic thresholding to extract the ulcer area, which effectively reduced the interference of background noise and irrelevant anatomical information.

To enhance intra-class texture variance while preserving fine-grained morphological cues, a multi-scale texture enhancement technique was introduced. Specifically, for each image *I*, a set of Gaussian-filtered versions $\left\{G_{\sigma }(I)\right|\;\sigma \in \{\text{1},\text{2},\text{4}\}\}$ was computed, and a composite texture-enhanced image $I_{T}$ was formulated as equation 1:


$$\begin{array}{c} \begin{array}{c} I_{T}=I+\sum_{\sigma }\alpha_{\sigma }\left(I-G_{\sigma }(I)\right), \end{array} \end{array}$$ (1)

Where $\alpha_{\sigma }$ denotes a learnable weight controlling the contribution of each scale, thus accentuating critical textural discontinuities associated with granulation and necrotic spread.

Furthermore, to standardize intensity distributions and compensate for acquisition variations, histogram specification was performed to align each image histogram $H_{I}$ to a canonical reference histogram $H_{R}$ derived from a severity-aligned reference subset, mathematically expressed as equation 2:


$$\begin{array}{c} \begin{array}{c} I^{\prime }=H_{I}^{-\text{1}}\left(H_{R}(I)\right), \end{array} \end{array}$$ (2)

Where $H_{I}^{-\text{1}}$ denotes the inverse cumulative distribution function of the source histogram.

Finally, a context-preserving elastic deformation was incorporated to simulate realistic variations in skin stretching and ulcer expansion, parametrized by a displacement field D(x,y)=(Δx,Δy) governed by a random smooth Gaussian field, enabling the model to learn invariant representations under physiologically plausible transformations. These advanced preprocessing strategies collectively enriched the dataset, providing a diverse and texture-preserving foundation critical for effective progression-like synthesis as seen in Fig 3.

**Fig 3:**
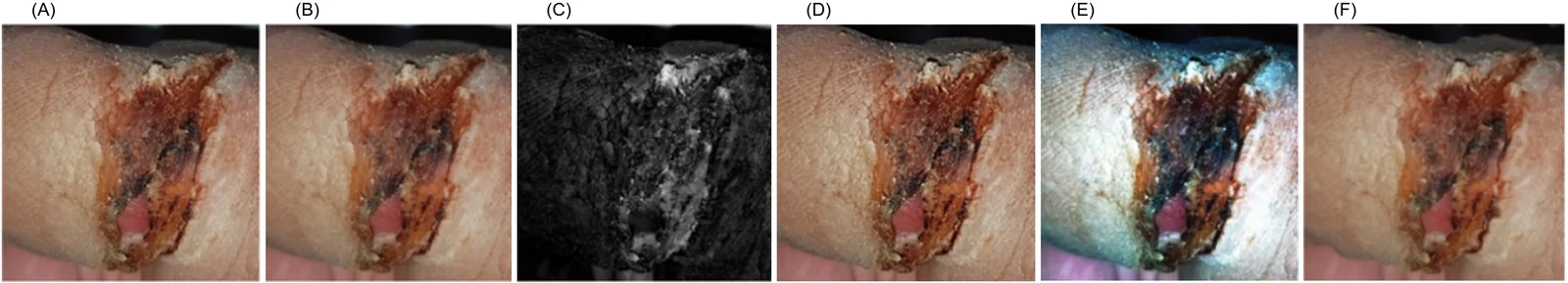
Preprocessing step for ulcer images.

### 3.3 Phase 3: Texture feature encoding

Following the advanced preprocessing strategies described in Phase 2, a dedicated texture feature encoding scheme was designed to capture both fine-grained microstructural details and global morphological patterns crucial for modelling severity-associated DFU appearance variation. Each pre-processed and texture-enhanced image, denoted as $I_{T^{\prime }}^{\prime }$ served as the foundation for constructing a multi-scale, texture-aware representation that informs the diffusion-based generative model.

Initially, local texture variations and severity-associated indicators of ulcer appearance, such as granulation tissue spread, slough formation, and necrotic changes, were quantitatively encoded using Local Binary Patterns (LBP). For each pixel *p*, the LBP code LBP(p) was derived as follows in equation 3:


$$\begin{array}{c} \begin{array}{c} LBP(p)=\sum_{n=\text{0}}^{P-\text{1}}s\left(i_{n}-i_{p}\right)\;X\;{\text{2}}^{n},\;s(x)=\left\{ \begin{array}{cc} \text{1} & x\geq \text{0},\\ \text{0} & x<\text{0}, \end{array}\right.\; \end{array} \end{array}$$ (3)

Where $i_{p}$ represents the intensity at the central pixel and $i_{n}$ denotes the intensity values of its *P* equally spaced neighbourhood pixels. This formulation captures local texture transitions, essential for differentiating healthy tissue from early signs of ischemia or infection. To supplement these local features with higher-level semantic and structural information, deep perceptual features $F_{VGG}(I_{T}^{\prime })$ were extracted using intermediate convolutional layers from a pre-trained VGG-19 network. This process can be mathematically represented as equation 4:


$$\begin{array}{c} \begin{array}{c} F_{VGG}\left(I_{T}^{\prime }\right)=\left\{f_{l}\left(I_{T}^{\prime }\right)|l\in L\right\}, \end{array} \end{array}$$ (4)

Where $f_{l}$(.) signifies the feature activation map at layer *l*, and L is a carefu*ll*y selected set of layers capturing hierarchical texture and shape characteristics. These perceptual features enable the model to understand the ulcer’s broader morphological context, such as edge irregularity and peripheral tissue degradation. To harmonize and integrate the fine-scale LBP features with the deep perceptual features, a unified texture descriptor $F_{T}$ was constructed. This was achieved through feature normalization and channel-wise concatenation, followed by a learnable linear projection $W_{T}$, formulated as equation 5:


$$\begin{array}{c} \begin{array}{c} F_{T}=W_{T}\left[Norm\left(LBP\left(I_{T}^{\prime }\right)\right),\;Norm\left(F_{VGG}\left(I_{T}^{\prime }\right)\right)\right], \end{array} \end{array}$$ (5)

where Norm (⋅) denotes feature-wise normalization to ensure scale invariance and stable downstream conditioning. The resulting composite feature map $F_{T}$ was appended as an auxiliary conditioning input to the diffusion model, providing explicit guidance to preserve critical ulcer textures and morphological details during progression-like synthesis.

By integrating local texture encodings and high-level semantic features into a unified, learnable representation, this phase ensures that the generative diffusion process not only maintains visual fidelity but also respects subtle biological cues associated with ulcer severity and tissue appearance. Consequently, the generated progression-like synthetic images exhibit high visual and clinical plausibility, making them useful for exploratory visualization and risk-aware interpretation, as seen in Fig 4.

**Fig 4:**
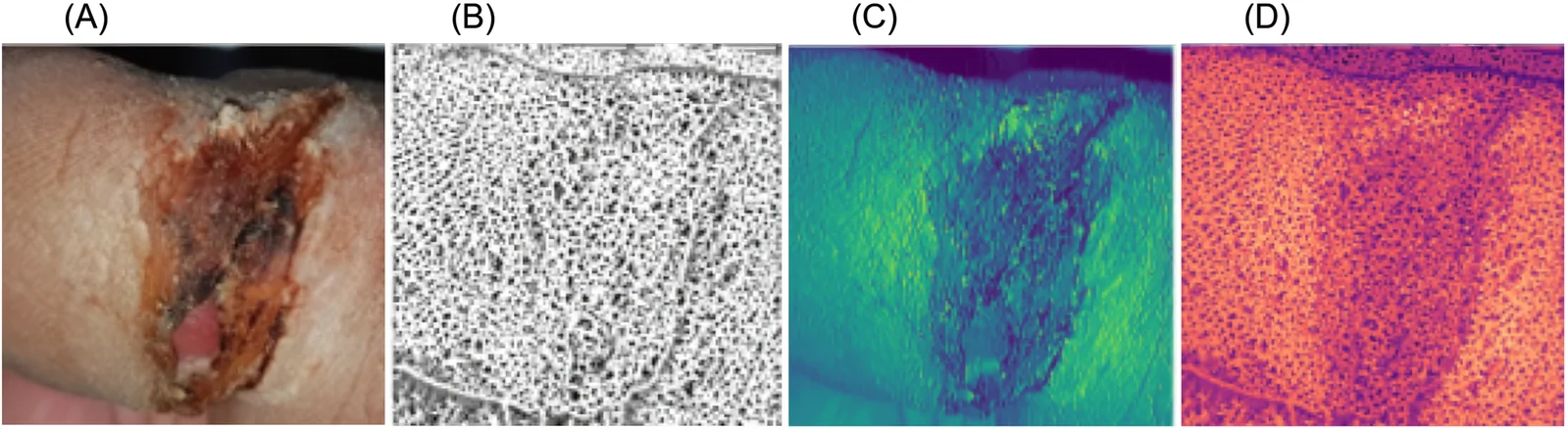
Texture feature extraction and composite descriptor visualization.

### 3.4 Phase 4: Diffusion model architecture

Building upon the unified texture representation constructed in Phase 3, a specialized conditional diffusion probabilistic model was designed to generate severity-conditioned progression-like synthetic DFU images from a single input image. The core framework is grounded in the denoising diffusion probabilistic model (DDPM) paradigm, which iteratively refines a noise-initialized latent representation towards a high-fidelity synthetic image by progressively reversing a defined noise corruption process. Mathematically, the forward diffusion process corrupts an input image $x_{\text{0}}$ by adding Gaussian noise across *T* time steps, formulated as equation 6:


$$\begin{array}{c} \begin{array}{c} q\left(x_{t}|x_{t-\text{1}}\right)=\;N\left(x_{t};\;\sqrt{\text{1}-\beta_{t}}\;x_{t-\text{1}},\beta_{t}I\right), \end{array} \end{array}$$ (6)

Where $x_{t}$ denotes the intermediate noisy image at step *t*, $\beta_{t}$ represents the variance schedule parameter, and *I* is *t*he identity matrix. The reverse denoising process, parameterized by a neural network $\epsilon_{\theta }$, estimates the noise component and reconstructs the clean image from the corrupted state as equation 7:


$$\begin{array}{c} \begin{array}{c} p_{\theta }\left(x_{t-\text{1}}|x_{t},F_{T}\right)=𝒩\left(x_{t-\text{1}};\mu_{\theta }\left(x_{t},t,F_{T}\right),\sum_{\theta }\left(x_{t},t,F_{T}\right)\right), \end{array} \end{array}$$ (7)

Where $F_{T}$ is the composite texture feature map introduced as a conditional input, ensuring preservation of ulcer-specific micro-textural and macro-morphological cues throughout the generative trajectory. The model backbone is implemented as a U-Net architecture equipped with cross-attention mechanisms, facilitating the propagation of multi-scale features and enabling dynamic focus on critical ulcer regions. In each decoding layer, cross-attention modules integrate the texture encoding $F_{T}$ by learning attention weights α that highlight clinically relevant sub-regions, mathematically expressed as equation 8:


$$\begin{array}{c} \begin{array}{c} Attention(Q,K,V)=softmax\left(\frac{QK^{T}}{\sqrt{d_{k}}}\right)V, \end{array} \end{array}$$ (8)

where queries *Q*, keys *K*, and values *V* are derived from the intermediate U-Net feature maps and the projected texture embeddings, and $d_{k}$ denotes the dimension of the key vectors. This design allows the model to adaptively focus on ulcer structural patterns and their complexity, which may be associated with ulcer severity. Furthermore, a hierarchical skip-connection approach is used to retain low-level spatial details and capture high-level semantic appearance variations, which ensures structural coherence and textural realism of the final synthetic outputs. The diffusion model was additionally extended with an auxiliary feature-consistency loss that is used to enforce the consistency of the intermediate features of the generated images with the features of the original input image, which is important for encouraging biological plausibility in the progression-like synthetic output. The proposed architecture incorporates a texture-conditioned cross-attention mechanism into a powerful diffusion model to tackle the problem of modelling plausible changes in DFU appearance from a single image when considering severity. This allows for the creation of visually interpretable severity-consistent progression-like visualizations that could aid in exploratory risk-aware interpretation, although further validation would be required before clinical use , as shown in Fig 5.

**Fig 5:**
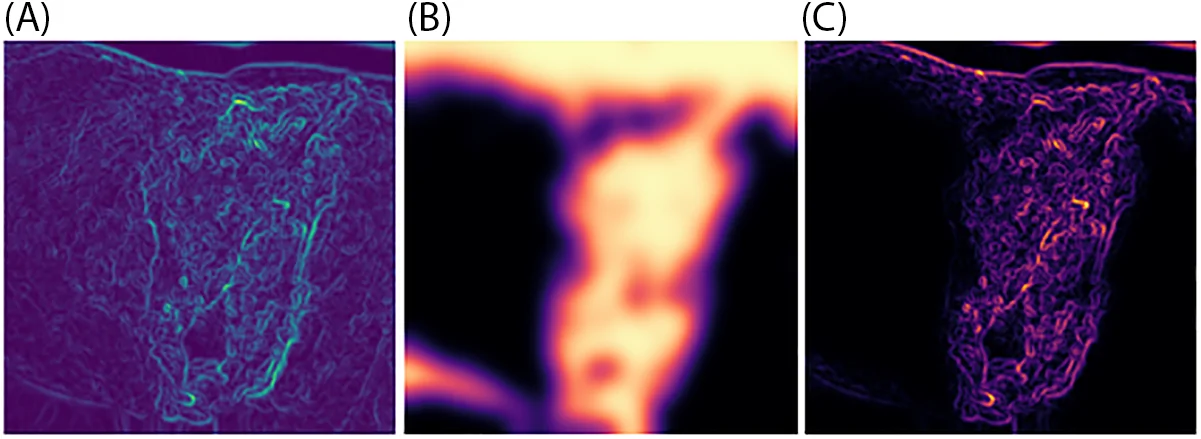
Feature attention integration within diffusion model architecture.

### 3.5 Phase 5: Training strategy

A multi-objective training strategy was carefully designed to effectively learn the complex mappings needed for realistic synthetic images that resemble severity-associated progression-like appearances of diabetic foot ulcers (DFUs). The main goal was to teach the conditional diffusion model to generate high fidelity progression-like DFU images, while maintaining global morphology and fine-grained textural features corresponding to pseudo-stages that reflect severity. However, no real longitudinal images of ulcers were available and a pseudo-progression paradigm was therefore used. This method involved the categorisation of images and the approximate classification into ordinal pseudo-stages using visual indicators of severity such as ulcer size, depth, granulation presence and necrotic coverage. Pseudo-stage labels were defined using a predefined heuristic, appearance-based severity grouping scheme derived from cross-sectional visual characteristics of the ulcer region. Stage 1 (mild) represented predominantly superficial ulcer appearance with pink granulation and minimal slough; Stage 2 (moderate) represented partial-thickness appearance with visible slough or exudate and irregular wound borders; and Stage 3 (severe) represented deeper ulceration with extensive slough and more pronounced tissue damage. Supporting cues included ulcer size, colour, apparent depth, granulation appearance, exudate/slough, and necrotic coverage. These pseudo-stage labels were used only as weak severity-conditioning variables and were not derived from longitudinal follow-up, patient outcomes, or a formal clinician-led annotation or adjudication protocol. Accordingly, they should be interpreted as heuristic severity groupings rather than clinically validated wound grades or temporal progression labels.

The pseudo-progression paradigm should be viewed as a weakly supervised severity-conditioned ordering paradigm, and not as a temporal supervision paradigm. The available DFU images are cross-sectional and do not represent repeated visits from the same patients., so the model does not learn patient-specific wound evolution over chronological time. Instead, it learns population level visual transitions corresponding to the increase or change in ulcer severity. The outputs are thus not predictions of the clinical state of a particular ulcer, but rather plausible appearance-like progressions given the severity-related texture and morphology of the ulcer. The term pseudo-progression is used in this study to denote the severity conditioned ordinal ordering of independent DFU images. It does not indicate genuine temporal progression, patient-level follow-up, or future-state supervision. The pseudo-progression strategy was adopted only as a practical workaround for limited longitudinal data availability. Since the ordinal stages are derived from cross-sectional images belonging to different cases, the training process models severity-associated appearance interpolation across independent patients rather than true patient-specific temporal progression. Accordingly, the generated outputs are described as severity-conditioned progression-like synthetic visualizations, not as future progression predictions.

The training process optimized a composite loss function, formulated as equation 9:


$$\begin{array}{c} \begin{array}{c} {ℒ}_{total}=\lambda_{\text{1}}{ℒ}_{rec}+\lambda_{\text{2}}{ℒ}_{perc}+\lambda_{\text{3}}{ℒ}_{adv}+\lambda_{\text{4}}{ℒ}_{feat}, \end{array} \end{array}$$ (9)

where λ_1_, λ_2_, λ_3_, and λ_4_ are weighting coefficients empirically set to balance the contribution of each loss term. The reconstruction loss $L_{rec}$ enforces pixel-wise fidelity between the input image $x_{\text{0}}$ and the generated image ${\hat{x}}_{\text{0}}$, and is defined as equation 10:


$$\begin{array}{c} \begin{array}{c} {ℒ}_{rec}={\left\|x_{\text{0}}-{\hat{x}}_{\text{0}}\right\|}_{\text{2}}^{\text{2}} \end{array} \end{array}$$ (10)

The perceptual loss $L_{perc}$ leverages intermediate feature activations from a pre-trained VGG-19 network to preserve high-level semantic and textural similarity, expressed as equation 11:


$$\begin{array}{c} L_{perc}=\sum_{l\in L}{\left\|\phi_{l}\left(x_{\text{0}}\right)-\phi_{l}({\hat{x}}_{\text{0}})\right\|}_{\text{2}}^{\text{2}}\;,\; \end{array}$$ (11)

where $\phi_{l}$ (.) denotes the feature map extracted from layer l, and L represents the selected set of VGG-19 layers used for perceptual feature comparison. The adversarial loss $L_{adv}$ encourages photorealistic texture synthesis by employing a patch-based discriminator D, defined as equation 12:


$$\begin{array}{c} L_{adv}=E_{x}[\text{log}D(x_{\text{0}})]+E_{\hat{x}}[\text{log}(\text{1}-D\left({\hat{x}}_{\text{0}}\right))] \end{array}$$ (12)

Also, to maximize the consistency of the features, an extra feature consistency loss $L_{feat}$ is introduced that matches the intermediate features of the generator with those of the input image. This term encourages consistency in the texture representation and biologically plausible progression-like texture synthesis by enforcing consistency between the generated outputs and the feature representation derived from the input. The Adam optimizer with a learning rate of 10^-4^, weight decay of 10^-5^, and a batch size of 8 was used to train. The learning rate was gradually decreased using a cosine annealing scheduler for smoother convergence in later training stages. To enhance robustness to clinical variability and to aid in generalization across unseen ulcer presentations, extensive data augmentation was performed during the training process, such as elastic deformation and intensity perturbations. To ensure smoother iterative denoising steps in the synthesis process, an exponential moving average (EMA) of the model weights was kept with a decay rate of 0.999 to further stabilize the diffusion learning process. The training was conducted on an NVIDIA RTX 3090 GPU with convergence taking about 40 hours.

Texture conditioning, perceptual loss, and feature-consistency loss were incorporated to minimize texture degradation during synthesis. However, residual smoothing of very-high-frequency boundary details remained visible in the zoomed-in analysis, indicating the need for explicit frequency-aware and boundary-aware texture-refinement strategies in future work.

This thorough multi-objective optimization approach allowed the model to be taught detailed patterns of ulcer appearance, while simultaneously minimizing the structural deviations while maximizing the textural realism. Several measures were also incorporated to reduce overfitting during training, including extensive data augmentation, weight decay, cosine learning-rate scheduling, exponential moving average of model weights, and validation-based monitoring. These strategies were used to improve training stability and generalization under limited data availability. Nevertheless, the dataset size remains modest for diffusion-based generative modelling, and external validation was therefore included to further examine cross-dataset robustness.

The observed residual smoothing of high-frequency boundary details suggests that future training objectives should extend beyond perceptual and feature consistency losses by incorporating frequency-aware losses, high-pass adversarial supervision, and boundary-level patch refinement to explicitly preserve micro-textural structures, as seen in Fig 6.

**Fig 6:**
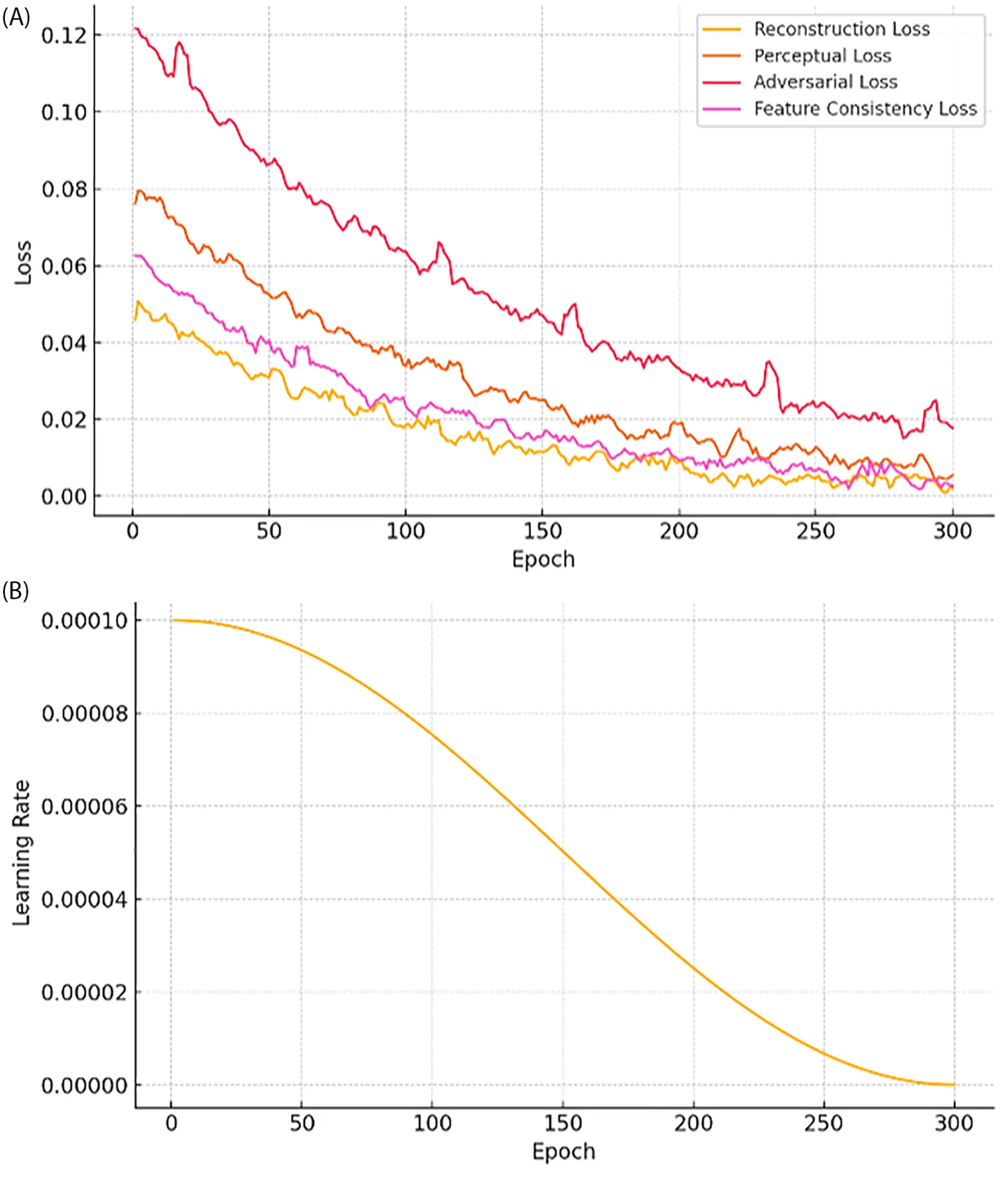
Training dynamics: loss curves and learning rate schedule.

### 3.6 Phase 6: Evaluation and validation

To evaluate the technical quality and visual plausibility of the progression-like synthetic diabetic foot ulcer images generated by the proposed diffusion model, an evaluation procedure was used that integrated quantitative image-quality metrics, qualitative visual inspection, ablation analysis, and robustness assessment. Quantitative evaluation was conducted using two established image quality metrics: the Structural Similarity Index Measure (SSIM) and the Fréchet Inception Distance (FID). The SSIM metric, defined as equation 13:


$$\begin{array}{c} \begin{array}{c} SSIM(x,y)=\frac{\left(\text{2}\mu_{x}\mu_{y}+c_{\text{1}})(\text{2}\sigma_{xy}+c_{\text{2}}\right)}{{(\mu }_{x}^{\text{2}}+{(\mu }_{y}^{\text{2}}+c_{\text{1}})(\sigma_{x}^{\text{2}}+\sigma_{y}^{\text{2}}+c_{\text{2}})} \end{array} \end{array}$$ (13)

Where $\mu_{x}$ and $\mu_{y}$ represent mean intensities, $\sigma_{x}^{\text{2}}$ and $\sigma_{y}^{\text{2}}$ denote variances, $\sigma_{xy}$ is the covariance between images *x* and *y*, and $c_{\text{1}}$, $c_{\text{2}}$ are stabilizing constants, which were used to evaluate perceptual similarity between generated images and reference pseudo-progression samples. High SSIM values indicate strong structural fidelity and textural coherence, which are essential for clinical interpretability as seen in Fig 7.

**Fig 7:**
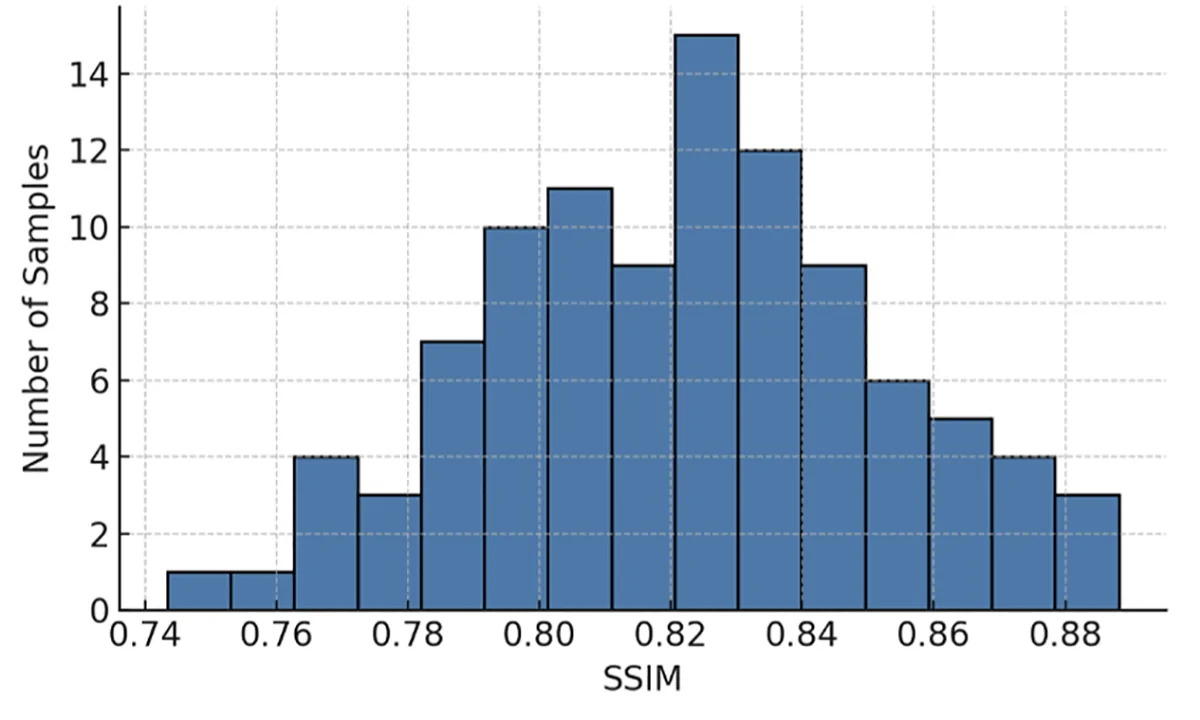
Distribution of SSIM Scores Across Test Samples.

FID was employed to quantify the distributional similarity between the generated image set and the real dataset, calculated as equation 14:


$$\begin{array}{c} \begin{array}{c} FID=\left(X_{r},\;X_{g}\right)={\left\|\mu_{r}-\mu_{g}\right\|}_{\text{2}}^{\text{2}}+Tr\left(\sum_{r}+\sum_{g}-\text{2}{\left(\sum_{r}\sum_{g}\right)}^{\text{1}/\text{2}}\right), \end{array} \end{array}$$ (14)

where ($\mu_{r},\;\sum_{r}$) and ($\mu_{g},\;\sum_{g}$) represent the means and covariance matrices of features extracted from a pre-trained Inception network for real ($X_{r})$ and generated ($X_{g})$ images, respectively. Lower FID scores indicate higher fidelity and better realism. In parallel, qualitative visual inspection was performed to assess the visual plausibility of the generated progression-like synthetic images. The generated outputs were examined for morphological coherence, texture continuity, ulcer-boundary preservation, and the absence of visually unrealistic artefacts. This qualitative inspection was used only to support technical interpretation of the generated images and should not be interpreted as clinician-based validation, diagnostic evaluation, or evidence of clinical decision-support effectiveness. Since no formal specialist review was conducted, no Likert-scale scoring or inter-rater reliability analysis using Cohen’s kappa was performed.

Moreover, visual interpretability was also enhanced by the creation of pixel-level attention maps based on the cross-attention modules of the model, which identified areas most important in generating progression-like synthetic outputs. These attention overlays supported the straightforward interpretation of the clinical aspects, as certain parts of the ulcer and its texture that contributed to the formed outcomes were highlighted. To supplement the quantitative and qualitative visual analyses, an ablation study was conducted to examine the importance of the main architectural components, including the texture conditioning module and cross-attention mechanisms. Comparisons of metrics such as SSIM and FID were made between the variants of the models with and without each of these components, thereby confirming the importance and effect of each design element on the realism of progression-like synthesis. Because no ground-truth future DFU images were available, the evaluation metrics were not used to measure true longitudinal progression accuracy. SSIM and PSNR were used to assess structural and pixel-level similarity between generated outputs and severity-aligned pseudo-progression references. FID was used to measure distributional similarity between generated images and the real DFU image dataset. Therefore, these metrics quantify synthetic image fidelity, texture preservation, and dataset-level realism, rather than chronological progression accuracy or patient-specific future-state prediction. In addition to established image-quality metrics, two task-specific supplementary metrics, namely the Texture Consistency Score (TCS) and Granulation Tissue Fidelity Score (GTFS), were used to evaluate clinically relevant appearance characteristics that are not fully captured by generic image similarity measures. TCS was designed to assess local micro-textural preservation using LBP-based texture similarity, while GTFS was used to estimate the consistency of granulation-related appearance patterns between generated images and algorithmically derived severity-aligned pseudo-reference appearance maps. These metrics were not intended to replace established evaluation standards such as SSIM, PSNR, FID, clinician-led assessment, or prospective clinical validation. Instead, they were used as complementary exploratory indicators for assessing texture preservation and granulation-related visual plausibility in progression-like DFU synthesis.

Since the pseudo-stages are derived from cross-sectional severity grouping, the evaluation assesses synthetic image fidelity and severity-conditioned consistency, not temporal forecasting accuracy.

#### TCS and GTFS formal definition.

To improve reproducibility, the Texture Consistency Score (TCS) and Granulation Tissue Fidelity Score (GTFS) were formally defined and computed using explicit algorithmic steps. These metrics were designed as task-specific supplementary indicators for evaluating texture preservation and granulation-related appearance fidelity in severity-conditioned progression-like DFU synthesis. They were not used as standardized clinical validation metrics.

For each generated progression-like image ${\hat{I}}_{i}$and its corresponding severity-aligned pseudo-reference image $I_{i}^{r}$, Local Binary Pattern (LBP) maps were first extracted using the same LBP configuration described in the texture encoding stage. The corresponding LBP histograms were then normalized to obtain $H^{LBP}({\hat{I}}_{i})$and $H^{LBP}(I_{i}^{r})$. Texture dissimilarity between the generated image and the pseudo-reference image was calculated using the chi-square histogram distance, as expressed in equation 15:


$$\begin{array}{c} \begin{array}{c} D_{\chi^{\text{2}}}^{\left(i\right)}=\frac{\text{1}}{\text{2}}\sum_{b=\text{1}}^{B}\frac{{\left(H_{b}^{LBP}({\hat{I}}_{i})-H_{b}^{LBP}(I_{i}^{r})\right)}^{\text{2}}}{H_{b}^{LBP}({\hat{I}}_{i})+H_{b}^{LBP}(I_{i}^{r})+\varepsilon } \end{array} \end{array}$$ (15)

where $D_{\chi^{\text{2}}}^{\left(i\right)}$ denotes the chi-square texture dissimilarity for the $i^{th}$ image pair, B represents the total number of LBP histogram bins, bdenotes the histogram-bin index, and ε is a small numerical constant added to avoid division by zero. A lower value of $D_{\chi^{\text{2}}}^{\left(i\right)}$ indicates stronger similarity between the local texture distributions of the generated image and the severity-aligned pseudo-reference image.

The image-level Texture Consistency Score was then obtained by applying exponential normalization to the chi-square distance, as shown in equation 16:


$$\begin{array}{c} \begin{array}{c} TCS_{i}=\text{exp}\left(-D_{\chi^{\text{2}}}^{\left(i\right)}\right) \end{array} \end{array}$$ (16)

where $TCS_{i}$ represents the texture consistency score for the $i^{th}$ generated image. This transformation maps the texture dissimilarity into a bounded similarity score, where values closer to 1 indicate stronger preservation of local micro-textural patterns.

The final Texture Consistency Score was calculated by averaging the image-level TCS values across all N test samples, as expressed in equation 17:


$$\begin{array}{c} \begin{array}{c} TCS=\frac{\text{1}}{N}\sum_{i=\text{1}}^{N}TCS_{i} \end{array} \end{array}$$ (17)

where N denotes the total number of evaluated test samples. Higher TCS values indicate stronger preservation of fine-grained texture patterns, including local tissue roughness, keratinization-like appearance, and necrosis-like texture variation, between generated progression-like images and severity-aligned pseudo-reference images.

#### Derivation of the granulation-related appearance map.

The granulation-related appearance map G(I) was implemented as a deterministic, non-learned soft colour-appearance map rather than as the output of a trained tissue-segmentation network. For each preprocessed RGB image I, analysis was restricted to the ulcer-region mask Ωobtained using the same adaptive background-subtraction and dynamic-thresholding procedure described in Section 3.2. The pixels within Ωwere converted from RGB to the CIELAB colour space. Let $L^{*}(p)$, $a^{*}(p)$, and $b^{*}(p)$ denote the lightness, red–green, and yellow–blue components, respectively, at pixel p. Three normalized appearance components were computed as equation 18 &19.


$$\begin{array}{c} \begin{array}{c} L_{n}(p)=\frac{L^{*}(p)}{\text{1}\text{0}\text{0}}, \end{array} \end{array}$$ (18)

$$\begin{array}{c} \begin{array}{c} A_{r}(p)=\text{clip}\left(\frac{a^{*}(p)}{\text{1}\text{2}\text{7}},\text{0},\text{1}\right),\;B_{y}(p)=\text{clip}\left(\frac{b^{*}(p)}{\text{1}\text{2}\text{7}},\text{0},\text{1}\right)\; \end{array} \end{array}$$ (19)

A soft granulation-related appearance response was then obtained as equation 20.


$$\begin{array}{c} \begin{array}{c} \tilde{G}(I)(p)=M_{\Omega }(p)\;A_{r}(p)\;\left[\text{1}-B_{y}(p)\right]\;L_{n}(p),\; \end{array} \end{array}$$ (20)

where $M_{\Omega }(p)$ is the binary ulcer-region mask. This formulation assigns greater response to relatively red/pink, non-dark, and non-yellow pixels, while suppressing darker necrosis-like and yellow slough-like appearance patterns. To reduce isolated pixel-level variation, the response map was smoothed using a 3×3 Gaussian kernel with σ=1.0, followed by min–max normalization within the ulcer region as equation 21:


$$\begin{array}{c} \begin{array}{c} G(I)(p)=\frac{{\tilde{G}}_{s}(p)-\underset{\Omega }{\text{min}}{\tilde{G}}_{s}}{\underset{\Omega }{\text{max}}{\tilde{G}}_{s}-\underset{\Omega }{\text{min}}{\tilde{G}}_{s}+\epsilon }\; \end{array} \end{array}$$ (21)

Pixels outside the ulcer mask were assigned a value of zero. The identical procedure and parameters were applied independently to both the generated image and its severity-aligned pseudo-reference image before calculation of the soft Dice-based GTFS. Consequently, G(I) represents a reproducible granulation-like chromatic appearance score rather than a clinically validated tissue-segmentation probability map.

For the Granulation Tissue Fidelity Score, the deterministic granulation-related appearance mapping procedure defined above was applied separately to each generated progression-like image and its corresponding severity-aligned pseudo-reference image. Let $G_{g}$ denote the normalized granulation-related appearance map of the generated image and $G_{r}$ denote the corresponding map of the pseudo-reference image. Both maps therefore lie within the range [0,1], with larger values indicating stronger granulation-like chromatic appearance within the ulcer-region domain.

The image-level GTFS was computed using a soft Dice similarity formulation, as given in equation 22:


$$\begin{array}{c} \begin{array}{c} GTFS_{i}=\frac{\text{2}\sum_{p\in \Omega }G({\hat{I}}_{i},p)G(I_{i}^{r},p)+\varepsilon }{\sum_{p\in \Omega }G({\hat{I}}_{i},p{)}^{\text{2}}+\sum_{p\in \Omega }G(I_{i}^{r},p{)}^{\text{2}}+\varepsilon } \end{array} \end{array}$$ (22)

where $GTFS_{i}$ denotes the granulation tissue fidelity score for the $i^{th}$ image pair, p represents the pixel location, Ω denotes the ulcer-region pixel domain, and ε is a small numerical stabilizer. The soft Dice formulation measures the spatial overlap and intensity-level consistency between granulation-related appearance patterns in the generated image and the severity-aligned pseudo-reference image.

The final Granulation Tissue Fidelity Score was obtained by averaging the image-level GTFS values across all N test samples, as shown in equation 23:


$$\begin{array}{c} \begin{array}{c} GTFS=\frac{\text{1}}{N}\sum_{i=\text{1}}^{N}GTFS_{i} \end{array} \end{array}$$ (23)

Higher GTFS values indicate stronger spatial consistency of granulation-related appearance patterns between generated progression-like images and severity-aligned pseudo-reference images. Since the granulation-related maps were derived algorithmically from the generated and severity-aligned pseudo-reference images rather than from clinician-verified tissue masks, GTFS should be interpreted as a task-specific exploratory measure of granulation-related appearance fidelity rather than as a standardized clinical tissue-validation metric.

Algorithmically, TCS was computed by extracting LBP maps from each generated and pseudo-reference image, converting these maps into normalized histograms, estimating chi-square histogram dissimilarity, transforming this distance into an image-level similarity score, and averaging the scores across the test set. GTFS was computed by deriving deterministic CIELAB-based granulation-related soft appearance maps within the ulcer region for both generated and pseudo-reference images, applying identical Gaussian smoothing and normalization, comparing the resulting maps using soft Dice similarity, and averaging the image-level scores across the test set. This formulation makes both metrics reproducible and clarifies their role as supplementary technical measures for texture preservation and granulation-related appearance fidelity.

Finally, the ability of the model to withstand perturbations was evaluated by adding synthetic noise and/or small occlusions to the input images and observing the stability and consistency of the progression-like output images. The ability to maintain coherent progression-like outputs in the presence of such perturbations further confirmed the robustness of the proposed synthesis framework. As a combination of these three evaluations, the proposed framework was technically effective in generating and visualizing severity-conditioned severity-progression-like DFU appearances from a single image, as shown in Fig 8. It should be noted that the evaluation protocol was developed to evaluate image fidelity, distributional similarity, texture preservation and preliminary visual plausibility. These measures are not clinical efficacy, diagnostic accuracy, treatment benefit or real-world decision-support performance measures. Qualitative assessment has not been formally examined by an expert clinician, and should only be used as a visual plausibility inspection and not as full clinical validation. The proposed framework would need to be assessed in a larger multi-centre clinician evaluation, patient longitudinal follow-up and prospective clinical outcome analysis before it would be considered clinically deployable.

**Fig 8:**
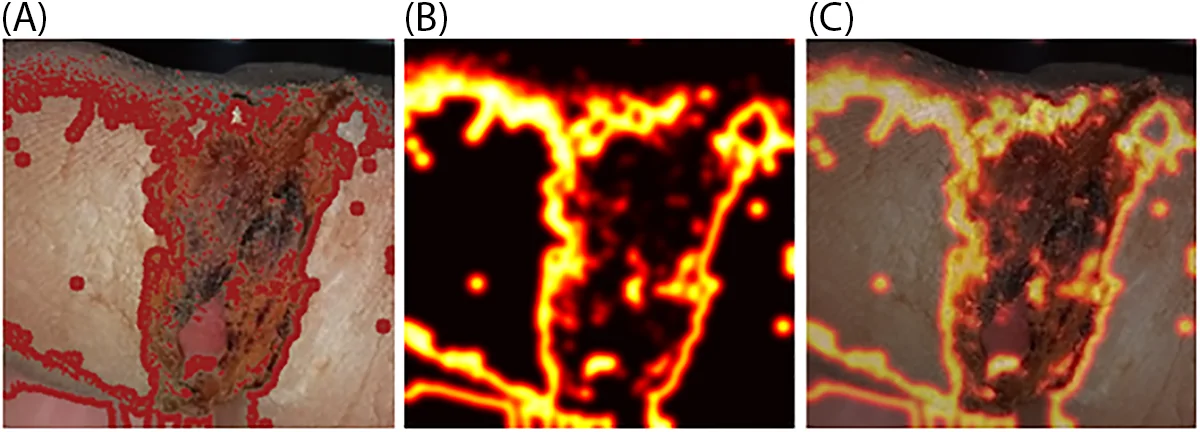
Visual comparison: progression-like synthesis, differences, and overlays.

## 4. Algorithmic summary of the proposed framework

### 4.1 Implementation details

All model development and experimentation were conducted using Python 3.10 and the PyTorch deep learning framework (version 2.0), providing the flexibility and modularity required to implement the custom conditional diffusion architecture and advanced texture-encoding mechanisms.High-efficiency batch-level operations and reproducible geometric and intensity transformations were achieved by using pre-processing and augmentation pipelines implemented with OpenCV and Albumentations library. To ensure generalizable texture and shape representation, the VGG-19 network used for perceptual feature extraction and loss computations was initialized with pre-trained weights on ImageNet and only intermediate layer activations were used without fine-tuning. Local Binary Pattern (LBP) computations were done with scikit-image while feature normalization was done with custom PyTorch modules to ensure smooth integration with the training pipeline. The training and inference were carried out on an NVIDIA RTX 3090 GPU with 24GB of VRAM, ensuring that the high resolution of ulcer images and the large intermediate feature maps could be processed without limitation of memory. The model was trained for 300 epochs with a batch size of 8, with the Adam optimizer and default values for β1 = 0.9 and β2 = 0.999, and an initial learning rate of ${\text{1}\text{0}}^{-\text{4}}$. A cosine annealing learning rate scheduler was employed to gradually decrease the learning rate, facilitating smoother convergence towards the later training stages. Weight decay with a coefficient of ${\text{1}\text{0}}^{-\text{5}}\;$was applied to mitigate overfitting and promote generalization. In order to reduce the variance of the model weights during training and enhance the output quality, the model weights were stored using an exponential moving average (EMA) with a decay factor of 0.999 throughout the training process. To track back and retest if there was divergence or instability, model checkpoints were saved at each epoch. Visualizations of intermediate attention maps and texture were produced after each 10 epochs as qualitative measures of model interpretability and texture fidelity for the progression-like synthesis. All quantitative experiments were automatically repeated with custom Python scripts to ensure consistency and minimize human bias in repeated experiments, such as SSIM and FID evaluations.

#### 4.1.1 Reproducibility and implementation details.

All experiments were performed with the same implementation pipeline, promoting reproducibility. All the experimental variants were trained and evaluated using the same preprocessing, architecture configuration and optimization parameters. Data partitioning and model initialisation were done using a fixed random seed to minimise the variance in the results caused by the stochastic factors as shown in Table 1. A diffusion-based U-Net backbone with texture-aware conditioning and cross-attention mechanisms was adopted for the proposed framework. The evaluation protocol and the definition of metrics for all baseline models, ablation variants and external validation experiments were the same as described in Table 2. To facilitate the fair evaluation under the limited dataset setting, the same random seed, preprocessing pipeline, data split, architecture configuration and evaluation protocol were adopted for the proposed model, baseline models, ablation models and external validation experiments. This design allows for reduced experimental variability, and enables the reproduction of the comparison between the model configurations.

**Table 1:** Model architecture summary.

Component	Specification
**Backbone**	U-Net–based diffusion architecture
**Texture Encoding**	Local Binary Patterns + deep perceptual features
**Conditioning Strategy**	Cross-attention–guided texture conditioning
**Input Resolution**	256 × 256 RGB
**Output**	Progression-like synthetic DFU images
**Diffusion Timesteps**	1000
**Attention Scope**	Ulcer-focused spatial attention

**Table 2:** Model architecture summary.

Parameter	Value
**Optimizer**	Adam
**Learning Rate**	1 × 10^-4^
**Batch Size**	8
**Training Epochs**	300
**Loss Functions**	Diffusion loss + texture consistency constraints
**Random Seed**	Fixed (reported)
**Hardware**	Single GPU

### 4.2 Proposed algorithm

The proposed algorithm is summarized in Algorithm 1. The algorithm starts by extracting texture feature encodings from a single pre-processed image of the diabetic foot ulcer and then merges local binary patterns and deep perceptual representations to create a single conditioning map. This texture-aware map is used to inform the subsequent conditional diffusion process, an iterative denoising process that starts with an initialized latent variable and uses learned texture and morphological information to guide the denoising. The final result is a high-fidelity synthetic image that mimics a progression-like ulcer appearance, preserving the global structure and micro-textural details that are important for clinical interpretation, and aligned to the severity of the ulcer.

Algorithm: Texture-aware diffusion-based severity-conditioned ulcer synthesis

**Input:** Single preprocessed ulcer image I

**Output:** Severity-conditioned progression-like synthetic DFU image I_progressed

1: # --------------------------

2: # Phase 1: Texture Feature Encoding

3: # --------------------------

4: Compute Local Binary Pattern (LBP) map: LBP_map ← ComputeLBP(I)

5: Extract deep perceptual features from VGG-19: VGG_feats ← ExtractVGGFeatures(I)

6: Normalize LBP_map and VGG_feats: LBP_norm ← Normalize(LBP_map), VGG_norm ← Normalize(VGG_feats)

7: Concatenate features: F_T ← Concatenate(LBP_norm, VGG_norm)

8: Project concatenated features: F_T ← LinearProjection(F_T_)

9: # --------------------------

10: # Phase 2: Diffusion Process Initialization

11: # --------------------------

12: Initialize noisy latent x_T ← SampleFromGaussian(μ = 0, σ = 1)

13: # --------------------------

14: # Phase 3: Reverse Diffusion with Texture Conditioning

15: # --------------------------

16: for t = T down to 1 do

17: Compute noise estimate ε_θ ← NoisePredictor(x_t, t, F_T)

18: Compute mean μ_θ and variance Σ_θ ← ParametrizePosterior(x_t, t, F_T, ε_θ)

19: Sample intermediate latent x_{t-1} ← SampleFromGaussian(μ_θ, Σ_θ)

20: end for

21: # --------------------------

22: # Phase 4: Obtain Progressed Ulcer Image

23: # --------------------------

24: I_progressed ← x_0 # Final severity-conditioned progression-like synthetic image

25: return I_progressed

## 5. Experimental results

### 5.1 Quantitative image quality evaluation

Table 3 summarizes the final performance of the full proposed diffusion model on the primary held-out test set using the same evaluation protocol applied in the baseline comparison presented in Table 5 and the full-model configuration reported in the ablation analysis in Table 7. Accordingly, the values in Table 3 represent a summary of the same final evaluation rather than a separate subset or alternative aggregation procedure.

**Table 3:** Summary of final full-model performance on the primary held-out test set.

Metric	Mean ± Standard Deviation	Interpretation
SSIM ↑	0.851 ± 0.020	High structural fidelity relative to severity-aligned pseudo-references.
FID ↓	44.33 ± 5.02	Strong distributional similarity between generated and real DFU images.
TCS ↑	0.79 ± 0.04	Good preservation of local micro-textural patterns.
GTFS ↑	0.76 ± 0.05	Consistent granulation-related appearance fidelity.

The quantitative evaluation summarized in Table 3 demonstrates the final performance of the proposed texture-aware conditional diffusion framework on the primary held-out test set. The full model achieved an SSIM of 0.851 ± 0.020, indicating strong structural similarity between the generated progression-like synthetic images and the corresponding severity-aligned pseudo-reference images. The FID value of 44.33 ± 5.02 indicates favorable distributional similarity between the generated image set and the real DFU image distribution. In addition, the Texture Consistency Score of 0.79 ± 0.04 demonstrates preservation of local micro-textural characteristics, while the Granulation Tissue Fidelity Score of 0.76 ± 0.05 indicates consistent representation of granulation-related appearance patterns. These values correspond to the same final full-model evaluation reported in Table 5 and Table 7 and therefore do not represent an alternative aggregation or evaluation subset. Since longitudinal future-state images were unavailable, these metrics quantify synthetic image fidelity, structural consistency, texture preservation, and distributional realism rather than patient-specific longitudinal progression accuracy. All these quantitative results add to the technical strength and initial visual plausibility of the proposed framework. However, they do not independently validate clinical applicability, as image similarity metrics cannot measure treatment impact, diagnostic safety, or patient outcome improvement. It is important to clarify that no formal clinician-led expert review was conducted in the present study. Therefore, no Likert-scale scores, Cohen’s kappa values, or expert-agreement statistics are reported in the experimental results. The qualitative results presented in this section are based on visual plausibility inspection, qualitative visual comparisons, attention overlays, heatmaps, and cross-dataset visual inspection. These results are intended to support technical interpretation of synthetic image fidelity, texture preservation, distributional realism, and preliminary visual plausibility, rather than clinician-validated clinical realism or clinical decision-support effectiveness.

### 5.2 Texture consistency and granulation fidelity assessment

The evaluation presented in Table 4 assessed microstructural texture preservation and granulation-related appearance fidelity using two task-specific supplementary metrics: the Texture Consistency Score (TCS) and the Granulation Tissue Fidelity Score (GTFS). TCS was used to measure local texture-pattern similarity through LBP-based histogram analysis, as formally defined in Equations (15)–(17). GTFS was used to estimate the spatial consistency of granulation-related appearance patterns between generated images and algorithmically derived severity-aligned pseudo-reference appearance maps using the soft Dice-based formulation defined in Section 3.6. The obtained TCS value of 0.79 ± 0.04 suggests that the generated progression-like DFU images preserved fine-grained local texture patterns, including tissue roughness, keratinization-like appearance, and necrosis-like texture variation. Similarly, the GTFS value of 0.76 ± 0.05 indicates plausible spatial consistency of granulation-related appearance patterns in the generated images. These findings support preliminary visual plausibility and texture fidelity under controlled experimental conditions. The reliability of TCS and GTFS was assessed indirectly by examining their consistency with established image-quality metrics, ablation behaviour, and external dataset performance. In the ablation study, removal of texture conditioning and cross-attention reduced both TCS and GTFS along with SSIM and FID, indicating that these metrics followed the expected degradation pattern when important architectural components were removed. Similarly, external validation showed only modest reductions in TCS and GTFS under domain shift, suggesting preliminary cross-dataset stability. However, TCS and GTFS should be interpreted as exploratory task-specific indicators rather than standardized clinical evaluation metrics. These scores are not intended to replace established evaluation standards such as SSIM, PSNR, FID, clinician-led assessment, or prospective clinical validation. Further clinician-centred validation, larger clinician-annotated DFU datasets, inter-rater agreement analysis, and comparison with established wound assessment criteria are required before these metrics can be considered broadly reliable or generalizable.

**Table 4:** Texture consistency and granulation fidelity metric evaluations.

Metric	Mean ± Standard Deviation	Interpretation
**Texture Consistency Score (TCS) ↑**	0.79 ± 0.04	High preservation of fine-grained textural features.
**Granulation Tissue Fidelity Score (GTFS) ↑**	0.76 ± 0.05	Biologically plausible representation of granulation-related appearance variation.

### 5.3 Quantitative image-quality metrics

#### 5.3.1 SSIM, PSNR, and FID comparison.

The descriptive results in Table 5 show that the diffusion model achieved the most favorable observed values among the three evaluated models. The diffusion model obtained an SSIM of 0.851 ± 0.020, compared with 0.782 ± 0.031 for the GAN baseline and 0.738 ± 0.038 for the VAE baseline, indicating higher observed structural similarity to the severity-aligned pseudo-progression references. Similarly, the observed PSNR was 24.76 ± 1.12 dB for the diffusion model, compared with 21.98 ± 1.56 dB and 19.63 ± 1.83 dB for the GAN and VAE baselines, respectively. The diffusion model also produced the lowest observed FID of 44.33 ± 5.02, compared with 59.13 ± 6.59 for the GAN and 69.77 ± 7.44 for the VAE. These differences represent descriptive performance trends on the fixed held-out test set and should not be interpreted as statistically significant superiority because formal inferential testing was not performed. Accordingly, the results support a favorable descriptive comparison of synthetic image fidelity for the diffusion framework while remaining subject to the statistical limitations described in Section 5.10.

**Table 5:** Comparison of image quality metrics across model baselines.

Model	SSIM	PSNR (dB)	FID
**Diffusion**	0.851 ± 0.020	24.76 ± 1.12	44.33 ± 5.02
**GAN baseline**	0.782 ± 0.031	21.98 ± 1.56	59.13 ± 6.59
**VAE baseline**	0.738 ± 0.038	19.63 ± 1.83	69.77 ± 7.44

#### 5.3.2 SSIM distribution analysis.

The SSIM distributions in Fig 9 show higher observed similarity values for the diffusion model than for the GAN and VAE baselines. The diffusion-model distribution is shifted toward higher SSIM values and shows a comparatively narrower observed spread within the evaluated test set. I The data in Fig 9, when the diffusion outputs are isolated, shows a median SSIM of 0.852 with an interquartile range of 0.837–0.865. The small IQR values indicate that the diffusion model consistently generates images with high structural fidelity, while the larger IQR values for GAN and VAE suggest that they may have difficulty maintaining a consistent quality. These results collectively highlight the potential of the diffusion model to produce synthetic DFU images with structural features similar to and stable with regard to progression.

**Fig 9:**
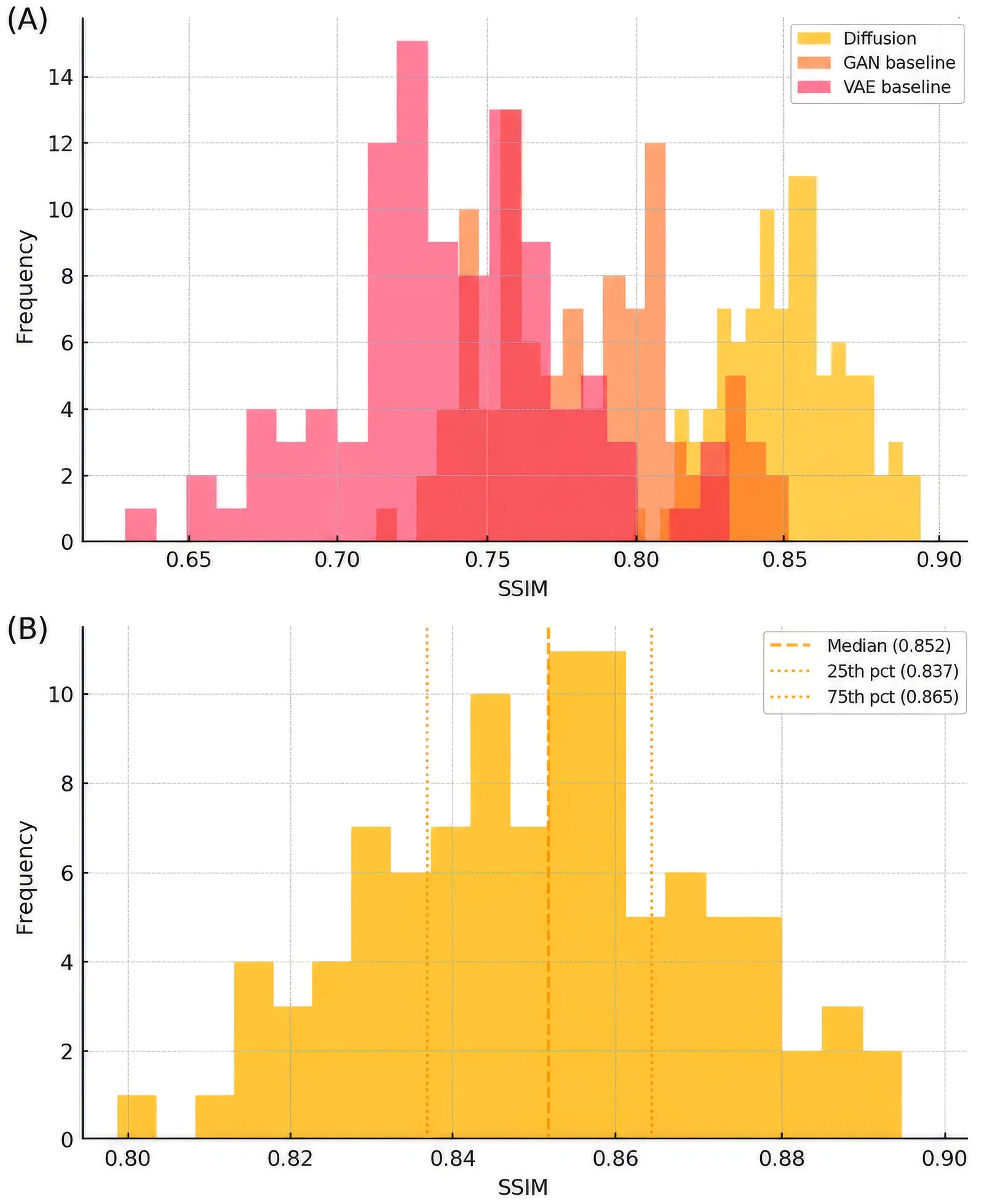
SSIM distributions comparing models and diffusion outputs.

#### 5.3.3 FID variation across diffusion timesteps.

Fig 10 reveals a clear U-shaped FID profile over the diffusion timesteps: at the very beginning (steps 1–2), high FID values (~120–90) indicate that the model’s outputs are overly smooth and lack the necessary textural detail; as the process advances into mid-range steps (4–6), FID reaches its minimum (~45), signifying the sweet spot where both coarse anatomical structures and fine ulcer textures are most faithfully reconstructed; beyond this window (steps 8–10), FID climbs again (~70–120), reflecting under-refinement of key features. This timestep-wise analysis reveals that sampling around timesteps 4–6 yields the most perceptually realistic images, suggesting that focusing training or early-stopping criteria within this phase can improve the perceptual realism and distributional consistency of generated progression-like DFU outputs.

**Fig 10:**
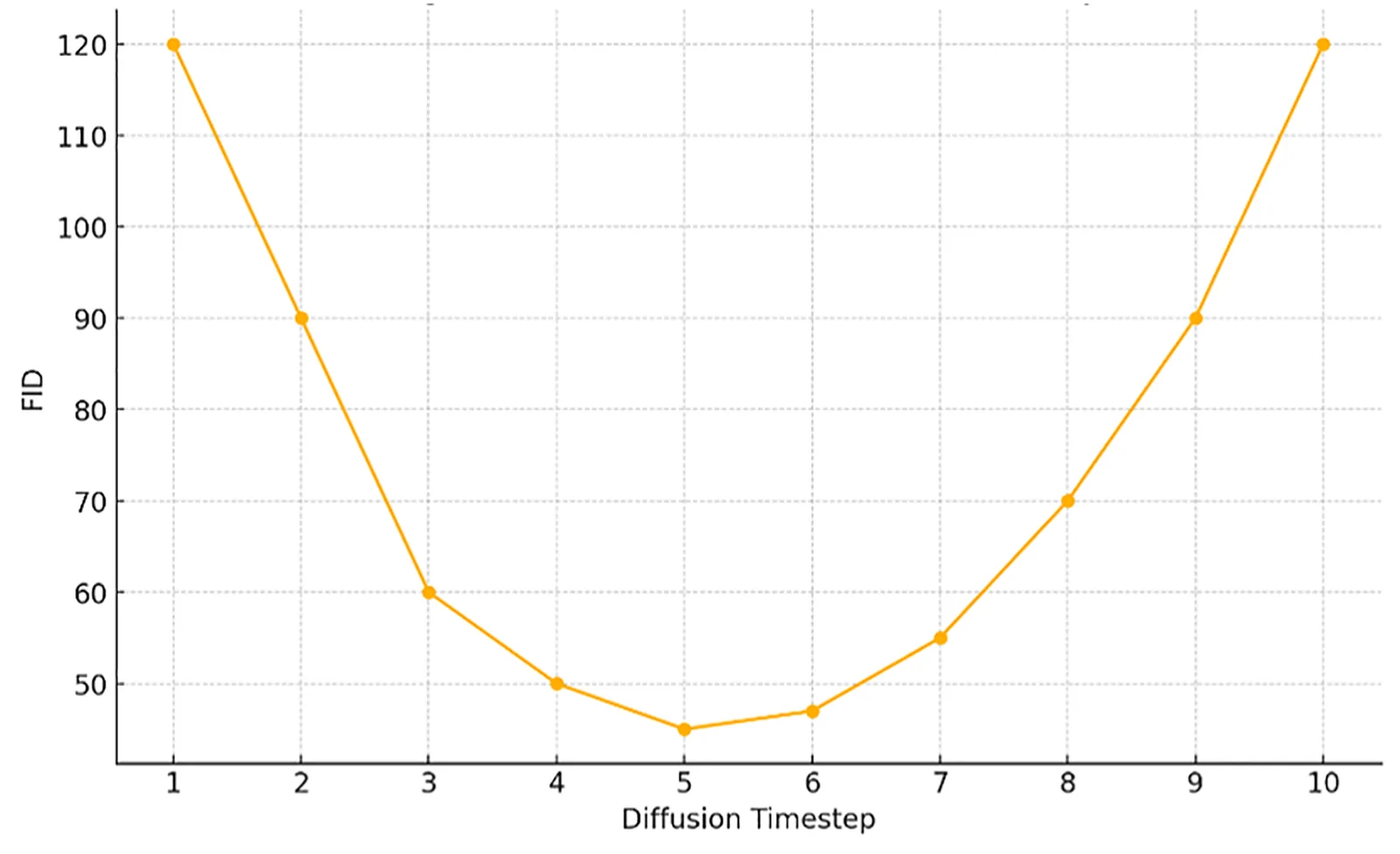
FID vs Diffusion timestep.

### 5.4 Qualitative visual comparisons

The qualitative visual comparisons in this subsection are presented as technical visual inspection outputs and should not be interpreted as formal clinician-rated assessment.

#### 5.4.1 SSIM overview.

The SSIM values over the three timesteps show a quantitative measure of the model’s performance across the pseudo-stages corresponding to the severity levels, as displayed in Table 6. The lowest SSIM value is 0.674 for timestep 1, suggesting that the synthetic reconstruction has the greatest difficulty at the very beginning, possibly because of the more complicated edge structures and texture gradient variations in the early wound formation. However, Timestep 2 has the best SSIM (0.804), indicating that the model performs better in terms of capturing the intermediate wound characteristics, which may be due to the fact that these stages contain more stable and less complex textures. Some loss of fidelity is seen in timestep 3 with a moderate SSIM of 0.726, especially in reproducing the deep central granulation and subtle surface variations typical of more advanced wounds. These findings, combined, indicate that the model has a good overall performance and can capture fine structural details and textural nuances well, but there is room for improvement in the early and advanced ulcer stages, where the performance was not as good. The values of SSIM calculated at each time step are used to indicate structural consistency with pseudo-progression references and are not the same as the accuracy of longitudinal progression.

**Table 6:** SSIM values for each timestep.

Timestep	SSIM
1	0.674
2	0.804
3	0.726

#### 5.4.2 Grid montage: Real vs. Synthetic vs. Difference.

The grid montage provides a step-by-step visualization of the proposed model’s ability to synthesize realistic progression-like DFU images, in an intuitive way. In all three timesteps, the synthetic outputs are able to accurately replicate the overall shape, wound size and general tissue structure of the real images. The absolute-difference heatmaps (right column), however, show areas of difference that are important for clinical interpretation. In particular, the model tends to damp down sharp edges of wound lesions and subtle variations in texture, resulting in higher error signals at the edges of lesions and at areas of slight tissue variation. For instance, the edges of the synthetic ulcer are not as sharp as those of the real image in Timestep 1. In Timestep 3, the central area of the wound is only very roughly approximated, while the rich textural details are somewhat lost. These spatial error patterns emphasize the need to improve the details of high-frequency reconstruction and preservation of edges. Overall, the montage suggests a good accuracy of the diffusion model in the global aspect and provides explicit knowledge about the points where the model can be improved on a micro aspect to make it more accurate, as shown in Fig 11.

**Fig 11:**
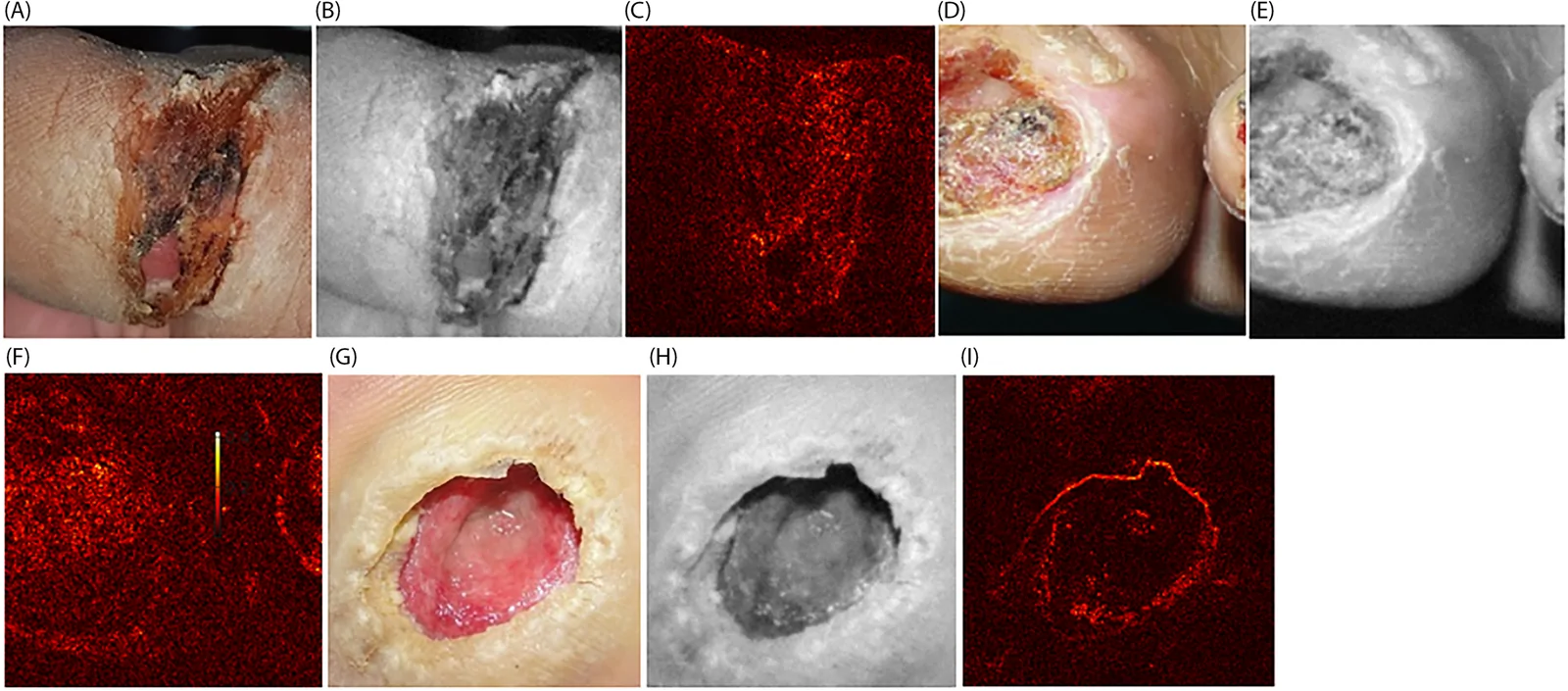
Stepwise montage: real vs synthetic vs error heatmap.

#### 5.4.3 Texture detail zoom-ins.

The generated progression-like DFU images are assessed visually in terms of the model’s ability to preserve micro-level texture details in the images through the 4 × magnified crop in Fig 12. The real image patch exhibits better definition of the ulcer margins, higher local contrast and better visibility of fine scale surface irregularities. Compared to the synthetic patch, the overall ulcer pattern and overall intensity gradient remain intact, but there is some smoothing of high frequency details, especially around the edge of the ulcer and areas of low tissue heterogeneity. The observation suggests that the proposed diffusion framework is more successful in maintaining the morphology of the ulcers at the macro level, rather than the very fine microstructural texture patterns. This over smoothing is a significant technical constraint as the transitions in texture at the edges could affect the visual interpretation of the appearance patterns that resemble granulation, slough and necrosis. Hence, the images created should not be regarded as complete substitutes of real high-resolution images of wounds, especially in the case of fine texture of the boundaries or subtle variation in the tissues. The zoom-in analysis is also presented to explicitly point out this limitation and to provide a guideline for future model refinement. Texture preserving techniques like frequency-aware loss functions, multi-scale patch-level supervision, boundary-aware conditioning, and edge-preserving post-refinement modules should be added in future iterations of the framework. The additions could enhance the retention of details that occur at high frequencies and preserve the overall structural consistency that is provided by the current model. Therefore, the proposed framework is supported by a balanced interpretation as presented in Fig 12, which shows that the framework is morphologically well preserved on a global scale, and yet there is a need for further refinement in terms of micro-texture preservation.

**Fig 12:**
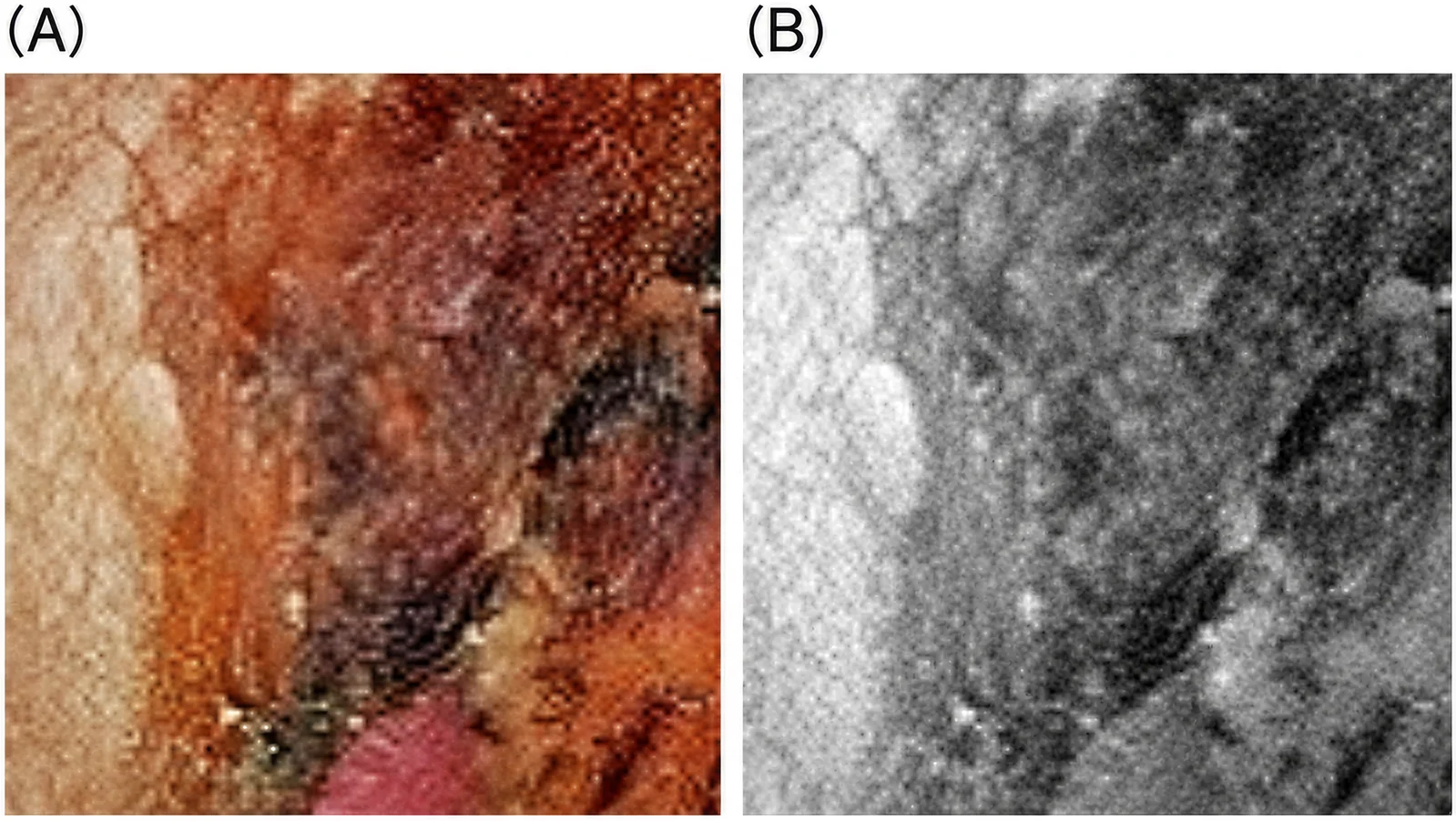
High-resolution zoom: real vs synthetic micro-texture details.

This smoothing effect is thus not considered as a small visual artefact, but as a major limitation in the present texture-aware framework with respect to texture preservation. In the current model, this is partially solved by explicitly conditioning the texture in the LBP stage, using deep perceptual features to guide the model, focusing on ulcer regions using cross-attention, and enforcing feature consistency loss. These components, however, better preserve the morphology at a global scale and the organization of the mid-level texture than very high-frequency microstructural detail, as seen in the zoom-in analysis. The finding indicates that texture awareness at conditioning level only is not enough for achieving the full recovery of texture at boundary level. Thus, future extensions should include explicit high-frequency texture constraints including frequency-domain reconstruction loss, Laplacian pyramid loss, boundary-aware texture supervision and local patch-discriminator refinement. The additions would directly address the smoothing effect seen in Fig 12 and enhance the texture preserving ability of the framework.

### 5.5 Attention overlay on progression-like synthetic output

The attention overlay in Fig 13 is essential to understand the spatial attention of the diffusion model in the synthetic DFU generation. This heatmap is based on the edge-based saliency signals and emphasizes the border of the ulcer as well as the central necrotic core with high intensity, and the healthy tissue is not given much attention. This spatial concentration implies that the model emphasizes the high-frequency textural and structural information closely associated with the morphology of ulcers, instead of the irrelevant background pattern. This focused attention gives initial evidence of interpretability; the highlighted regions match the regions of interest in the image, such as the margins and mid-body of the ulcer. Furthermore, the uniform localization of attention in various regions is helpful for the robustness and interpretability of the model, which facilitates transparent examination of the model behaviour. The visualization shows that when synthesizing DFU images that are more like progressions, the model puts emphasis on visually relevant ulcer regions, which is a confirmation that the model’s outputs are not only superficially realistic, but also supported by feature learning that is visually and texturally relevant to the ulcer region.

**Fig 13:**
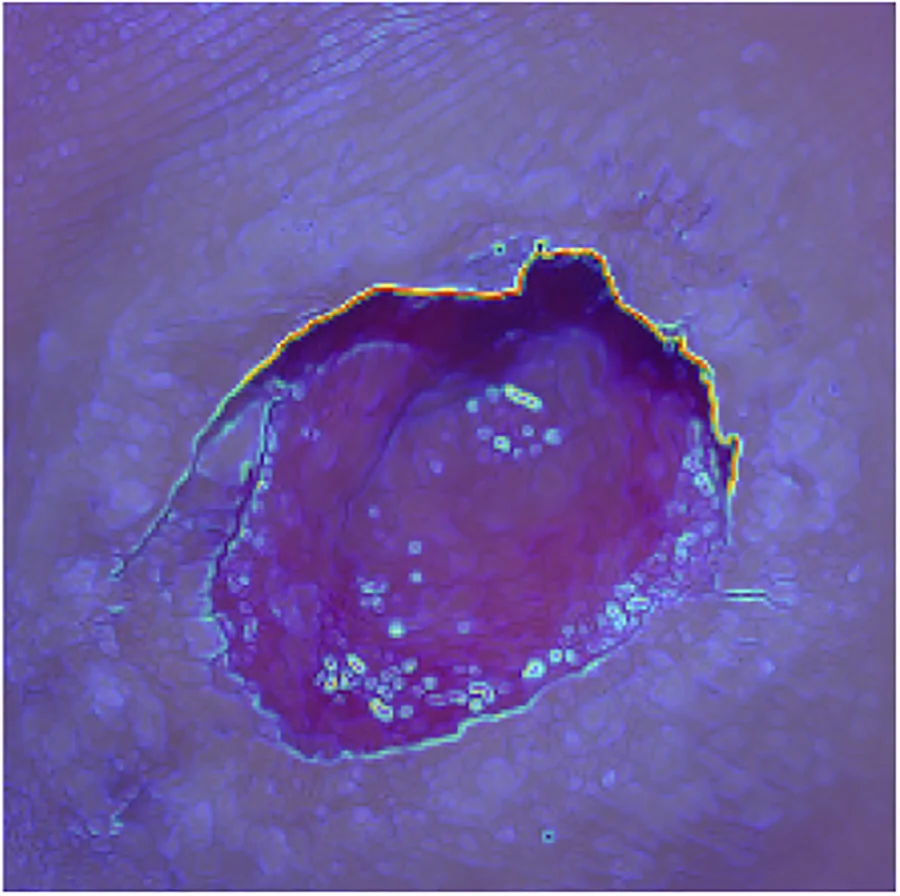
Attention heatmap highlighting clinically relevant ulcer regions in progression-like synthesis.

### 5.6 Severity-conditioned texture dynamics heatmap on baseline ulcer image

Fig 14 presents a severity-conditioned texture variation heatmap overlaid on the baseline ulcer image to show plausible regions of texture variation associated with the severity of the image. The aim of this visualization is to present a meaningful representation of the texture variation of the ulcer region, derived from the model, but keeping the anatomical context of the original image. Red-to-yellow areas in the heatmap represent relatively greater texture variation associated with severity, while blue-to-cyan areas represent relatively less texture variation associated with severity. This color-coded depiction allows visualization of the extent of localization of texture changes in the proposed framework during DFU synthesis of progression. In the case presented, the inner edges of the central ulcer bed and the surrounding inner edges of the ulcerated area are more texturally varied, indicating visually high change areas in the ulcer bed. The regions could be important for future clinically validated interpretation as central wound areas and margins of the ulcers may contain relevant visual cues that are important for tissue heterogeneity, surface irregularity and variation in appearance associated with the severity of the ulcer. The highlighted areas, however, do not necessarily represent clinical risk areas, areas of actual tissue degradation, or the actual areas of treatment. The heatmap is a model-based representation of appearance variation associated with severity and not clinically proven wound progression. The visualization is thus conceived as an exploratory research product to investigate model behavior and to gain insight into the spatial representation of the changes in texture for severity conditions. It could assist in a qualitative evaluation of whether the model captures the ulcer areas that are relevant to the image, rather than non-relevant background areas. Concurrently, care should be taken when interpreting this heatmap as it is not based on the longitudinal ground truth progression data or on prospective clinical outcome validation. Thus, the severity-conditioned texture dynamics heatmap should not be regarded as a clinically validated decision support tool. Do not use for debridement planning, patient counselling, treatment selection or for any type of clinical intervention without formal clinical validation. It is currently used as an exploratory visualizer, technical interpretability and analysis of severity-related texture variation in the proposed synthesis framework for progression. Future studies should confirm such heatmap outputs with longitudinal cohorts of DFUs, more clinician-centered assessments, and studies with outcomes to establish the clinical relevance of these visual patterns.

**Fig 14:**
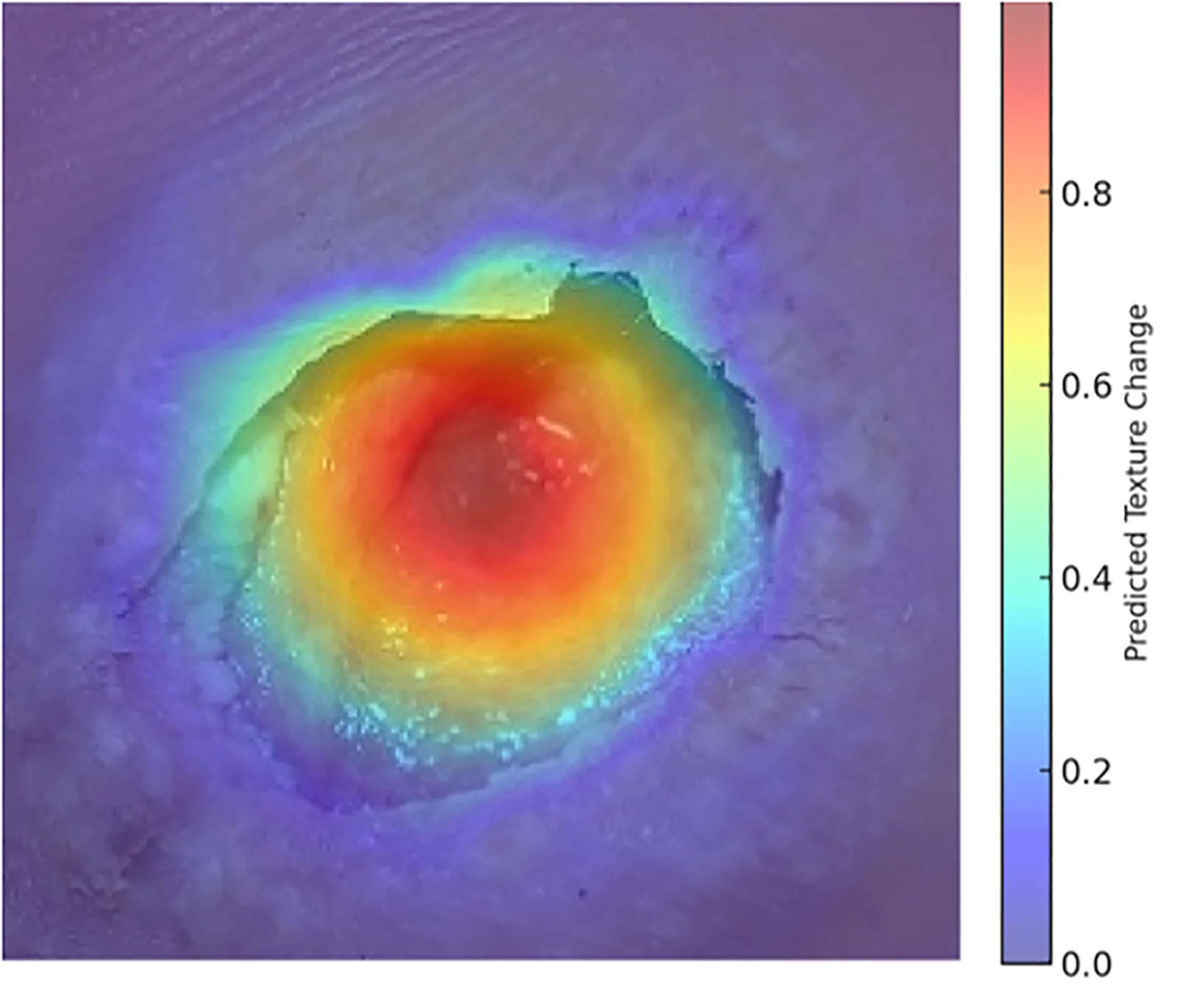
Severity-conditioned texture variation heatmap highlighting plausible high-change ulcer regions.

### 5.7 Multi-zonal overlay depicting plausible severity-conditioned tissue composition: Granulation, slough, and necrosis

Fig 15 shows a pixel-wise representation of the severity conditioned vision of the possible categories of appearance of the wound bed overlaid onto the base ulcer image. The green, yellow and black areas represent the granulation-like, slough-like and necrosis-like appearance patterns, respectively. These categories are intended to be used as model-generated visual appearance labels, and not as definitive clinical tissue diagnosis or direct treatment recommendations. The overlay combines these colour-coded appearance regions with the original anatomical context, allowing for exploratory examination of the tissue composition associated with severity, whilst maintaining the structural detail of the ulcer. The semi-transparency maintains the spatial relationships in the wound bed visible which helps in the interpretation of the output generated. Moreover, the region boundaries between various appearance zones allow a better visualization of the regional distribution of model identified tissue-like patterns.

**Fig 15:**
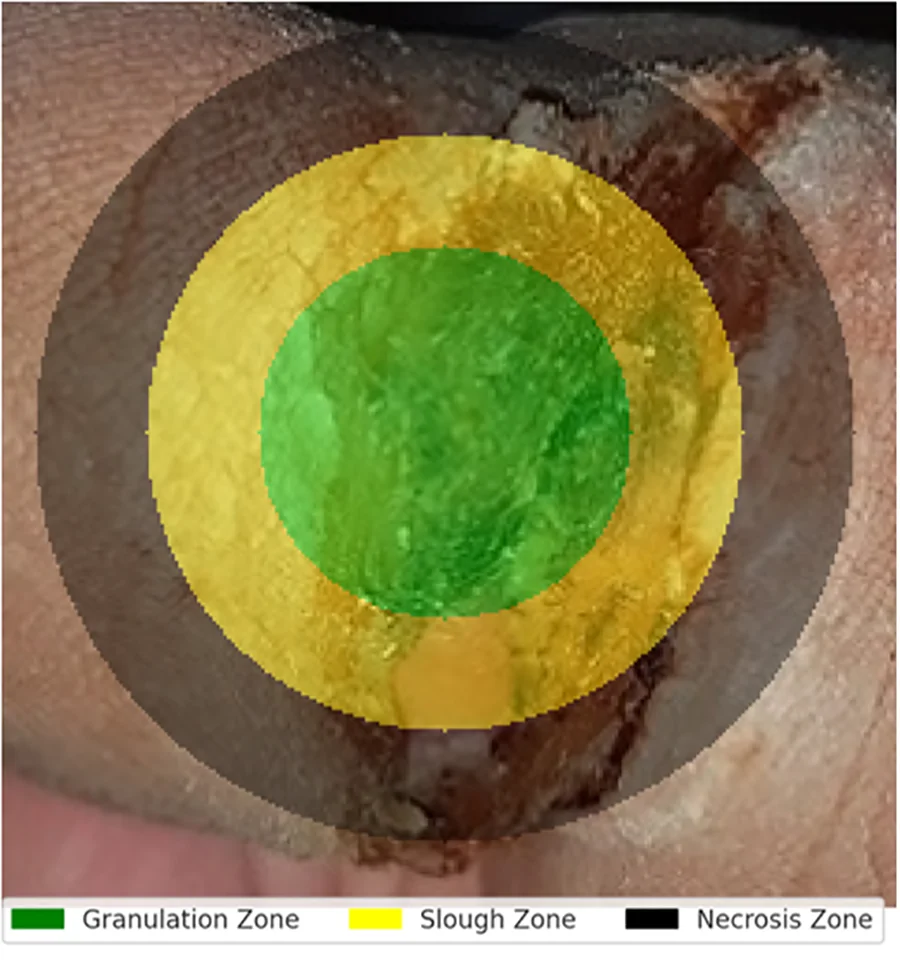
Multi-zonal overlay showing plausible severity-conditioned tissue composition zones.

This representation could be used as a visual cue for exploratory analysis of DFU synthesis for progression-like. However, its interpretation should remain cautious. The regions shown are not considered to be validated evidence of actual tissue status, biological progression or clinical risk. Therefore, the overlay should not be used as a replacement for direct clinical assessment, clinical expertise or accepted protocols for wound assessment. In the current phase, this visualization is to be considered as an interpretability tool for research purposes, for examining model behaviour and the appearance variation depending on the severity of the condition in the generated outputs. It is not intended for debridement planning, therapeutic decision-making, or any other form of clinical intervention. The possible clinical relevance of such multi-zonal overlays would necessitate further validation with larger clinician centered studies, longitudinal follow-up data sets and outcome-based evaluation. Hence, the use of this visualization is still restricted to the exploratory and methodological analysis, within the proposed progression-like synthesis framework.

### 5.8 Ablation study results on model components

The descriptive ablation results in Table 7 show that the full model produced the most favorable observed metric values among the evaluated configurations, with SSIM of 0.851 ± 0.020, FID of 44.33 ± 5.02, TCS of 0.79 ± 0.04, and GTFS of 0.76 ± 0.05. Removal of texture conditioning was associated with lower observed SSIM, TCS, and GTFS values and a higher FID, suggesting reduced preservation of local micro-textural characteristics and granulation-related appearance patterns. Removal of cross-attention also showed lower SSIM and higher FID values relative to the full model, indicating reduced structural similarity and distributional alignment. The configuration without either module produced the least favorable observed values across all four metrics. Taken together, these descriptive trends suggest that texture conditioning and cross-attention both contribute to the observed performance of the complete framework. However, because formal statistical significance testing was not performed, the observed differences should be interpreted as comparative trends on the fixed held-out test set rather than statistically established between-model effects.

**Table 7:** Ablation study highlighting contributions of key modules.

Model Variant	SSIM (↑)	FID (↓)	TCS (↑)	GTFS (↑)
**Full Proposed Model**	0.851 ± 0.020	44.33 ± 5.02	0.79 ± 0.04	0.76 ± 0.05
**Without Texture Conditioning**	0.802 ± 0.027	56.41 ± 6.18	0.69 ± 0.06	0.63 ± 0.07
**Without Cross-Attention**	0.815 ± 0.023	52.87 ± 5.91	0.72 ± 0.05	0.66 ± 0.06
**Without Both Modules**	0.778 ± 0.030	61.95 ± 7.02	0.65 ± 0.07	0.59 ± 0.08

### 5.9 External dataset validation and cross‑dataset generalization analysis

#### 5.9.1 External dataset description.

To further strengthen the robustness and generalisability of the proposed texture‑aware conditional diffusion framework, an additional external validation was conducted using an independent diabetic foot ulcer (DFU) image dataset obtained from Kaggle [31]. The external dataset contains 810 training images with labels, 200 validation images with labels and 200 test images, acquired in heterogeneous clinical conditions such as different light conditions, acquisition devices and ulcer appearance. This external validation was added in particular to address concerns regarding overfitting and limited data generalization. The primary dataset has only 493 DFU images, so the evaluation of the proposed framework using an independent dataset is more representative of the ability of the algorithm to learn the patterns of ulcer texture and morphology rather than the visual characteristics of the dataset.

More importantly, this external dataset was not used during the training, fine tuning or hyperparameter selection of the model, which allowed an unbiased evaluation of cross-dataset performance. The labels given in the dataset were only used for severity aligned grouping and evaluation and not as the temporal or longitudinal ground truth. This design is consistent with the pseudo progression paradigm used in the primary dataset and allows for meaningful external validation when there is a domain shift as demonstrated in Fig 16.

**Fig 16:**
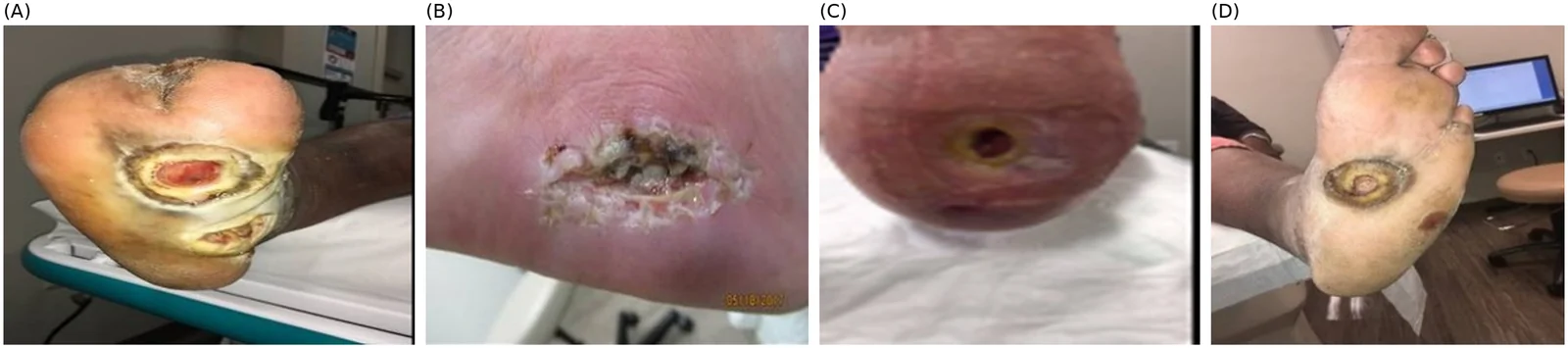
Sample images from external dataset.

#### 5.9.2 External validation protocol.

No retraining or adaptation was performed for the trained diffusion model on the external dataset and it was directly used for inference in a zero-shot setting. To guarantee methodological consistency in processing all images, the same pipeline of pre-processing was implemented, as described in Section 3.2, that consisted of resolution standardization, background suppression, texture enhancement and histogram normalization. A progression of synthetic images was created for each of the external input images with the same diffusion timestep setting used in the main experiments. The quantitative evaluation was done by SSIM, FID, Texture Consistency Score (TCS), and Granulation Tissue Fidelity Score (GTFS) and thus could be directly compared to the results reported in the primary dataset. In addition, qualitative visual inspections were performed to evaluate the plausibility of structural coherence, texture continuity and progression in cross dataset conditions.

#### 5.9.3 Quantitative results on external dataset.

The quantitative results in Table 8 show that the proposed framework is stable and competitive when tested on a new DFU dataset. Compared with the primary test set, a modest reduction in SSIM from 0.851 ± 0.020 to 0.801 ± 0.028 and in TCS from 0.79 ± 0.04 to 0.75 ± 0.05 was observed under external domain shift. The FID score is 38.7, which also suggests a high similarity between the distribution of generated images and the real image distribution, thereby demonstrating that the diffusion model can be applied to other data sources. The small drop in SSIM and TCS for the external dataset shows some sensitivity to domain shift, as one would expect in the absence of much training data. The proposed framework, however, is able to preserve reasonable values of FID, TCS and GTFS across the datasets, indicating that the framework is not purely memorisation of the primary dataset. The external dataset results also offer some initial insights into the generalizability of TCS and GTFS. Both scores dropped marginally from the primary data, but remained in a consistent range, suggesting that these scores can be used to measure consistency of appearance under domain shift in terms of texture and granulation. However, validation in larger multi-centre DFU datasets is needed for these metrics to be used as generalizable evaluation metrics.

**Table 8:** External dataset validation results.

Metric	Mean ± Standard Deviation	Interpretation
**SSIM ↑**	0.801 ± 0.028	Strong structural preservation under domain shift
**FID ↓**	38.7 ± 4.9	High perceptual realism across datasets
**Texture Consistency Score (TCS) ↑**	0.75 ± 0.05	Robust preservation of micro‑textural patterns
**Granulation Tissue Fidelity Score (GTFS) ↑**	0.72 ± 0.06	Biologically plausible granulation-related appearance representation

#### 5.9.4 Qualitative cross‑dataset analysis.

The external dataset was used to generate synthetic outputs that resemble progression; as shown in Fig 17, the boundaries of the ulcers, the central morphology of the wounds and the surrounding tissue context are preserved consistently across the progression-like synthetic outputs. Changes in progression appear to be spatially coherent, and there are gradual changes in texture rather than abrupt or unrealistic changes. Minor smoothing of higher frequency texture details, especially at the margins of ulcers are seen in some cases, as observed in the primary dataset, but the overall biological plausibility and clinical interpretability is preserved.

**Fig 17:**
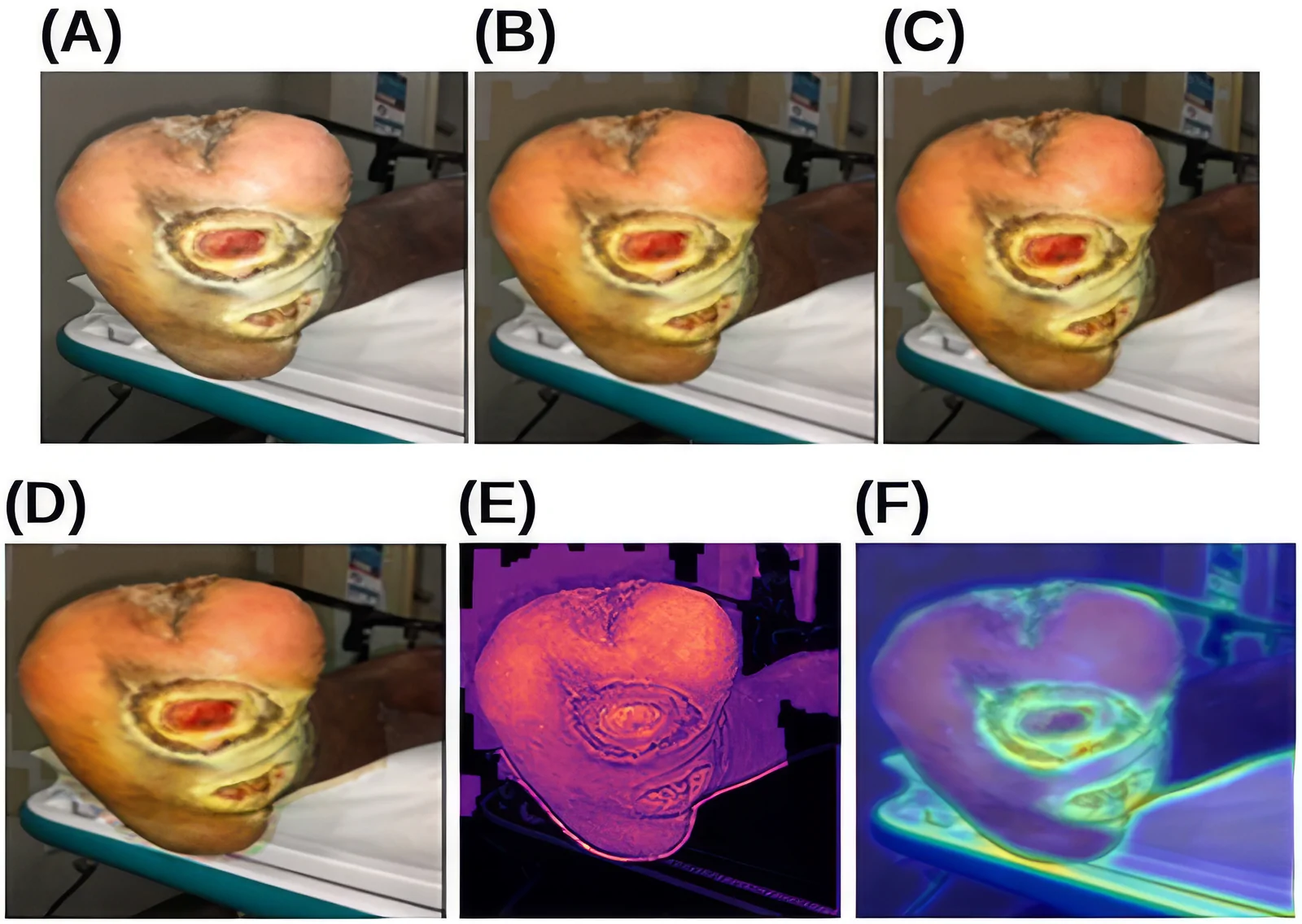
Cross-dataset progression-like DFU synthesis and risk visualization.

The results suggest that the proposed texture aware diffusion model is not simply memorizing the patterns of the dataset but instead learning transferable representations of ulcer texture and morphology that are effective on various clinical image distributions.

#### 5.9.5 Implications for longitudinal validation.

While the external validation results indicate the robustness of the proposed severity-conditioned synthesis framework across the different datasets, they may not be taken as evidence of true temporal prediction. The external dataset also does not have patient-level longitudinal follow-up, so the validation process also only supports the generalisability of progression-like visual synthesis under domain shift, but not chronological wound forecasting. While there is good cross dataset generalisability from external validation, this is not a substitute for true longitudinal validation. The primary and external datasets do not have patient level multi visit follow up images for direct evaluation of real temporal disease evolution. The pseudo progression framework used in this study is thus a weak form of progression, rather than a temporal predictor, such as visual transitions.

Longitudinal DFU cohorts in the future will need to be linked to clinically documented timepoints of follow up to directly assess temporal consistency, healing trajectories and clinical concordance, which can be validated. Nevertheless, the current external validation provides strong evidence that the proposed framework forms a reliable methodological foundation for future longitudinal modelling. Similarly, SSIM, FID, TCS, and GTFS values obtained during external validation indicate cross-dataset fidelity and texture consistency only. They do not confirm longitudinal progression accuracy because no patient-specific longitudinal ground truth was available in the external dataset.

### 5.10 Statistical considerations

Formal statistical significance testing between models was not performed due to the limited size of the held-out test set and the deterministic nature of the fixed data split. Instead, performance was reported using mean ± standard deviation across evaluation metrics, complemented by consistent performance trends observed across baseline comparisons, ablation studies, progression-stage analysis, and external dataset validation. This multi-faceted evaluation strategy was adopted to provide robust evidence of performance stability while avoiding potentially misleading significance estimates on small test samples. Additionally, because the pseudo-stages are derived from cross-sectional severity grouping rather than actual longitudinal follow-up, statistical comparisons are interpreted in terms of synthetic image fidelity and severity-conditioned consistency. They are not intended to validate real temporal prediction accuracy. True progression accuracy can only be assessed using longitudinal DFU cohorts with repeated imaging of the same patients over clinically documented follow-up intervals. Accordingly, terms implying formal statistical superiority or significance are avoided in the interpretation of the baseline and ablation comparisons. Differences in SSIM, PSNR, FID, TCS, and GTFS are reported as descriptive numerical trends on the fixed held-out test set and should not be interpreted as statistically significant between-model effects.

The absence of longitudinal future-state images also limits the interpretation of image-quality metrics. SSIM and PSNR assess similarity to severity-aligned pseudo-progression references, while FID assesses similarity to the overall real-image distribution. These metrics are useful for evaluating synthetic image quality but cannot determine whether a generated output matches the actual longitudinal future state of an ulcer. Therefore, the reported quantitative results should be interpreted as evidence of image fidelity and severity-conditioned consistency, not as validation of longitudinal progression accuracy.

## 6. Discussion

This study presents a texture-aware conditional diffusion framework for generating severity-conditioned progression-like synthetic images of diabetic foot ulcers (DFUs) from a single input image. The framework should be interpreted as a weakly supervised synthesis approach rather than a true temporal prediction model, because the available datasets consist of independent single-timepoint images without patient-specific longitudinal follow-up. By arranging cross-sectional images into severity-aligned pseudo-stages, the model learns population-level visual transitions associated with ulcer morphology and texture, rather than chronological wound dynamics within the same patient. The pseudo-progression assumption also has important physiological limitations. Diabetic foot ulcer evolution is not governed by visual severity alone, but is influenced by patient-specific biological and clinical factors, including vascular status, neuropathy, infection burden, glycaemic control, pressure redistribution, treatment response, wound care interventions, and systemic comorbidities. Since these factors were not available as longitudinal clinical variables in the dataset, the constructed pseudo-stages cannot represent the actual physiological trajectory of a specific ulcer. In addition, true wound progression may be non-linear, reversible, treatment-dependent, or interrupted by healing and deterioration events. Therefore, the generated outputs should be interpreted as severity-conditioned advanced severity-state visualizations rather than precise temporal forecasts of future ulcer evolution. For future longitudinal extension, the proposed framework can be adapted using a staged validation strategy. First, the current model may be retained as a cross-sectional severity-conditioned pretraining model for learning general ulcer texture and morphology representations. Second, the model should be fine-tuned on longitudinal DFU cohorts containing repeated images of the same ulcers across clinically documented follow-up intervals. In such a setting, pseudo-stage labels should be replaced or supplemented with actual follow-up targets, visit intervals, and available clinical covariates. Third, patient-level data splitting should be used to prevent leakage across training, validation, and testing subsets. Finally, generated follow-up images should be compared with real follow-up images using image-fidelity metrics, texture-preservation metrics, blinded clinician assessment, temporal consistency analysis, and clinical concordance with healing or deterioration outcomes. This future framework would allow direct assessment of whether severity-conditioned progression-like synthesis can be extended toward true patient-specific temporal modelling.

The best-performing variant achieves an SSIM of 0.851 ± 0.020, PSNR of 24.76 ± 1.12 dB, and FID of 44.33 ± 5.02, compared with the GAN baseline (SSIM: 0.782 ± 0.031, PSNR: 21.98 ± 1.56 dB, FID: 59.13 ± 6.59) and the VAE baseline (SSIM: 0.738 ± 0.038, PSNR: 19.63 ± 1.83 dB, FID: 69.77 ± 7.44). Additionally, the proposed Texture Consistency Score (TCS) and Granulation Tissue Fidelity Score (GTFS) further corroborate the biological realism of the generated images, with scores of 0.79 ± 0.04 and 0.76 ± 0.05 respectively, showing a good preservation of microstructural detail and plausible representation of granulation-related appearance variation. Ablation studies show the complementary benefits of texture conditioning and cross-attention. Explicit texture encoding for DFU synthesis is important as the removing texture conditioning reduced SSIM to 0.802 ± 0.027 and increased FID to 56.41 ± 6.18, with lower TCS 0.69 ± 0.06 and GTFS 0.63 ± 0.07. . Without cross-attention, SSIM is 0.815 ± 0.023, FID 52.87 ± 5.91, TCS 0.72 ± 0.05, and GTFS 0.66 ± 0.06, indicating that cross-attention helps to align conditioning signals and generated content in space. The variant with neither mechanism is most degraded (SSIM 0.778 ± 0.030, FID 61.95 ± 7.02, TCS 0.65 ± 0.07, GTFS 0.59 ± 0.08), suggesting that both mechanisms are important for good progression-like synthesis. Additionally, the progression-stage analysis shows that the model can capture the appearance variation from the beginning to the end of the progression, with SSIM scores of 0.674, 0.804, and 0.726 for the first, second, and third stage, respectively. Overall, the final full-model evaluation yielded an SSIM of 0.851 ± 0.020, FID of 44.33 ± 5.02, TCS of 0.79 ± 0.04, and GTFS of 0.76 ± 0.05, consistently reflecting structural fidelity, distributional similarity, and preservation of severity-related appearance characteristics across the reported analyses. However, without the ground truth longitudinal future images, SSIM, PSNR and FID cannot directly assess the accuracy of longitudinal progression. The three metrics: SSIM, PSNR, and FID, measure structural and pixel-level similarity to pseudo-progression references, and similarity between generated images and the current DFU image distribution, respectively. These metrics thus help to evaluate the quality of synthetic images and their realism in distribution, but do not prove that the synthetic image is the actual future state of a particular ulcer over time.

Additional support for potential clinical utility is provided by interpretability analyses. However, clinical implications of these findings should be interpreted cautiously. The main metrics used in the present validation are image similarity metrics, distributional realism metrics, texture-based scores, ablation analysis and qualitative visual plausibility inspection. These assessments are helpful in determining the technical quality of images and initial visual plausibility but they are not enough to determine the effectiveness of clinical decision support. The suggested framework has not yet been proven in potential clinical settings, has not been tested for impact on clinical decision making, and has not been correlated with patient-level outcomes like healing rate, infection prevention, or amputation reduction. Attention overlays and risk heat maps always focus on clinically relevant areas of ulcer (the central wound bed and peri-ulcer margins) but not on other background structures. The high-change regions found in the framework often coincide with the ulcer regions that are visually relevant such as necrosis-like, slough-like or granulation-like tissue appearance. The outputs should however be used as exploratory visualisations and should not be used as a guide for planning debridement, dressing selection or offloading without prospective clinical validation. These visual explanations will help future exploration of interpretable wound assessment workflows by providing intuitive, case-specific summaries of visual trajectories and regions that are likely to be associated with the severity of wounds, and should be monitored more carefully. An external validation was conducted on an independent dataset of DFU images obtained from Kaggle, that includes labelled images collected under varying clinical conditions in order to increase the robustness of the assessment. The proposed model was tested on this dataset in a zero-shot learning scenario without retraining or hyperparameter tuning and the same metrics as used for the main dataset. The external evaluation resulted in an SSIM of 0.801 ± 0.028, FID of 38.7 ± 4.9, TCS of 0.75 ± 0.05, and GTFS of 0.72 ± 0.06 which shows that it performs well under domain shift and only a small degradation compared to the primary test set. Qualitative examples showed that the morphology of the global foot and the shape of the ulcer remained unchanged, and that any changes that seemed to be progressing were limited to the ulcer bed and margins. These results indicate that the framework is not only learning appearance patterns of the datasets, but also learning transferable representations of ulcer texture and morphology that are able to generalize across different clinical image distributions. The results of the external validation of the proposed framework, presented in Section 5.9, also support the generalisability of the framework under different imaging conditions and ulcer presentations. The relatively small size of the primary data set is a factor to consider in interpreting the results. Typically, diffusion-based generative models work better with larger and more diverse datasets, so that using 493 images might make them more susceptible to overfitting to the imaging conditions of the dataset. This concern was partially mitigated by data augmentation, fixed validation protocols, ablation analysis and zero-shot external dataset validation. The external validation results show a structural and texture similarity under domain shift, but further multi-institutional validation is needed.

Several limitations should be acknowledged. First, both the primary and external datasets lack patient-level longitudinal image sequences with clinically documented follow-up time points. The pseudo-progression stages used in this work are therefore derived from severity-aligned groupings rather than true temporal observations. As a result, the framework should be interpreted as modelling progression-like visual transitions under weak supervision, rather than providing definitive temporal predictions of individual patient trajectories. Second, zoom-in analyses reveal some loss of very fine texture elements, particularly at ulcer boundaries, indicating that additional texture-preserving mechanisms such as frequency-aware generators, multi-scale refinement modules, or hybrid loss functions that emphasise high-frequency content may be beneficial. Third, although external validation supports cross-dataset generalisability, the datasets considered remain limited in size and geographic diversity, and performance may be influenced by differences in imaging protocols, lighting conditions, and patient characteristics. Although the proposed diffusion framework preserves global ulcer morphology and overall texture continuity, the zoom-in analysis in Fig 12 shows residual over-smoothing of high-frequency micro-textural details near wound boundaries. This limitation is particularly important for a texture-aware synthesis framework, because fine boundary-level texture transitions may influence the visual distinction between granulation-like, slough-like, and necrosis-like appearance patterns. The current architecture partially mitigates texture degradation through LBP-based texture conditioning, deep perceptual features, cross-attention-guided ulcer-region focusing, and feature consistency constraints. However, after iterative denoising these components do not fully recover the very fine high-frequency structures completely.

This is a limitation that needs to be addressed in future work specifically with refinement strategies that are frequency- and boundary-aware. The inclusion of a Focal Frequency Loss would help to penalize the missing high-frequency spectral components, and would encourage reconstruction of the fine surface irregularities, the sharpness of the wound edges and the subtle variations of the local tissue. Likewise, high-pass filtering the images before evaluating the discriminator would add a high-pass-filter guided adversarial loss, which would require the adversarial loss to be concerned with retaining micro-texture, as well as with global photorealism. Furthermore, Laplacian pyramid loss, multi-scale patch-level supervision, boundary-aware conditioning and an edge-preserving post-refinement module may be added to further enhance the preservation of high-frequency details. These additions are anticipated to help decrease smoothing without compromising the global structural fidelity that is already attained by the existing model. This study is mainly methodological and technically validates the methods with publicly available datasets. Structured usability studies with wound care specialists will be conducted in the future to assess the interpretability, clinical usefulness, and value of the decision support in real-life wound care management.

Future studies should focus on the inclusion of longitudinal DFU cohorts in which patients are followed across a number of visits with the same imaging techniques and comprehensive clinical annotations. These types of datasets would allow for direct assessment of temporal consistency, progression accuracy and clinical concordance, as well as calibration of progression like synthesis to actual healing or deterioration trajectories. Further increases in training and validation data to larger multi-institutional datasets would increase robustness and reduce potential biases from single-centre or single-source imagery. The explicit inclusion of clinical covariates (e.g., perfusion status, markers of infection, comorbidities) can also enable the modelling of the progression patterns in images and systemic risk factors together. The proposed texture-aware diffusion framework offers a methodological basis for the severity-conditioned progression-like visualization of DFU appearances. It should be used for technical synthesis and exploratory interpretation only, however, until larger longitudinal data sets, clinician-centred validation, and prospective outcome-based assessment are available. The proposed framework shows consistent improvement on various evaluation measures, but there are a few shortcomings in terms of statistical robustness that need to be recognized. Large annotated DFU datasets are not available, which makes patient-wise splitting and k-fold cross validation difficult, especially for the diffusion based generative models, which are computationally heavy. For this reason, in this study a fixed split strategy was used to guarantee the same and fair comparison of the models. In the future, more statistically sound validation methods will be investigated, such as cross validation and uncertainty aware evaluation, when more and more diverse DFU data sets are available. These will further build confidence in the generalisability of the models and their clinical applicability.

### 6.1 Comparative analysis of state-of-the-art methods

The results of the proposed texture-aware conditional diffusion framework are summarized in Table 9, which compares it with other methods. The models based on CNN or hybrid models are able to get good classification and segmentation accuracy, but they mainly work on static evaluation instead of progression-like severity-conditioned synthesis. The proposed method, on the contrary, enables the synthesis of a progression-like DFU from a single image, with high structural fidelity (SSIM: 0.851), excellent photorealism (FID: 44.33) and good preservation of biological texture (TCS: 0.79, GTFS: 0.76). Based on these results, the proposed method is expected to be a valuable methodological contribution for the synthesis of diabetic foot ulcers with progression and further validation is required to make generalization and clinical applicability statements.

**Table 9:** Comparison with state-of-the-art techniques.

Reference	Technique / Model	Dataset	Key Results / Accuracy
[21]	Deep learning (GoogLeNet, YOLO v3/v5, EfficientDet, U-Net, Mask R-CNN) for classification, detection, segmentation	Multiple DFU datasets (review)	Classification: up to 91.95% accuracy; Segmentation: U-Net, 94.96% accuracy; Mask R-CNN: precision 0.86
[4]	CNN + texture (mapped binary patterns)	Two public DFU datasets	AUC: 0.981 (healthy/unhealthy), 0.995 (ischaemia), 0.82 (infection)
[25]	Guided Conditional Diffusion Classifier (ConDiff)	DFU dataset [15]	81% accuracy, sensitivity 85.4%
[32]	Deep Learning (ResNet + Attention)	DFUC 2024	F1-score: 60.81%, AUC: 87.37%, Recall of 61.04%, and Precision of 61.56%
[33]	Thermogram-based SVM + DL	INAOE dataset	F1-score: 90% (concrete dropout features + SVM)
Proposed Method	Texture-aware conditional diffusion framework with hybrid texture encoding and cross-attention	Kaggle DFU RGB images (493 cases)	SSIM: 0.851 ± 0.020; PSNR: 24.76 ± 1.12 dB; FID: 44.33 ± 5.02; TCS: 0.79 ± 0.04; GTFS: 0.76 ± 0.05

### 6.2 Limitations and future validation requirements

A central limitation of the present study is the absence of true longitudinal diabetic foot ulcer image sequences. The datasets used in this work consist of independent single-timepoint images rather than repeated follow-up images of the same patients. Therefore, the pseudo-progression stages were constructed using cross-sectional severity-related visual cues, such as ulcer size, depth, granulation prevalence, and necrotic coverage, rather than actual temporal wound trajectories. As a result, the proposed framework does not learn genuine patient-specific temporal progression dynamics. The generated outputs should therefore be interpreted as severity-conditioned progression-like visualizations, not as deterministic predictions of an individual ulcer’s future clinical state.

A related limitation is that the pseudo-progression paradigm effectively represents severity-conditioned appearance interpolation across different patients rather than true temporal modelling within the same patient. This limitation arises because the ordinal pseudo-stages are derived from cross-sectional DFU images belonging to independent cases, not from repeated follow-up observations of the same ulcer. The pseudo-stage labels were not treated as clinician-validated annotations; no formal clinician-led annotation or adjudication protocol was used to establish longitudinal or diagnostic ground truth for these groupings. Therefore, the term progression-like is used throughout this study to indicate severity-conditioned synthetic visualization under limited longitudinal data availability, not actual future progression prediction. True patient-specific temporal progression prediction would require longitudinal DFU cohorts containing repeated images of the same ulcers across clinically documented follow-up intervals.

Another limitation is related to the clinical validation of the generated images. The present evaluation is primarily based on image-quality and visual-fidelity metrics, including SSIM, FID, TCS, and GTFS, along with qualitative visual plausibility inspection. Although these evaluations are useful for assessing structural similarity, distributional realism, texture preservation, and preliminary visual plausibility, they are not sufficient to establish real-world clinical decision-support effectiveness. These metrics cannot determine whether the generated images improve diagnostic safety, treatment selection, healing outcomes, infection prevention, or amputation-risk reduction.

A further limitation is that no formal clinician-led expert validation was conducted in the present study. The qualitative assessment was limited to visual plausibility inspection and did not involve blinded review by wound-care specialists, Likert-scale scoring, or inter-rater reliability analysis. Therefore, the findings should be interpreted as technical evidence of synthetic image fidelity and visual plausibility rather than clinician-validated clinical realism. Future studies should include blinded assessment by multiple wound-care specialists, structured rating criteria, inter-rater agreement analysis, and comparison with established wound assessment standards.

A further limitation concerns the interpretation of SSIM, PSNR, and FID in the absence of true longitudinal future-state images. These metrics are not suitable for assessing the longitudinal future state of a particular ulcer. However, SSIM and PSNR measure similarity to pseudo-progression images that are matched to severity, and FID measures distributional similarity to the current DFU image database. Thus, the reported metrics should be treated as measures of the image fidelity of the synthetic images and the realism of the datasets and not as measures of the accuracy of the longitudinal progressions.

The size and type of publicly available datasets are also limiting the study. While the number of images was limited by pre-processing, augmentation, regularization and external validation to address overfitting issues, 493 images are still a relatively small dataset to train a diffusion-based generative model. However, the model could be affected by the imaging characteristics of the dataset, such as lighting, acquisition conditions, placement of the wound and the way the ulcers are presented. Thus, the findings should be considered as preliminary evidence of technical feasibility and cross-dataset robustness, not as evidence of generalizability. Future studies should use and validate the framework on larger, multi-centre DFU datasets that include a wider variety of imaging devices, patient populations, skin tones, wound locations, and clinical severity.

Patient-level identifiers were not available; therefore, patient-wise data splitting could not be performed. This may limit the ability to fully assess generalization across independent patients. In addition, the datasets may not capture the full diversity of diabetic foot ulcer presentations, imaging devices, lighting conditions, skin tones, wound locations, infection states, vascular status, and clinical treatment histories. Although external dataset validation was included to evaluate cross-dataset robustness, such validation does not replace longitudinal or prospective clinical validation.

A further technical limitation is the partial loss of fine micro-textural details observed in some generated images, particularly near ulcer boundaries. Since edge-level granulation, slough, and necrotic transitions may be clinically important, future versions of the framework should incorporate texture-preserving strategies such as frequency-aware loss functions, multi-scale refinement modules, boundary-aware conditioning, and patch-level supervision to improve high-frequency detail preservation.

A further limitation concerns the proposed Texture Consistency Score and Granulation Tissue Fidelity Score. Although these metrics provide task-specific information about micro-texture preservation and granulation-related appearance fidelity, they are newly introduced and should be interpreted as supplementary exploratory measures rather than established evaluation standards. Their reliability was supported only indirectly through consistency with ablation trends and external dataset performance. Future studies should validate these metrics against larger clinician-annotated datasets, multiple wound-care specialists, inter-rater agreement analysis, and established wound assessment criteria before they can be considered broadly reliable or generalizable.

Overall, the proposed framework should be considered a methodological and exploratory visualization approach at this stage. It provides a foundation for severity-conditioned progression-like diabetic foot ulcer synthesis, but it should not be regarded as a validated clinical decision-support system. Future studies should validate the framework using longitudinal DFU cohorts with repeated patient-specific images, larger multi-institutional datasets, blinded clinician studies, prospective workflow testing, and outcome-based clinical evaluation before clinical applicability or deployment can be claimed.

## 7. Conclusion and future scope

This study proposes a texture-aware conditional diffusion model for synthesizing severity-conditioned, progression-like diabetic foot ulcer images from a single RGB input image. The proposed approach combines hybrid texture encoding using local binary patterns and deep perceptual features with cross-attention enhanced diffusion modelling, thus addressing key limitations of existing progression-like ulcer synthesis techniques. The framework does not attempt any patient-specific temporal forecasting as there is no time-series data of follow-up images. The study, however, shows that weakly supervised pseudo-progression modelling can produce plausible visual transitions that are aligned with the severity of the disease and retain clinically relevant texture and morphology.

The quantitative evaluation demonstrated favorable performance across multiple image-quality measures. The proposed model achieved an SSIM of 0.851 ± 0.020, PSNR of 24.76 ± 1.12 dB, and FID of 44.33 ± 5.02, corresponding to higher observed SSIM and PSNR and lower FID values than the evaluated GAN and VAE baselines. The Texture Consistency Score (TCS: 0.79 ± 0.04) and Granulation Tissue Fidelity Score (GTFS: 0.76 ± 0.05) were also computed to demonstrate the model’s capacity to retain fine-grained texture and appearance patterns associated with granulation. The ablation studies further demonstrated the importance of the texture-conditioning and cross-attention modules, where their removal led to a decrease in structural fidelity, texture consistency, and distributional realism. The progression-stage analysis further confirmed the ability of the model to portray severity-aligned pseudo-stages with structural consistency at timesteps 1, 2 and 3 with SSIM values of 0.674, 0.804 and 0.726, respectively.

The quantitative findings should be interpreted cautiously. In the absence of true longitudinal future-state images, SSIM, PSNR and FID can be used to assess the fidelity of synthetic images, the consistency of their structure, and the realism of their distribution, but not the accuracy of longitudinal progression. Therefore, these metrics should not be interpreted as indicators of whether a generated image accurately represents the future appearance of an individual patient’s ulcer. Longitudinal datasets will be needed in the future to determine if the images generated are progression-like images of real patients. Likewise, severity-conditioned risk heatmaps and attention overlays are not intended as clinical decision support outputs, but rather as exploratory visualizations.

Pseudo-progression stages were derived from predefined cross-sectional visual severity cues rather than longitudinal imaging data. Because these ordinal groupings are based on appearance characteristics rather than patient-specific temporal observations, they may not fully represent the biological complexity or temporal variability of diabetic foot ulcer progression. Moreover, small loss of micro-level textural details was noticed in zoomed areas, suggesting the requirement of texture optimized refinement modules or loss functions emphasizing high-frequency detail preservation. Another key constraint of diffusion-based DFU synthesis is the relatively small size of the primary data set. While data augmentation, regularization, fixed evaluation protocols, ablation analysis, and external dataset validation were employed to mitigate overfitting issues, the remaining 493 images are still relatively small for training a diffusion-based generative model. However, the model could be influenced by specific characteristics of the data, such as the lighting conditions, acquisition parameters, position of the wounds, and patterns of ulcer presentation.

The cross-dataset robustness was further validated with an external dataset, which helped alleviate the risk of memorizing the primary dataset. But this does not mean that this is a substitute for a more comprehensive multi-institutional assessment. Longitudinal image sequences of DFU taken at various clinical follow-up visits should be included in future studies to assess the temporal consistency, the patient-specific wound trajectory, and the accuracy of the longitudinal progression. In addition, larger multi-institutional DFU image collections are needed to minimize the risk of overfitting, increase the robustness over imaging conditions, and enhance the basis for assessing the generalization of diffusion-based DFU synthesis models.

The incorporation of multimodal data, such as thermal imaging, 3D surface scans, and clinical data (e.g., vascular status, infection markers, treatment history, and comorbidities) would also be valuable for future research. This information can improve the interpretability and severity-aware modelling of the image-based appearance patterns, as well as provide case-specific interpretability by associating image-based appearance patterns with clinically relevant patient factors. This framework would need to be translated into a real-time clinical decision support environment, and validated in a larger clinician-centred evaluation and outcome-based assessment before being deployed in the management of DFU. For future implementations, uncertainty estimation should also be integrated to provide information on the level of confidence of the outputs generated and to enable more confident interpretation of the severity-conditioned progression-like visualization in different clinical imaging settings and datasets. In general, the proposed framework offers a methodological base for severity-conditioned progression-like synthesis, risk-aware visualization and future longitudinal DFU modelling, highlighting the importance of thorough validation before any clinical claims can be made. Future studies should also involve formal validation of TCS and GTFS with larger clinician annotated DFU datasets and existing wound assessment standards to assess the reliability, reproducibility and generalizability of TCS and GTFS. While the pseudo-progression strategy gives an approximation of the severity-conditioned progression with limited availability of longitudinal data, true modelling of the progression over time will require longitudinal DFU datasets with repeated patient-specific images over clinically documented follow-up time.
