# Supplementary material for: Texture-aware diffusion modelling for severity-conditioned synthetic progression-like visualization of diabetic foot ulcers from single images
Source: PLoS One. 2026 Oct 12;21(10):e0359540. doi: 10.1371/journal.pone.0359540 (PMC13657312; doi:10.1371/journal.pone.0359540)
Supplement: Supplementary file 3 [file pone.0359540.s004.docx]

**RESPONSE SHEET**

| **REVIEWER 1** | |
| --- | --- |
| **COMMENT** | **RESPONSE** |
| The authors have substantially addressed the major concerns raised in the previous round, particularly regarding the reframing of "future progression prediction" to "severity-conditioned progression-like synthesis," the removal of unsupported expert-review claims, the addition of formal TCS/GTFS definitions, and the expanded discussion of texture over-smoothing limitations. The manuscript is now considerably more careful about the scope of its claims. However, a few issues remain before the manuscript can be accepted: | The reviewer’s careful reassessment and positive recognition of the substantial revisions are sincerely appreciated. The feedback has been invaluable in strengthening the manuscript’s methodological clarity, scientific rigor, and appropriate interpretation of the proposed severity-conditioned progression-like synthesis framework. |
| There is an unexplained discrepancy between the SSIM/FID values reported in Table 3 (SSIM 0.82 ± 0.03, FID 31.4) and Table 5 (SSIM 0.851 ± 0.020, FID 44.33 ± 5.02), both attributed to the same full proposed diffusion model. Please clarify the source of this difference (e.g., different aggregation method, different subset of results) or correct the inconsistency. | Thank you for identifying this inconsistency. The values in Section 5.1, Table 3 and its accompanying text were corrected to SSIM = 0.851 ± 0.020 and FID = 44.33 ± 5.02, consistent with Section 5.3.1 (Table 5) and Section 5.8 (Table 7). The corresponding comparison in Section 5.9.3 (Table 8 discussion) and the results reported in Section 6 (Discussion) were also updated to ensure complete numerical consistency throughout the manuscript. |
| The description of how pseudo-progression stage labels were assigned is inconsistent across sections (automated visual-cue-based grouping in Section 3.5 vs. "assigned by experts" in the Conclusion). Please clarify the exact labeling procedure and, if any manual/expert input was involved, provide details on the annotators and protocol. | Thank you for identifying this inconsistency. The pseudo-stage labeling procedure has been clarified in Section 3.5 (Phase 5: Training Strategy) as a predefined heuristic appearance-based severity grouping using explicit Stage 1–3 visual criteria, rather than longitudinal or clinically validated labels. The inconsistent “assigned by experts” statement was corrected in Section 7 (Conclusion and Future Scope), and the absence of a formal clinician-led annotation or adjudication protocol was clarified in Section 6.2 (Limitations and Future Validation Requirements). |
| The Granulation Tissue Fidelity Score (Eq. 18) relies on a "granulation-related appearance map" G(I) whose derivation is not specified. Please describe how this map is computed (e.g., color-space thresholding, a trained segmentation model) to allow reproducibility. | Thank you for highlighting this reproducibility issue. The derivation of the granulation-related appearance map $G\left( I \right)$has now been explicitly defined in Section 3.6 (Phase 6: Evaluation and Validation) using a deterministic CIELAB-based soft appearance mapping procedure, including ulcer-region masking, normalization, and Gaussian smoothing. Following the added derivation, the GTFS soft-Dice formulation is now presented as Eq. (22), and the corresponding description in Section 5.2 has been revised accordingly. |
| Given the small test set (n=74) and the explicit absence of formal significance testing (Section 5.10), the language describing the diffusion model's advantage over GAN/VAE baselines (e.g., "clear and consistent advantage") should be softened, or a suitable statistical test/bootstrap confidence interval should be added to support the comparison. | Thank you for highlighting this concern. Comparative language has been softened throughout the manuscript to avoid implying statistical superiority in the absence of formal significance testing, particularly in the Abstract, Section 5.3.1 (Table 5), Section 5.3.2, Section 5.8 (Table 7), Section 6 (Discussion), and Section 7 (Conclusion and Future Scope). Section 5.10 (Statistical Considerations) was also revised to clarify that the reported between-model differences represent descriptive numerical trends rather than statistically significant effects. |
| Minor language/formatting issues remain (duplicated figure captions/text blocks, some repetitive phrasing) that should be cleaned up at copyediting. | Thank you for highlighting these presentation issues. The manuscript has been carefully proofread to remove duplicated figure captions and repeated text blocks, while repetitive phrasing and minor language inconsistencies have been revised throughout for improved clarity, readability, and overall presentation. |
| **REVIEWER 3** | |
| Thank you very much for addressing all the comments from all the reviewers. The manuscript is now good. | Thank you for the positive feedback. |
