# Supplementary material for: Texture-aware diffusion modelling for severity-conditioned synthetic progression-like visualization of diabetic foot ulcers from single images
Source: PLoS One. 2026 Oct 12;21(10):e0359540. doi: 10.1371/journal.pone.0359540 (PMC13657312; doi:10.1371/journal.pone.0359540)
Supplement: Supplementary file 4 [file pone.0359540.s002.docx]

**RESPONSE SHEET**

| **REVIEWER 1** | |
| --- | --- |
| **Comment** | **Response** |
| The manuscript presents a novel texture-aware conditional diffusion model for synthetic progression prediction of diabetic foot ulcers (DFU) from single images. The work is technically solid, clinically relevant, and clearly demonstrates innovation through the integration of hybrid texture encoding and cross-attention within the diffusion framework. The introduction of biologically meaningful evaluation metrics (TCS and GTFS), along with ablation studies and expert validation, further strengthens the contribution. | Thank you for your positive feedback. |
| Dataset and Validation: The use of pseudo-progression stages is acknowledged as a limitation. Please expand on this in the discussion and, if possible, consider additional datasets or outline a clear pathway toward longitudinal validation. | The limitation associated with pseudo-progression has been explicitly expanded in the Discussion, clarifying that severity-aligned stages serve as a weakly supervised proxy rather than true temporal progression. To strengthen validation, an external labeled DFU dataset was added (Section 5.9) and evaluated in a zero-shot setting, demonstrating cross-dataset robustness. A clear roadmap toward future longitudinal validation using multi-visit clinical DFU cohorts has also been outlined. |
| Preservation of Fine Details: The generated images show some smoothing of micro-textural details, particularly at wound edges. Consider implementing or discussing refinement techniques to better retain clinically important structures. | The reviewer’s observation regarding smoothing of micro-textural details at wound edges is acknowledged. This limitation has been explicitly discussed in the revised Discussion section, where diffusion-induced boundary smoothing is clarified and several refinement strategies are outlined, including frequency-aware loss functions, multi-scale supervision, and edge-preserving refinement modules. |
| Generalizability: The robustness of the model across diverse clinical imaging conditions (lighting, acquisition protocols, patient demographics) should be further addressed, even if only in discussion. | This concern addressed through external dataset validation (Section 5.9), which evaluates robustness under heterogeneous imaging conditions, and further discussed in the revised Discussion. |
| Clinical Usability: Expanding expert validation with larger cohorts or usability testing would strengthen the claim of translational potential. | The reviewer’s suggestion regarding expanded expert validation and usability testing is appreciated. A dedicated statement has been added in the Discussion acknowledging that the present study focuses on methodological development and does not yet include large-scale clinical usability evaluation. Future work involving structured validation with wound-care specialists has been clearly outlined to strengthen translational applicability. |
| Presentation: The methodology section is dense and may benefit from a pipeline flowchart or summary tables to improve readability for readers from clinical backgrounds. | Thank you for the valuable suggestion. A detailed pipeline flowchart has been added to the Methodology section to visually summarise all stages of the proposed framework and improve clarity and accessibility for readers from clinical backgrounds. |
| Overall, this is a strong and promising study with high potential for clinical translation. With minor revisions, the manuscript will be suitable for publication in PLOSONE. | Thank you for the positive and encouraging assessment of the manuscript. The constructive feedback has been carefully addressed through targeted revisions, and the manuscript has been improved accordingly to enhance clarity, robustness, and clinical relevance. |
| **REVIEWER 2** | |
| The study uses only 493 images from a Kaggle dataset with minimal characterization. There is no information about patient demographics, ulcer grade distribution (Wagner/PEDIS classification), wound etiology, imaging protocols, or device specifications. This raises serious concerns about generalizability and potential biases.  Recommendation: Provide comprehensive dataset characterization, validate on at least one external dataset from a different source to demonstrate generalizability. | Thank you for raising this important concern. Additional details clarifying the characteristics and inherent limitations of the primary public dataset have been added in Section 3.1 (Dataset Description) and further discussed in Section 6 (Discussion), explicitly acknowledging the absence of patient-level demographics, ulcer grading, and imaging protocol metadata typical of publicly available datasets. To address generalizability, an independent external DFU dataset from a different source has been incorporated and evaluated in a zero-shot setting, as described in Section 5.9 (External Dataset Validation and Cross-Dataset Generalization Analysis), demonstrating stable quantitative performance and consistent qualitative behavior under domain shift. |
| The researchers created "pseudo-progression stages" by grouping different patients' ulcers based on severity rankings rather than tracking actual progression over time. This means the model is learning cross-sectional severity differences between different patients, not actual temporal progression dynamics of individual ulcers. The model cannot genuinely predict how a specific ulcer will evolve because it has never seen actual progression sequences.  Recommendation: Add a dedicated "Limitations" subsection explicitly acknowledging this fundamental constraint and its implications for clinical applicability. | Thank you for highlighting this important conceptual limitation. A dedicated “Limitations and Clinical Implications of Pseudo-Progression Modelling” subsection has been added to the Discussion, explicitly acknowledging that the pseudo-progression stages are derived from cross-sectional severity groupings rather than true longitudinal ulcer trajectories. |
| The paper reports standard deviations but lacks essential statistical details: exact test set sizes, data splitting methodology, statistical significance testing between models, and reproducibility information. It's unclear whether performance differences are statistically significant or whether good results might be due to favorable data splits.  Recommendation: Clearly specify exact numbers for training set size, validation set size, test set size, describe splitting strategy, Provide complete reproducibility package, like: Model architecture specifications in a table format, Complete hyperparameter settings, and Consider k-fold cross-validation | Thank you for highlighting the importance of statistical transparency and reproducibility. The revised manuscript now explicitly reports the exact training, validation, and test set sizes and the fixed data-splitting strategy in Section 3.1 (Phase 1: Data Collection). A dedicated Section 5.10 (Statistical Considerations) has been added to clarify the absence of formal significance testing and to justify the use of mean ± standard deviation supported by consistent trends across baselines, ablations, and external validation. Reproducibility details, including model architecture and hyperparameter settings, are now fully documented in Section 4.1.1 (Reproducibility and Implementation Details) with Tables 1 and 2, and limitations regarding k-fold cross-validation are explicitly discussed in Section 6 (Discussion). |
| **JOURNAL REQUIREMENTS** | |
| Please ensure that your manuscript meets PLOS ONE's style requirements, including those for file naming. | Thank you for the reminder. The manuscript and all associated files have been carefully reviewed and updated to fully comply with PLOS ONE formatting and file-naming requirements prior to resubmission. |
| Please note that PLOS One has specific guidelines on code sharing for submissions in which author-generated code underpins the findings in the manuscript. In these cases, we expect all author-generated code to be made available without restrictions upon publication of the work. Please review our guidelinesat <https://journals.plos.org/plosone/s/materials-and-software-sharing#loc-sharing-code> and ensure that your code is shared in a way that follows best practice and facilitates reproducibility and reuse. | Thank you for bringing this to our attention. The manuscript has been reviewed in accordance with PLOS ONE’s code-sharing guidelines, and the availability of all author-generated code underlying the reported findings has been clearly addressed in the revised submission. The code will be made openly accessible without restrictions upon publication, in compliance with PLOS ONE best practices for reproducibility and reuse. |
| We note that the grant information you provided in the ‘Funding Information’ and ‘Financial Disclosure’ sections do not match. When you resubmit, please ensure that you provide the correct grant numbers for the awards you received for your study in the ‘Funding Information’ section. | Thank you for noting this discrepancy. The funding details have been carefully reviewed and corrected, and the grant numbers in the Funding Information section have now been updated to accurately match those reported in the Financial Disclosure section in the revised manuscript. |
| Thank you for stating the following financial disclosure:  “This work is supported as part of a research grant by the Office of Research and Sponsored Programs at Abu Dhabi University, United Arab Emirates Grant reference 19300636.” Please state what role the funders took in the study.  If the funders had no role, please state: "The funders had no role in study design, data collection and analysis, decision to publish, or preparation of the manuscript." If this statement is not correct you must amend it as needed. Please include this amended Role of Funder statement in your cover letter; we will change the online submission form on your behalf. | Financial disclosure and funder’s role is mentioned clearly in the article. |
| Please note that funding information should not appear in any section or other areas of your manuscript. We will only publish funding information present in the Funding Statement section of the online submission form. Please remove any funding-related text from the manuscript. | Thank you for the clarification. All funding-related text has been removed from the manuscript, and funding information is now provided exclusively in the Funding Statement section of the online submission form, in accordance with PLOS ONE guidelines. |
| Please note that your Data Availability Statement is currently missing the repository name and the DOI/accession number of each dataset OR a direct link to access each database. If your manuscript is accepted for publication, you will be asked to provide these details on a very short timeline. We therefore suggest that you provide this information now, though we will not hold up the peer review process if you are unable. | Thank you for the reminder. The Data Availability Statement has been updated to include the repository name and direct access links for the datasets used in this study, in accordance with PLOS ONE requirements. |
| We note that Figures 1, 2, 3, 4, and 11 in your submission contain copyrighted images. All PLOS content is published under the Creative Commons Attribution License (CC BY 4.0), which means that the manuscript, images, and Supporting Information files will be freely available online, and any third party is permitted to access, download, copy, distribute, and use these materials in any way, even commercially, with proper attribution. For more information, see our copyright guidelines: <http://journals.plos.org/plosone/s/licenses-and-copyright>.  We require you to either (1) present written permission from the copyright holder to publish these figures specifically under the CC BY 4.0 license, or (2) remove the figures from your submission:  1. You may seek permission from the original copyright holder of Figures 1, 2, 3, 4, and 11 to publish the content specifically under the CC BY 4.0 license.  We recommend that you contact the original copyright holder with the Content Permission Form (<http://journals.plos.org/plosone/s/file?id=7c09/content-permission-form.pdf>) and the following text:  “I request permission for the open-access journal PLOS ONE to publish XXX under the Creative Commons Attribution License (CCAL) CC BY 4.0 (<http://creativecommons.org/licenses/by/4.0/>). Please be aware that this license allows unrestricted use and distribution, even commercially, by third parties. Please reply and provide explicit written permission to publish XXX under a CC BY license and complete the attached form.”  Please upload the completed Content Permission Form or other proof of granted permissions as an "Other" file with your submission.   In the figure caption of the copyrighted figure, please include the following text: “Reprinted from [ref] under a CC BY license, with permission from [name of publisher], original copyright [original copyright year].”  2. If you are unable to obtain permission from the original copyright holder to publish these figures under the CC BY 4.0 license or if the copyright holder’s requirements are incompatible with the CC BY 4.0 license, please either i) remove the figure or ii) supply a replacement figure that complies with the CC BY 4.0 license. Please check copyright information on all replacement figures and update the figure caption with source information. If applicable, please specify in the figure caption text when a figure is similar but not identical to the original image and is therefore for illustrative purposes only. | Thank you for raising this important copyright clarification. Figures 1, 2, 3, 4, and 11 consist entirely of author-generated images created specifically for this study and do not reproduce or adapt any third-party copyrighted material. As such, these figures are original works of the authors and are fully compatible with publication under the Creative Commons Attribution License (CC BY 4.0). The figure captions have been reviewed to ensure clarity regarding their original authorship, and no additional permissions are required. |
| Please ensure that you refer to Figure 5 in your text as, if accepted, production will need this reference to link the reader to the figure. | Thank you for the reminder. The manuscript text has been reviewed and updated to explicitly reference Figure 5 at the appropriate location, ensuring proper linkage between the figure and the corresponding discussion in the text. |
| Please include captions for your Supporting Information files at the end of your manuscript, and update any in-text citations to match accordingly. Please see our Supporting Information guidelines for more information. | Thank you for the clarification. The manuscript does not include any Supporting Information files. All figures, tables, and methodological details are fully contained within the main manuscript, and therefore no Supporting Information captions or in-text citations are required. |
| If the reviewer comments include a recommendation to cite specific previously published works, please review and evaluate these publications to determine whether they are relevant and should be cited. There is no requirement to cite these works unless the editor has indicated otherwise. | No specific previously published works were recommended for citation by the reviewer; therefore, no changes were required or made with respect to references in the revised manuscript. |
