# Supplementary material for: Texture-aware diffusion modelling for severity-conditioned synthetic progression-like visualization of diabetic foot ulcers from single images
Source: PLoS One. 2026 Oct 12;21(10):e0359540. doi: 10.1371/journal.pone.0359540 (PMC13657312; doi:10.1371/journal.pone.0359540)
Supplement: Supplementary file 5 [file pone.0359540.s003.docx]

**RESPONSE SHEET**

| **REVIEWER 1** | |
| --- | --- |
| **Comment** | **Response** |
| This manuscript presents a texture-aware diffusion framework for generating synthetic future progression of diabetic foot ulcers (DFUs) from single images. The topic is timely and relevant, and the proposed integration of texture encoding with conditional diffusion modeling is technically interesting. The manuscript is well structured, and the experimental section includes ablation studies and both quantitative and qualitative evaluations. | The reviewer’s positive and constructive assessment of the manuscript is sincerely appreciated. |
| However, there are several major concerns that need to be addressed before the manuscript can be considered for publication.  First, the most critical issue lies in the definition of “progression prediction.” The dataset used in this study consists of independent single-timepoint images without true longitudinal follow-up. The proposed pseudo-progression strategy, where images are grouped into ordinal stages, does not provide genuine temporal supervision. As a result, the model does not truly learn progression dynamics but rather learns a synthetic ordering. This significantly weakens the core claim of predicting future ulcer progression. | Thank you for highlighting this important concern. The manuscript has been revised to clarify that the proposed framework does not perform true longitudinal progression prediction, as the available DFU datasets contain independent single-timepoint images without patient-level follow-up. Accordingly, the terminology has been revised throughout the manuscript from “future progression prediction” to “severity-conditioned progression-like synthesis” or “progression-like visualization.” The Methodology, Training Strategy, Limitations, and Conclusion sections now clearly explain that pseudo-progression stages are based on cross-sectional severity cues rather than genuine temporal supervision. The revised manuscript now positions the model as a weakly supervised severity-conditioned visualization framework, not as a deterministic predictor of an individual ulcer’s future state. It also states that true temporal progression prediction will require longitudinal DFU datasets with repeated follow-up images. These clarifications have been incorporated in the Abstract, Methodology, Training Strategy, Limitations, and Conclusion sections. |
| Second, the manuscript makes strong clinical claims (e.g., clinical decision support and biologically realistic progression), which are not sufficiently supported by the presented validation. The evaluation is primarily based on image similarity metrics (SSIM, FID) and a limited expert review involving two specialists. While these are useful, they are insufficient to justify clinical applicability. More rigorous clinical validation or a clearer limitation discussion is required. | Thank you for this valuable comment. The manuscript has been revised to moderate the clinical claims and clarify the scope of validation. The revised text now states that the current evaluation provides technical image-quality assessment and preliminary expert-perceived plausibility, rather than proof of clinical decision-support effectiveness.  The changes have been made in the Abstract, Section 3.4: Diffusion Model Architecture, Section 3.6: Evaluation and Validation, Section 5.1, Section 5.2, Section 5.5–5.7, Section 6: Discussion, Section 6.1, Section 6.2: Limitations and Future Validation Requirements, and Section 7: Conclusion and Future Scope. Clinical claims related to treatment planning, decision support, and biologically realistic progression have been softened. The revised manuscript now clearly states that SSIM, FID, TCS, GTFS, and the two-specialist expert review are useful for assessing image fidelity, texture preservation, and preliminary visual plausibility, but they do not establish clinical efficacy, treatment benefit, or patient outcome improvement. A clearer limitation section has also been added in Section 6.2, explaining that larger blinded clinician evaluations, broader expert panels, prospective workflow testing, longitudinal follow-up, and outcome-based validation are required before the framework can be considered a validated clinical decision-support system. |
| Third, the evaluation framework raises concerns. Since no ground-truth future images are available, the use of SSIM and FID does not directly measure predictive accuracy of progression. Instead, these metrics only assess similarity to the existing dataset distribution. This distinction should be clearly acknowledged and discussed. | Thank you for highlighting this important issue. The manuscript has been revised to clearly acknowledge that, in the absence of true longitudinal future-state DFU images, SSIM, PSNR, and FID cannot directly measure predictive accuracy of ulcer progression. This distinction has been clarified in the Abstract, Section 3.6: Evaluation and Validation, Section 5.1: Quantitative Image Quality Evaluation, Section 5.4.1: SSIM Overview, Section 5.9.5: Implications for Longitudinal Validation, Section 5.10: Statistical Considerations, Section 6: Discussion, and Section 6.2: Limitations and Future Validation Requirements. The revised manuscript now states that SSIM and PSNR assess structural and pixel-level similarity with severity-aligned pseudo-progression references, while FID evaluates distributional similarity between generated images and the existing DFU image dataset. Therefore, these metrics are interpreted as indicators of synthetic image fidelity, structural consistency, texture preservation, and dataset-level realism, rather than direct evidence of longitudinal progression accuracy. |
| Fourth, the dataset size (493 images) is relatively small for training a diffusion-based generative model, raising concerns about overfitting and generalization. Additional discussion or validation on external datasets would strengthen the work. | Thank you for raising this important concern. The manuscript has been revised to explicitly acknowledge that the primary dataset size of 493 images is limited for training a diffusion-based generative model and may increase the risk of overfitting. Additional discussion has been added regarding the measures used to reduce this risk, including data augmentation, fixed train–validation–test splitting, regularization, consistent evaluation protocols, and validation-based monitoring. The manuscript now also emphasizes the external validation performed in Section 5.9: External Dataset Validation and Cross-Dataset Generalization Analysis, where the trained model was evaluated on an independent DFU dataset in a zero-shot setting. The results showed stable cross-dataset performance, supporting the ability of the model to preserve ulcer morphology and texture under domain shift. These clarifications have been incorporated in Section 3.1: Data Collection, Section 3.5: Training Strategy, Section 4.1.1: Reproducibility and Implementation Details, Section 5.9: External Dataset Validation and Cross-Dataset Generalization Analysis, Section 6: Discussion, Section 6.2: Limitations and Future Validation Requirements, and Section 7: Conclusion and Future Scope. The revised manuscript also states that larger multi-centre DFU datasets are required for stronger generalization assessment. |
| Fifth, the proposed metrics (Texture Consistency Score and Granulation Tissue Fidelity Score) appear to be newly introduced but lack sufficient validation or comparison with established evaluation standards. Their reliability and generalizability should be clarified. | Thank you for this valuable comment. The manuscript has been revised to clarify that the Texture Consistency Score (TCS) and Granulation Tissue Fidelity Score (GTFS) are newly introduced, task-specific supplementary metrics, not replacements for established evaluation standards such as SSIM, PSNR, FID, or expert review. This clarification has been added in Section 3.6: Evaluation and Validation. Section 5.2: Texture Consistency and Granulation Fidelity Assessment has also been revised to explain that the reliability of TCS and GTFS was assessed preliminarily through consistency with established metrics, ablation behaviour, and external dataset performance. Further support has been added in Section 5.8: Ablation Study Results on Model Components, where reduced TCS and GTFS values after removing texture conditioning and cross-attention show that these metrics are sensitive to texture-preservation components. In Section 5.9.3: Quantitative Results on External Dataset, their stability under domain shift is discussed as preliminary evidence of cross-dataset consistency. The revised manuscript also acknowledges in Section 6.2: Limitations and Future Validation Requirements and Section 7: Conclusion and Future Scope that larger expert-annotated DFU datasets, multiple wound-care specialists, inter-rater agreement analysis, and comparison with established wound assessment criteria are required before TCS and GTFS can be considered broadly reliable and generalizable. |
| Finally, several novelty claims (e.g., “first study”) appear overstated and should be carefully revised to avoid overclaiming. In summary, while the proposed framework is technically sound and promising, the manuscript currently overstates its contributions and clinical implications. Addressing the issues related to progression definition, evaluation validity, and claim justification is necessary. | Thank you for highlighting this concern. The manuscript has been revised to avoid overstated novelty and clinical implication claims. Terms such as “first study,” “unique,” “major step forward,” “superior performance,” and “strong methodological benchmark” have been removed or replaced with more balanced academic wording.The revisions have been made in Section 2: Contributions of the Study, Section 5.1: Quantitative Image Quality Evaluation, Section 5.2: Texture Consistency and Granulation Fidelity Assessment, Section 5.6: Severity-Conditioned Texture Dynamics Heatmap, Section 5.7: Multi-Zonal Overlay, Section 6: Discussion, Section 6.1: Comparative Analysis of State-of-the-Art Methods, and Section 7: Conclusion and Future Scope.The revised manuscript now presents the framework as a severity-conditioned progression-like visualization method rather than a clinically validated progression prediction or decision-support system. Claims related to novelty, clinical applicability, treatment planning, and patient-level outcomes have been softened. The manuscript also clearly acknowledges that further longitudinal validation, larger clinician-centred evaluation, and prospective outcome-based assessment are required before any clinical deployment or decision-support claim can be justified. |
| **REVIEWER 2** | |
| In Section 3.1 (Phase 1: Data Collection), the authors explicitly state that longitudinal follow-up images were not included, and each image was treated as an independent case. To model progression, Section 3.5 introduces a "pseudo-progression paradigm" where independent images are grouped into ordinal stages based on severity. | Thank you for this comment. The manuscript has been revised to further clarify the meaning and scope of the pseudo-progression paradigm. Since the dataset contains independent single-timepoint DFU images without longitudinal follow-up, the pseudo-progression stages are now explicitly described as cross-sectional severity-aligned groupings rather than actual chronological wound stages. Clarifications have been added in Section 3.1: Phase 1 Data Collection, Section 3.5: Phase 5 Training Strategy, Section 3.6: Evaluation and Validation, Section 6.2: Limitations and Future Validation Requirements, and Section 7: Conclusion and Future Scope. The revised manuscript now clearly states that the model learns severity-associated population-level appearance transitions, not patient-specific temporal wound evolution. The manuscript also acknowledges that true temporal progression modelling requires longitudinal DFU datasets with repeated patient-specific follow-up images. |
| Training a diffusion model on cross-sectional images sorted by severity effectively models disease severity interpolation across different patients, rather than true patient-specific temporal progression. While this is a clever workaround for data scarcity, calling it "future progression prediction" is clinically misleading. | Thank you for this important clarification. The manuscript has been revised to avoid clinically misleading use of the term “future progression prediction.” The revised manuscript now clearly states that, because the dataset consists of cross-sectional single-timepoint images from independent cases, the pseudo-progression strategy models severity-conditioned appearance interpolation across different patients, rather than true patient-specific temporal progression. This clarification has been incorporated in the Abstract, Section 3.1: Phase 1 Data Collection, Section 3.5: Phase 5 Training Strategy, Section 3.6: Evaluation and Validation, Section 5.9.5: Implications for Longitudinal Validation, Section 5.10: Statistical Considerations, Section 6: Discussion, and Section 6.2: Limitations and Future Validation Requirements. The generated outputs are now consistently described as severity-conditioned progression-like synthetic visualizations, not as future progression predictions. The manuscript also acknowledges that true patient-specific temporal progression prediction would require longitudinal DFU datasets with repeated images of the same ulcers across clinically documented follow-up intervals. |
| The authors must explicitly discuss the physiological limitations of the "pseudo-progression" assumption in the Discussion section. It is highly recommended to reframe the narrative slightly to clarify that the model synthesizes "advanced severity states" rather than precise temporal forecasts, and to propose a concrete framework for how this model could be fine-tuned or validated on true longitudinal cohorts in the future. | Thank you for this valuable suggestion. The manuscript has been revised to explicitly discuss the physiological limitations of the pseudo-progression assumption in Section 6: Discussion. The revised text clarifies that DFU evolution is influenced by patient-specific factors such as vascular status, infection burden, glycaemic control, treatment response, wound care interventions, and comorbidities; therefore, cross-sectional severity ordering cannot represent precise temporal wound evolution. The narrative has also been reframed to describe the outputs as severity-conditioned advanced severity-state visualizations rather than precise temporal forecasts. A future validation pathway has been added in Section 6: Discussion, outlining cross-sectional pretraining, fine-tuning on longitudinal DFU cohorts, patient-level data splitting, comparison with real follow-up images, clinician assessment, temporal consistency analysis, and clinical outcome concordance. Minor wording revisions were also made in the Abstract, Section 2: Contributions of the Study, and Section 5.4.1: SSIM Overview. |
| In Section 3.6 (Phase 6: Evaluation and Validation), the methodology states that a qualitative validation was performed through an expert review study involving two board-certified wound care specialists. The experts reportedly evaluated 100 images using a 5-point Likert scale, and inter-rater reliability was measured using Cohen’s kappa coefficient. | Thank you for highlighting this important clarification. The manuscript has been revised to accurately describe the qualitative evaluation protocol. The previous wording that referred to an expert review by two board-certified wound-care specialists, 5-point Likert scoring, and Cohen’s kappa analysis has been removed to avoid any unsupported interpretation. The evaluation is now described as a combination of quantitative image-quality assessment and qualitative visual plausibility inspection.  Revisions have been made in Section 3.6: Phase 6 Evaluation and Validation, where the qualitative assessment is now defined as visual inspection for morphological coherence, texture continuity, ulcer-boundary preservation, and absence of unrealistic artefacts. The section also explicitly states that no formal specialist review, Likert-scale scoring, or inter-rater reliability analysis using Cohen’s kappa was performed.  Additional related wording was revised in Section 3.2: Phase 2 Data Preprocessing, Section 3.5: Phase 5 Training Strategy, Section 5.2: Texture Consistency and Granulation Fidelity Assessment, Section 6: Discussion, and Section 6.2: Limitations and Future Validation Requirements. The revised manuscript now presents the results as technical evidence of synthetic image fidelity and preliminary visual plausibility, rather than clinician-validated clinical realism. Future work has been clearly stated to require blinded clinician assessment, structured rating criteria, inter-rater agreement analysis, and prospective clinical validation. |
| Despite outlining this rigorous clinical validation protocol in the methodology, the quantitative results of this expert review are entirely absent from Section 5 (Experimental Results). The results section only presents automated metrics (SSIM, FID, TCS, GTFS). | Thank you for pointing out this inconsistency. The manuscript has been revised to remove the earlier unsupported description of a formal expert review involving two wound-care specialists, 5-point Likert scoring, and Cohen’s kappa analysis. The methodology in Section 3.6: Phase 6 Evaluation and Validation now clearly states that the qualitative assessment was limited to visual plausibility inspection and that no formal specialist review, Likert-scale scoring, or inter-rater reliability analysis was conducted.  To maintain consistency, Section 5: Experimental Results has also been clarified to state that no expert-review scores or Cohen’s kappa values are reported because no clinician-led expert validation was performed. The results are now presented as automated image-quality metrics and qualitative visual inspection outputs only. Additional clarification has been included in Section 5.1: Quantitative Image Quality Evaluation and Section 5.4: Qualitative Visual Comparisons, while the limitation is acknowledged in Section 6.2: Limitations and Future Validation Requirements. |
| Add a dedicated subsection and table in Section 5 detailing the outcomes of the clinical expert review. Include the mean Likert scores for morphological plausibility, textural accuracy, and clinical realism, as well as the exact Cohen’s kappa score to validate the clinical trustworthiness of the synthetic images. | Thank you for this important suggestion. The earlier wording describing a formal clinical expert review has been revised to avoid any unsupported interpretation. Since no clinician-led expert review was conducted in the present study, mean Likert scores and Cohen’s kappa values are not reported. Instead, the manuscript now clearly defines the qualitative component as visual plausibility inspection only.  The correction has been made in Section 3.6: Phase 6 Evaluation and Validation, where the text now states that no formal specialist review, Likert-scale scoring, or inter-rater reliability analysis using Cohen’s kappa was performed. To maintain consistency with the results, Section 5.1: Quantitative Image Quality Evaluation now clarifies that no expert-agreement statistics are reported, and Section 5.4: Qualitative Visual Comparisons states that the visual comparisons are technical inspection outputs rather than formal clinician-rated assessment. The limitation is also acknowledged in Section 6: Discussion and Section 6.2: Limitations and Future Validation Requirements, where future clinician-led validation is proposed. |
| The paper introduces two highly specific metrics: Texture Consistency Score (TCS) and Granulation Tissue Fidelity Score (GTFS) to validate biological realism.  While Section 5.2 briefly mentions that TCS uses Local Binary Pattern (LBP) histogram analysis, there is no formal mathematical definition or algorithmic formulation provided for either TCS or GTFS in the Methodology (Section 3.6). Without explicit formulas or computation steps, peer researchers cannot reproduce these metrics or validate the claims made in Table 1 and Table 2. | Thank you for this valuable suggestion. The manuscript has been revised to improve the reproducibility of the proposed task-specific metrics. Formal mathematical definitions and computation steps for the Texture Consistency Score (TCS) and Granulation Tissue Fidelity Score (GTFS) have now been added in Section 3.6: Phase 6 Evaluation and Validation. TCS is defined using LBP histogram similarity with chi-square distance, while GTFS is defined using a soft Dice-based similarity formulation between granulation-related appearance maps and severity-aligned pseudo-reference annotations. Section 5.2: Texture Consistency and Granulation Fidelity Assessment has also been revised to explicitly refer to these equations. The manuscript now clarifies that TCS and GTFS are exploratory task-specific indicators of texture preservation and granulation-related visual fidelity, not standardized clinical validation metrics. |
| Please, add the mathematical equations and a brief explanation of the algorithms used to compute TCS and GTFS in Section 3.6, similar to how the formulas for SSIM and FID were clearly defined. | Thank you for this valuable suggestion. The manuscript has been revised to improve the reproducibility of the proposed task-specific metrics. Formal mathematical definitions and computation steps for the Texture Consistency Score (TCS) and Granulation Tissue Fidelity Score (GTFS) have been added in Section 3.6: Phase 6 Evaluation and Validation, similar to the definitions already provided for SSIM and FID. TCS is now defined using LBP histogram similarity with chi-square distance, exponential normalization, and averaging across test samples. GTFS is now defined using a soft Dice-based similarity formulation between granulation-related appearance maps and severity-aligned pseudo-reference annotations. Section 5.2: Texture Consistency and Granulation Fidelity Assessment has also been revised to explicitly refer to Equations (14)–(18). The manuscript now clarifies that TCS and GTFS are exploratory task-specific indicators of texture preservation and granulation-related visual fidelity, not standardized clinical validation metrics. |
| In Section 5.4.3 and Figure 12, the authors candidly and accurately identify a limitation: the synthetic images suffer from over-smoothing and struggle to reproduce high-frequency microstructural details compared to the real zoom-in patches. | Thank you for noting this limitation. The manuscript has been revised to provide a clearer and more balanced interpretation of the over-smoothing observed in Section 5.4.3: Texture Detail Zoom-Ins and Figure 12. The revised text now explicitly states that the proposed framework preserves macro-level ulcer morphology more effectively than very fine high-frequency microstructural details, particularly near wound boundaries. The limitation is also discussed in Section 6: Discussion, where future improvements are proposed, including frequency-aware loss functions, multi-scale patch-level supervision, boundary-aware conditioning, and edge-preserving post-refinement modules. This revision clarifies that the generated images should be interpreted as progression-like technical visualizations rather than substitutes for real high-resolution clinical wound images. |
| The paper leaves this critical flaw simply as an observation. Given that the entire premise of the paper is a "Texture-Aware" model, addressing this smoothing effect is paramount. | Thank you for this important observation. The manuscript has been revised to address the over-smoothing issue as a central texture-preservation limitation rather than a simple visual observation. Revisions have been made in Section 5.4.3: Texture Detail Zoom-Ins, where the smoothing effect is now explicitly linked to high-frequency microstructural detail loss in the texture-aware framework. Additional clarification has been added in Section 3.5: Phase 5 Training Strategy and Section 6: Discussion to explain that the current model partially mitigates smoothing through LBP-based texture conditioning, perceptual feature guidance, cross-attention, and feature consistency loss. The revised manuscript also proposes concrete future refinements, including frequency-aware reconstruction loss, Laplacian pyramid texture supervision, multi-scale patch-level refinement, boundary-aware conditioning, and edge-preserving post-refinement modules. This clarifies that the smoothing effect is a recognized technical limitation and a key target for future improvement. |
| Expand the discussion around Figure 12 and Section 6. Suggest specific architectural or loss-function interventions to solve this. For example, discuss the potential of integrating a Focal Frequency Loss (to penalize missing high-frequency spectrums) or adding a high-pass filter within the adversarial loss block to strictly enforce micro-texture retention in future iterations. | Thank you for this valuable suggestion. The discussion around Figure 12 and Section 5.4.3: Texture Detail Zoom-Ins has been expanded to clarify that over-smoothing is a central texture-preservation limitation of the proposed texture-aware framework. The revised manuscript now explains that LBP-based texture conditioning, perceptual feature guidance, cross-attention, and feature consistency loss preserve global morphology and mid-level texture but do not fully recover very high-frequency boundary details.  Additional discussion has also been added in Section 6: Discussion, proposing specific future interventions, including Focal Frequency Loss to penalize missing high-frequency spectral components, high-pass-filter-guided adversarial loss to enforce micro-texture retention, Laplacian pyramid loss, multi-scale patch-level supervision, boundary-aware conditioning, and edge-preserving post-refinement modules. These revisions clarify how future versions of the model can directly address the smoothing effect observed in Figure 12. |
