# Supplementary material for: Texture-aware diffusion modelling for severity-conditioned synthetic progression-like visualization of diabetic foot ulcers from single images
Source: PLoS One. 2026 Oct 12;21(10):e0359540. doi: 10.1371/journal.pone.0359540 (PMC13657312; doi:10.1371/journal.pone.0359540)
Supplement: Peer review history

# Peer review: Decision Letter 0

**DOI:** 10.1371/journal.pone.0359540.r001

**Authors:** Brian Weaver

4 Jan 2026

Dear Dr. Ati,

The manuscript has been evaluated by two reviewers, and their comments are available below. The reviewers have raised a number of concerns with the methodology in your manuscript that need attention. Could you please revise the manuscript to carefully address the concerns raised?

Please submit your revised manuscript by Feb 18 2026 11:59PM. If you will need more time than this to complete your revisions, please reply to this message or contact the journal office at ?>plosone@plos.org. When you're ready to submit your revision, log on to https://www.editorialmanager.com/pone/ and select the 'Submissions Needing Revision' folder to locate your manuscript file.

- A letter that responds to each point raised by the academic editor and reviewer(s). You should upload this letter as a separate file labeled 'Response to Reviewers'.
- A marked-up copy of your manuscript that highlights changes made to the original version. You should upload this as a separate file labeled 'Revised Manuscript with Track Changes'.
- An unmarked version of your revised paper without tracked changes. You should upload this as a separate file labeled 'Manuscript'.

If you would like to make changes to your financial disclosure, please include your updated statement in your cover letter. Guidelines for resubmitting your figure files are available below the reviewer comments at the end of this letter.

If applicable, we recommend that you deposit your laboratory protocols in protocols.io to enhance the reproducibility of your results. Protocols.io assigns your protocol its own identifier (DOI) so that it can be cited independently in the future. For instructions see: https://journals.plos.org/plosone/s/submission-guidelines#loc-laboratory-protocols. Additionally, PLOS ONE offers an option for publishing peer-reviewed Lab Protocol articles, which describe protocols hosted on protocols.io. Read more information on sharing protocols at https://plos.org/protocols?utm_medium=editorial-email&utm_source=authorletters&utm_campaign=protocols.

We look forward to receiving your revised manuscript.

Kind regards,

Brian Patrick Weaver, Ph.D.

Staff Editor

PLOS One

**Journal Requirements:**

When submitting your revision, we need you to address these additional requirements.

1. Please ensure that your manuscript meets PLOS ONE's style requirements, including those for file naming. The PLOS ONE style templates can be found at

https://journals.plos.org/plosone/s/file?id=wjVg/PLOSOne_formatting_sample_main_body.pdf and

https://journals.plos.org/plosone/s/file?id=ba62/PLOSOne_formatting_sample_title_authors_affiliations.pdf

2. Please note that PLOS One has specific guidelines on code sharing for submissions in which author-generated code underpins the findings in the manuscript. In these cases, we expect all author-generated code to be made available without restrictions upon publication of the work. Please review our guidelines at https://journals.plos.org/plosone/s/materials-and-software-sharing#loc-sharing-code and ensure that your code is shared in a way that follows best practice and facilitates reproducibility and reuse.

3. We note that the grant information you provided in the ‘Funding Information’ and ‘Financial Disclosure’ sections do not match.

When you resubmit, please ensure that you provide the correct grant numbers for the awards you received for your study in the ‘Funding Information’ section.

4. Thank you for stating the following financial disclosure:

“This work is supported as part of a research grant by the Office of Research and Sponsored Programs at Abu Dhabi University, United Arab Emirates Grant reference 19300636.”

Please state what role the funders took in the study.  If the funders had no role, please state: "The funders had no role in study design, data collection and analysis, decision to publish, or preparation of the manuscript."

If this statement is not correct you must amend it as needed.

Please include this amended Role of Funder statement in your cover letter; we will change the online submission form on your behalf.

5. Please note that funding information should not appear in any section or other areas of your manuscript. We will only publish funding information present in the Funding Statement section of the online submission form. Please remove any funding-related text from the manuscript.

6. Please note that your Data Availability Statement is currently missing the repository name and the DOI/accession number of each dataset OR a direct link to access each database. If your manuscript is accepted for publication, you will be asked to provide these details on a very short timeline. We therefore suggest that you provide this information now, though we will not hold up the peer review process if you are unable.

7. We note that Figures 1, 2, 3, 4, and 11 in your submission contain copyrighted images. All PLOS content is published under the Creative Commons Attribution License (CC BY 4.0), which means that the manuscript, images, and Supporting Information files will be freely available online, and any third party is permitted to access, download, copy, distribute, and use these materials in any way, even commercially, with proper attribution. For more information, see our copyright guidelines: http://journals.plos.org/plosone/s/licenses-and-copyright.

We require you to either (1) present written permission from the copyright holder to publish these figures specifically under the CC BY 4.0 license, or (2) remove the figures from your submission:

1. You may seek permission from the original copyright holder of Figures 1, 2, 3, 4, and 11 to publish the content specifically under the CC BY 4.0 license.

We recommend that you contact the original copyright holder with the Content Permission Form (http://journals.plos.org/plosone/s/file?id=7c09/content-permission-form.pdf) and the following text:

“I request permission for the open-access journal PLOS ONE to publish XXX under the Creative Commons Attribution License (CCAL) CC BY 4.0 (http://creativecommons.org/licenses/by/4.0/). Please be aware that this license allows unrestricted use and distribution, even commercially, by third parties. Please reply and provide explicit written permission to publish XXX under a CC BY license and complete the attached form.”

Please upload the completed Content Permission Form or other proof of granted permissions as an "Other" file with your submission.

In the figure caption of the copyrighted figure, please include the following text: “Reprinted from [ref] under a CC BY license, with permission from [name of publisher], original copyright [original copyright year].”

2. If you are unable to obtain permission from the original copyright holder to publish these figures under the CC BY 4.0 license or if the copyright holder’s requirements are incompatible with the CC BY 4.0 license, please either i) remove the figure or ii) supply a replacement figure that complies with the CC BY 4.0 license. Please check copyright information on all replacement figures and update the figure caption with source information. If applicable, please specify in the figure caption text when a figure is similar but not identical to the original image and is therefore for illustrative purposes only.

8. Please ensure that you refer to Figure 5 in your text as, if accepted, production will need this reference to link the reader to the figure.

9. Please include captions for your Supporting Information files at the end of your manuscript, and update any in-text citations to match accordingly. Please see our Supporting Information guidelines for more information: http://journals.plos.org/plosone/s/supporting-information.

10. If the reviewer comments include a recommendation to cite specific previously published works, please review and evaluate these publications to determine whether they are relevant and should be cited. There is no requirement to cite these works unless the editor has indicated otherwise.

[Note: HTML markup is below. Please do not edit.]

Reviewers' comments:

Reviewer's Responses to Questions

**Comments to the Author**

1. Is the manuscript technically sound, and do the data support the conclusions?

Reviewer #1: Yes

Reviewer #2: Partly

**********

2. Has the statistical analysis been performed appropriately and rigorously?

Reviewer #1: Yes

Reviewer #2: Yes

**********

3. Have the authors made all data underlying the findings in their manuscript fully available?

The PLOS Data policy

Reviewer #1: Yes

Reviewer #2: No

**********

4. Is the manuscript presented in an intelligible fashion and written in standard English?

Reviewer #1: Yes

Reviewer #2: Yes

**********

Reviewer #1: The manuscript presents a novel texture-aware conditional diffusion model for synthetic progression prediction of diabetic foot ulcers (DFU) from single images. The work is technically solid, clinically relevant, and clearly demonstrates innovation through the integration of hybrid texture encoding and cross-attention within the diffusion framework. The introduction of biologically meaningful evaluation metrics (TCS and GTFS), along with ablation studies and expert validation, further strengthens the contribution.

That said, I recommend the following points for revision to further improve the clarity, reproducibility, and impact of the study:

1. Dataset and Validation: The use of pseudo-progression stages is acknowledged as a limitation. Please expand on this in the discussion and, if possible, consider additional datasets or outline a clear pathway toward longitudinal validation.

2. Preservation of Fine Details: The generated images show some smoothing of micro-textural details, particularly at wound edges. Consider implementing or discussing refinement techniques to better retain clinically important structures.

3. Generalizability: The robustness of the model across diverse clinical imaging conditions (lighting, acquisition protocols, patient demographics) should be further addressed, even if only in discussion.

4. Clinical Usability: Expanding expert validation with larger cohorts or usability testing would strengthen the claim of translational potential.

5. Presentation: The methodology section is dense and may benefit from a pipeline flowchart or summary tables to improve readability for readers from clinical backgrounds.

Overall, this is a strong and promising study with high potential for clinical translation. With minor revisions, the manuscript will be suitable for publication in PLOS ONE.

Reviewer #2: 1, Dataset

The study uses only 493 images from a Kaggle dataset with minimal characterization. There is no information about patient demographics, ulcer grade distribution (Wagner/PEDIS classification), wound etiology, imaging protocols, or device specifications. This raises serious concerns about generalizability and potential biases.

Recommendation: Provide comprehensive dataset characterization, Validate on at least one external dataset from a different source to demonstrate generalizability.

2, Methodology

The researchers created "pseudo-progression stages" by grouping different patients' ulcers based on severity rankings rather than tracking actual progression over time. This means the model is learning cross-sectional severity differences between different patients, not actual temporal progression dynamics of individual ulcers.

The model cannot genuinely predict how a specific ulcer will evolve because it has never seen actual progression sequences.

Recommendation: Add a dedicated "Limitations" subsection explicitly acknowledging this fundamental constraint and its implications for clinical applicability.

3, Data incomplete

The paper reports standard deviations but lacks essential statistical details: exact test set sizes, data splitting methodology, statistical significance testing between models, and reproducibility information. It's unclear whether performance differences are statistically significant or whether good results might be due to favorable data splits.

Recommendation: Clearly specify exact numbers for training set size, validation set size, test set size, Describe splitting strategy, Provide complete reproducibility package, like: Model architecture specifications in a table format, Complete hyperparameter settings, and Consider k-fold cross-validation

**********

what does this mean?). If published, this will include your full peer review and any attached files.

If you choose “no”, your identity will remain anonymous but your review may still be made public.

**Do you want your identity to be public for this peer review?** For information about this choice, including consent withdrawal, please see our Privacy Policy

Reviewer #1: No

Reviewer #2: No

**********

[NOTE: If reviewer comments were submitted as an attachment file, they will be attached to this email and accessible via the submission site. Please log into your account, locate the manuscript record, and check for the action link "View Attachments". If this link does not appear, there are no attachment files.]

To ensure your figures meet our technical requirements, please review our figure guidelines: https://journals.plos.org/plosone/s/figures

You may also use PLOS’s free figure tool, NAAS, to help you prepare publication quality figures: https://journals.plos.org/plosone/s/figures#loc-tools-for-figure-preparation.

NAAS will assess whether your figures meet our technical requirements by comparing each figure against our figure specifications.

# Author comment: Author response to Decision Letter 1

**DOI:** 10.1371/journal.pone.0359540.r002

19 Feb 2026

Response sheet is attached.

Attachment Submitted filename: RESPONSE SHEET PLOS ONE.docx

# Peer review: Decision Letter 1

**DOI:** 10.1371/journal.pone.0359540.r003

**Authors:** Shahid Rahman

27 Mar 2026

Dear Dr. Ati,

Please submit your revised manuscript by May 11 2026 11:59PM. If you will need more time than this to complete your revisions, please reply to this message or contact the journal office at plosone@plos.org. When you're ready to submit your revision, log on to https://www.editorialmanager.com/pone/ and select the 'Submissions Needing Revision' folder to locate your manuscript file.

- A letter that responds to each point raised by the academic editor and reviewer(s). You should upload this letter as a separate file labeled 'Response to Reviewers'.
- A marked-up copy of your manuscript that highlights changes made to the original version. You should upload this as a separate file labeled 'Revised Manuscript with Track Changes'.
- An unmarked version of your revised paper without tracked changes. You should upload this as a separate file labeled 'Manuscript'.

If you would like to make changes to your financial disclosure, please include your updated statement in your cover letter. Guidelines for resubmitting your figure files are available below the reviewer comments at the end of this letter.

If applicable, we recommend that you deposit your laboratory protocols in protocols.io to enhance the reproducibility of your results. Protocols.io assigns your protocol its own identifier (DOI) so that it can be cited independently in the future. For instructions see: https://journals.plos.org/plosone/s/submission-guidelines#loc-laboratory-protocols. Additionally, PLOS ONE offers an option for publishing peer-reviewed Lab Protocol articles, which describe protocols hosted on protocols.io. Read more information on sharing protocols at https://plos.org/protocols?utm_medium=editorial-email&utm_source=authorletters&utm_campaign=protocols.

As the corresponding author, your ORCID iD is verified in the submission system and will appear in the published article. PLOS supports the use of ORCID, and we encourage all coauthors to register for an ORCID iD and use it as well. Please encourage your coauthors to verify their ORCID iD within the submission system before final acceptance, as unverified ORCID iDs will not appear in the published article. *Only* the individual author can complete the verification step; PLOS staff *cannot* verify ORCID iDs on behalf of authors.

We look forward to receiving your revised manuscript.

Kind regards,

Shahid Rahman, PhD

Academic Editor

PLOS One

Journal Requirements:

If the reviewer comments include a recommendation to cite specific previously published works, please review and evaluate these publications to determine whether they are relevant and should be cited. There is no requirement to cite these works unless the editor has indicated otherwise.

[Note: HTML markup is below. Please do not edit.]

Reviewers' comments:

Reviewer's Responses to Questions

**Comments to the Author**

Reviewer #1: (No Response)

Reviewer #2: All comments have been addressed

**********

2. Is the manuscript technically sound, and do the data support the conclusions?

Reviewer #1: Partly

Reviewer #2: Yes

**********

3. Has the statistical analysis been performed appropriately and rigorously?

Reviewer #1: No

Reviewer #2: Yes

**********

4. Have the authors made all data underlying the findings in their manuscript fully available?

The PLOS Data policy

Reviewer #1: Yes

Reviewer #2: Yes

**********

5. Is the manuscript presented in an intelligible fashion and written in standard English?

Reviewer #1: Yes

Reviewer #2: Yes

**********

Reviewer #1: This manuscript presents a texture-aware diffusion framework for generating synthetic future progression of diabetic foot ulcers (DFUs) from single images. The topic is timely and relevant, and the proposed integration of texture encoding with conditional diffusion modeling is technically interesting. The manuscript is well structured, and the experimental section includes ablation studies and both quantitative and qualitative evaluations. However, there are several major concerns that need to be addressed before the manuscript can be considered for publication.

First, the most critical issue lies in the definition of “progression prediction.” The dataset used in this study consists of independent single-timepoint images without true longitudinal follow-up. The proposed pseudo-progression strategy, where images are grouped into ordinal stages, does not provide genuine temporal supervision. As a result, the model does not truly learn progression dynamics but rather learns a synthetic ordering. This significantly weakens the core claim of predicting future ulcer progression.

Second, the manuscript makes strong clinical claims (e.g., clinical decision support and biologically realistic progression), which are not sufficiently supported by the presented validation. The evaluation is primarily based on image similarity metrics (SSIM, FID) and a limited expert review involving two specialists. While these are useful, they are insufficient to justify clinical applicability. More rigorous clinical validation or a clearer limitation discussion is required.

Third, the evaluation framework raises concerns. Since no ground-truth future images are available, the use of SSIM and FID does not directly measure predictive accuracy of progression. Instead, these metrics only assess similarity to the existing dataset distribution. This distinction should be clearly acknowledged and discussed.

Fourth, the dataset size (493 images) is relatively small for training a diffusion-based generative model, raising concerns about overfitting and generalization. Additional discussion or validation on external datasets would strengthen the work.

Fifth, the proposed metrics (Texture Consistency Score and Granulation Tissue Fidelity Score) appear to be newly introduced but lack sufficient validation or comparison with established evaluation standards. Their reliability and generalizability should be clarified.

Finally, several novelty claims (e.g., “first study”) appear overstated and should be carefully revised to avoid overclaiming.

In summary, while the proposed framework is technically sound and promising, the manuscript currently overstates its contributions and clinical implications. Addressing the issues related to progression definition, evaluation validity, and claim justification is necessary.

I recommend major revision.

Reviewer #2: 1. "Pseudo-Progression" Paradigm

In Section 3.1 (Phase 1: Data Collection), the authors explicitly state that longitudinal follow-up images were not included, and each image was treated as an independent case. To model progression, Section 3.5 introduces a "pseudo-progression paradigm" where independent images are grouped into ordinal stages based on severity.

Training a diffusion model on cross-sectional images sorted by severity effectively models disease severity interpolation across different patients, rather than true patient-specific temporal progression. While this is a clever workaround for data scarcity, calling it "future progression prediction" is clinically misleading.

The authors must explicitly discuss the physiological limitations of the "pseudo-progression" assumption in the Discussion section. It is highly recommended to reframe the narrative slightly to clarify that the model synthesizes "advanced severity states" rather than precise temporal forecasts, and to propose a concrete framework for how this model could be fine-tuned or validated on true longitudinal cohorts in the future.

2. Missing Quantitative Results from the Expert Clinical Review

In Section 3.6 (Phase 6: Evaluation and Validation), the methodology states that a qualitative validation was performed through an expert review study involving two board-certified wound care specialists. The experts reportedly evaluated 100 images using a 5-point Likert scale, and inter-rater reliability was measured using Cohen’s kappa coefficient.

Despite outlining this rigorous clinical validation protocol in the methodology, the quantitative results of this expert review are entirely absent from Section 5 (Experimental Results). The results section only presents automated metrics (SSIM, FID, TCS, GTFS).

Add a dedicated subsection and table in Section 5 detailing the outcomes of the clinical expert review. Include the mean Likert scores for morphological plausibility, textural accuracy, and clinical realism, as well as the exact Cohen’s kappa score to validate the clinical trustworthiness of the synthetic images.

3. Novel Evaluation Metrics

The paper introduces two highly specific metrics: Texture Consistency Score (TCS) and Granulation Tissue Fidelity Score (GTFS) to validate biological realism.

While Section 5.2 briefly mentions that TCS uses Local Binary Pattern (LBP) histogram analysis, there is no formal mathematical definition or algorithmic formulation provided for either TCS or GTFS in the Methodology (Section 3.6). Without explicit formulas or computation steps, peer researchers cannot reproduce these metrics or validate the claims made in Table 1 and Table 2.

Please, add the mathematical equations and a brief explanation of the algorithms used to compute TCS and GTFS in Section 3.6, similar to how the formulas for SSIM and FID were clearly defined.

4. Mitigating High-Frequency Texture Loss

In Section 5.4.3 and Figure 12, the authors candidly and accurately identify a limitation: the synthetic images suffer from over-smoothing and struggle to reproduce high-frequency microstructural details compared to the real zoom-in patches.

The paper leaves this critical flaw simply as an observation. Given that the entire premise of the paper is a "Texture-Aware" model, addressing this smoothing effect is paramount.

Expand the discussion around Figure 12 and Section 6. Suggest specific architectural or loss-function interventions to solve this. For example, discuss the potential of integrating a Focal Frequency Loss (to penalize missing high-frequency spectrums) or adding a high-pass filter within the adversarial loss block to strictly enforce micro-texture retention in future iterations.

**********

what does this mean?). If published, this will include your full peer review and any attached files.

If you choose “no”, your identity will remain anonymous but your review may still be made public.

**Do you want your identity to be public for this peer review?** For information about this choice, including consent withdrawal, please see our Privacy Policy

Reviewer #1: No

Reviewer #2: No

**********

[NOTE: If reviewer comments were submitted as an attachment file, they will be attached to this email and accessible via the submission site. Please log into your account, locate the manuscript record, and check for the action link "View Attachments". If this link does not appear, there are no attachment files.]

To ensure your figures meet our technical requirements, please review our figure guidelines: https://journals.plos.org/plosone/s/figures

You may also use PLOS’s free figure tool, NAAS, to help you prepare publication quality figures: https://journals.plos.org/plosone/s/figures#loc-tools-for-figure-preparation.

NAAS will assess whether your figures meet our technical requirements by comparing each figure against our figure specifications.

# Author comment: Author response to Decision Letter 2

**DOI:** 10.1371/journal.pone.0359540.r004

9 Jun 2026

The revision is done and manuscript is attached for consideration.

Attachment Submitted filename: RESPONSE SHEET REVISION 2 PLOS ONE.docx

# Peer review: Decision Letter 2

**DOI:** 10.1371/journal.pone.0359540.r005

**Authors:** Khan Bahadar Khan

29 Jul 2026

Dear Dr. Ati,

Thank you for submitting your manuscript to PLOS ONE. After careful consideration, we feel that it has merit but does not fully meet PLOS ONE’s publication criteria as it currently stands. Therefore, we invite you to submit a revised version of the manuscript that addresses the points raised during the review process.

Please submit your revised manuscript by Sep 12 2026 11:59PM. If you will need more time than this to complete your revisions, please reply to this message or contact the journal office at plosone@plos.org. When you're ready to submit your revision, log on to https://www.editorialmanager.com/pone/ and select the 'Submissions Needing Revision' folder to locate your manuscript file.

- A letter that responds to each point raised by the academic editor and reviewer(s). You should upload this letter as a separate file labeled 'Response to Reviewers'.
- A marked-up copy of your manuscript that highlights changes made to the original version. You should upload this as a separate file labeled 'Revised Manuscript with Track Changes'.
- An unmarked version of your revised paper without tracked changes. You should upload this as a separate file labeled 'Manuscript'.

If you would like to make changes to your financial disclosure, please include your updated statement in your cover letter. Guidelines for resubmitting your figure files are available below the reviewer comments at the end of this letter.

If applicable, we recommend that you deposit your laboratory protocols in protocols.io to enhance the reproducibility of your results. Protocols.io assigns your protocol its own identifier (DOI) so that it can be cited independently in the future. For instructions see: https://journals.plos.org/plosone/s/submission-guidelines#loc-laboratory-protocols. Additionally, PLOS ONE offers an option for publishing peer-reviewed Lab Protocol articles, which describe protocols hosted on protocols.io. Read more information on sharing protocols at https://plos.org/protocols?utm_medium=editorial-email&utm_source=authorletters&utm_campaign=protocols.

As the corresponding author, your ORCID iD is verified in the submission system and will appear in the published article. PLOS supports the use of ORCID, and we encourage all coauthors to register for an ORCID iD and use it as well. Please encourage your coauthors to verify their ORCID iD within the submission system before final acceptance, as unverified ORCID iDs will not appear in the published article. *Only* the individual author can complete the verification step; PLOS staff *cannot* verify ORCID iDs on behalf of authors.

We look forward to receiving your revised manuscript.

Kind regards,

Khan Bahadar Khan, Ph.D

Academic Editor

PLOS One

Journal Requirements:

1. If the reviewer comments include a recommendation to cite specific previously published works, please review and evaluate these publications to determine whether they are relevant and should be cited. There is no requirement to cite these works unless the editor has indicated otherwise.

2. Please review your reference list to ensure that it is complete and correct. If you have cited papers that have been retracted, please include the rationale for doing so in the manuscript text, or remove these references and replace them with relevant current references. Any changes to the reference list should be mentioned in the rebuttal letter that accompanies your revised manuscript. If you need to cite a retracted article, indicate the article’s retracted status in the References list and also include a citation and full reference for the retraction notice.

[Note: HTML markup is below. Please do not edit.]

Reviewers' comments:

Reviewer's Responses to Questions

**Comments to the Author**

Reviewer #1: All comments have been addressed

Reviewer #3: All comments have been addressed

**********

2. Is the manuscript technically sound, and do the data support the conclusions?

Reviewer #1: Partly

Reviewer #3: Yes

**********

3. Has the statistical analysis been performed appropriately and rigorously?

Reviewer #1: No

Reviewer #3: Yes

**********

4. Have the authors made all data underlying the findings in their manuscript fully available?

The PLOS Data policy

Reviewer #1: Yes

Reviewer #3: Yes

**********

5. Is the manuscript presented in an intelligible fashion and written in standard English?

Reviewer #1: Yes

Reviewer #3: Yes

**********

Reviewer #1: The authors have substantially addressed the major concerns raised in the previous round, particularly regarding the reframing of "future progression prediction" to "severity-conditioned progression-like synthesis," the removal of unsupported expert-review claims, the addition of formal TCS/GTFS definitions, and the expanded discussion of texture over-smoothing limitations. The manuscript is now considerably more careful about the scope of its claims. However, a few issues remain before the manuscript can be accepted:

1. There is an unexplained discrepancy between the SSIM/FID values reported in Table 3 (SSIM 0.82 ± 0.03, FID 31.4) and Table 5 (SSIM 0.851 ± 0.020, FID 44.33 ± 5.02), both attributed to the same full proposed diffusion model. Please clarify the source of this difference (e.g., different aggregation method, different subset of results) or correct the inconsistency.

2. The description of how pseudo-progression stage labels were assigned is inconsistent across sections (automated visual-cue-based grouping in Section 3.5 vs. "assigned by experts" in the Conclusion). Please clarify the exact labeling procedure and, if any manual/expert input was involved, provide details on the annotators and protocol.

3. The Granulation Tissue Fidelity Score (Eq. 18) relies on a "granulation-related appearance map" G(I) whose derivation is not specified. Please describe how this map is computed (e.g., color-space thresholding, a trained segmentation model) to allow reproducibility.

4. Given the small test set (n=74) and the explicit absence of formal significance testing (Section 5.10), the language describing the diffusion model's advantage over GAN/VAE baselines (e.g., "clear and consistent advantage") should be softened, or a suitable statistical test/bootstrap confidence interval should be added to support the comparison.

5. Minor language/formatting issues remain (duplicated figure captions/text blocks, some repetitive phrasing) that should be cleaned up at copyediting.

These are relatively minor points given the substantial improvements already

made, and I recommend minor revision.

Reviewer #3: Thank you very much for addressing all the comments from all the reviewers. The manuscript is now good.

**********

what does this mean?). If published, this will include your full peer review and any attached files.

If you choose “no”, your identity will remain anonymous but your review may still be made public.

**Do you want your identity to be public for this peer review?** For information about this choice, including consent withdrawal, please see our Privacy Policy

Reviewer #1: No

Reviewer #3: No

**********

[NOTE: If reviewer comments were submitted as an attachment file, they will be attached to this email and accessible via the submission site. Please log into your account, locate the manuscript record, and check for the action link "View Attachments". If this link does not appear, there are no attachment files.]

To ensure your figures meet our technical requirements, please review our figure guidelines: https://journals.plos.org/plosone/s/figures

You may also use PLOS’s free figure tool, NAAS, to help you prepare publication quality figures: https://journals.plos.org/plosone/s/figures#loc-tools-for-figure-preparation.

NAAS will assess whether your figures meet our technical requirements by comparing each figure against our figure specifications.

# Author comment: Author response to Decision Letter 3

**DOI:** 10.1371/journal.pone.0359540.r006

28 Aug 2026

Revision file is attached

Attachment Submitted filename: RESPONSE SHEET DFU REVISION 3.docx

# Peer review: Decision Letter 3

**DOI:** 10.1371/journal.pone.0359540.r007

**Authors:** Khan Bahadar Khan

15 Sep 2026

Texture-Aware Diffusion Modelling for Severity-Conditioned Synthetic Progression-Like Visualization of Diabetic Foot Ulcers from Single Images

PONE-D-25-41148R3

Dear Dr. Ati,

We’re pleased to inform you that your manuscript has been judged scientifically suitable for publication and will be formally accepted for publication once it meets all outstanding technical requirements.

Within one week, you’ll receive an e-mail detailing the required amendments. When these have been addressed, you’ll receive a formal acceptance letter and your manuscript will be scheduled for publication.

An invoice will be generated when your article is formally accepted. Please note, if your institution has a publishing partnership with PLOS and your article meets the relevant criteria, all or part of your publication costs will be covered. Please make sure your user information is up-to-date by logging into Editorial Manager at Editorial Manager® and clicking the ‘Update My Information' link at the top of the page. For questions related to billing, please contact billing support.

If your institution or institutions have a press office, please notify them about your upcoming paper to help maximize its impact. If they’ll be preparing press materials, please inform our press team as soon as possible -- no later than 48 hours after receiving the formal acceptance. Your manuscript will remain under strict press embargo until 2 pm Eastern Time on the date of publication. For more information, please contact onepress@plos.org.

Kind regards,

Khan Bahadar Khan, Ph.D

Academic Editor

PLOS One

Additional Editor Comments (optional):

Reviewers' comments:

Reviewer's Responses to Questions

**Comments to the Author**

Reviewer #1: All comments have been addressed

Reviewer #3: All comments have been addressed

**********

2. Is the manuscript technically sound, and do the data support the conclusions?

Reviewer #1: Yes

Reviewer #3: Yes

**********

3. Has the statistical analysis been performed appropriately and rigorously?

Reviewer #1: Yes

Reviewer #3: Yes

**********

4. Have the authors made all data underlying the findings in their manuscript fully available?

The PLOS Data policy

Reviewer #1: Yes

Reviewer #3: Yes

**********

5. Is the manuscript presented in an intelligible fashion and written in standard English?

Reviewer #1: Yes

Reviewer #3: Yes

**********

Reviewer #1: I have verified directly in the revised manuscript that:

(1) the SSIM/FID values in Table 3 are now numerically consistent with Table 5 and Table 7;

(2) the pseudo-progression stage labeling procedure is now consistently described as a predefined visual-cue-based heuristic rather than expert-assigned labels, both in Section 3.5 and the Conclusion;

(3) the Granulation Tissue Fidelity Score's underlying appearance map is now explicitly derived via a CIELAB-based soft appearance mapping procedure; and

(4) comparative language regarding model performance has been appropriately softened to reflect the absence of formal statistical significance testing.

The manuscript now presents its contributions and limitations with appropriate scientific caution, clearly distinguishing "progression-like synthetic visualization" from genuine patient-specific temporal prediction. I have no further concerns and recommend acceptance.

Reviewer #3: Thank you for submitting the revised version of your work, “Texture-Aware Diffusion Modelling for Severity-Conditioned Synthetic Progression-Like Visualization of Diabetic Foot Ulcers from Single Image”.

All reviewers' comments have been addressed

**********

what does this mean?). If published, this will include your full peer review and any attached files.

If you choose “no”, your identity will remain anonymous but your review may still be made public.

**Do you want your identity to be public for this peer review?** For information about this choice, including consent withdrawal, please see our Privacy Policy

Reviewer #1: No

Reviewer #3: **Yes:**  Mohammed Abdo Mahdi

**********

# Editor report: Acceptance letter

**DOI:** 10.1371/journal.pone.0359540.r008

**Authors:** Khan Bahadar Khan

PONE-D-25-41148R3

PLOS One

Dear Dr. Ati,

I'm pleased to inform you that your manuscript has been deemed suitable for publication in PLOS One. Congratulations! Your manuscript is now being handed over to our production team.

At this stage, our production department will prepare your paper for publication. This includes ensuring the following:

* All references, tables, and figures are properly cited

* All relevant supporting information is included in the manuscript submission,

* There are no issues that prevent the paper from being properly typeset

You will receive further instructions from the production team, including instructions on how to review your proof when it is ready. Please keep in mind that we are working through a large volume of accepted articles, so please give us a few days to review your paper and let you know the next and final steps.

Lastly, if your institution or institutions have a press office, please let them know about your upcoming paper now to help maximize its impact. If they'll be preparing press materials, please inform our press team within the next 48 hours. Your manuscript will remain under strict press embargo until 2 pm Eastern Time on the date of publication. For more information, please contact onepress@plos.org.

You will receive an invoice from PLOS for your publication fee after your manuscript has reached the completed accept phase. If you receive an email requesting payment before acceptance or for any other service, this may be a phishing scheme. Learn how to identify phishing emails and protect your accounts at https://explore.plos.org/phishing.

If we can help with anything else, please email us at customercare@plos.org.

Thank you for submitting your work to PLOS One and supporting open access.

Kind regards,

PLOS One Editorial Office Staff

on behalf of

Dr. Khan Bahadar Khan

Academic Editor

PLOS One
